# Boosting for high-dimensional two-class prediction

**DOI:** 10.1186/s12859-015-0723-9

**Published:** 2015-09-21

**Authors:** Rok Blagus, Lara Lusa

**Affiliations:** Institute for Biostatistics and Medical Informatics, University of Ljubljana, Vrazov trg 2, Ljubljana, Slovenia

**Keywords:** Class-prediction, Boosting, AdaBoost.M1, Gradient boosting, Stochastic Gradient boosting, LogitBoost

## Abstract

**Background:**

In clinical research prediction models are used to accurately predict the outcome of the patients based on some of their characteristics. For high-dimensional prediction models (the number of variables greatly exceeds the number of samples) the choice of an appropriate classifier is crucial as it was observed that no single classification algorithm performs optimally for all types of data. Boosting was proposed as a method that combines the classification results obtained using base classifiers, where the sample weights are sequentially adjusted based on the performance in previous iterations. Generally boosting outperforms any individual classifier, but studies with high-dimensional data showed that the most standard boosting algorithm, AdaBoost.M1, cannot significantly improve the performance of its base classier. Recently other boosting algorithms were proposed (Gradient boosting, Stochastic Gradient boosting, LogitBoost); they were shown to perform better than AdaBoost.M1 but their performance was not evaluated for high-dimensional data.

**Results:**

In this paper we use simulation studies and real gene-expression data sets to evaluate the performance of boosting algorithms when data are high-dimensional. Our results confirm that AdaBoost.M1 can perform poorly in this setting, often failing to improve the performance of its base classifier. We provide the explanation for this and propose a modification, AdaBoost.M1.ICV, which uses cross-validated estimates of the prediction errors and outperforms the original algorithm when data are high-dimensional. The use of AdaBoost.M1.ICV is advisable when the base classifier overfits the training data: the number of variables is large, the number of samples is small, and/or the difference between the classes is large. To a lesser extent also Gradient boosting suffers from similar problems. Contrary to the findings for the low-dimensional data, shrinkage does not improve the performance of Gradient boosting when data are high-dimensional, however it is beneficial for Stochastic Gradient boosting, which outperformed the other boosting algorithms in our analyses. LogitBoost suffers from overfitting and generally performs poorly.

**Conclusions:**

The results show that boosting can substantially improve the performance of its base classifier also when data are high-dimensional. However, not all boosting algorithms perform equally well. LogitBoost, AdaBoost.M1 and Gradient boosting seem less useful for this type of data. Overall, Stochastic Gradient boosting with shrinkage and AdaBoost.M1.ICV seem to be the preferable choices for high-dimensional class-prediction.

**Electronic supplementary material:**

The online version of this article (doi:10.1186/s12859-015-0723-9) contains supplementary material, which is available to authorized users.

## Background

The goal of class prediction (classification) is to develop a rule (classifier) based on the values of the variables measured on a group of samples with known class membership (training set). This rule can be used to predict the class membership for samples with unknown class membership but for which the values of the variables used by the rule are known [1]. In clinical research prediction models are used to develop rules that can be used to accurately predict the outcome of the patients based on some of their characteristics and are extensively used in medicine and bioinformatics [2–4]. They represent a valuable tool in the decision making process of clinicians and health policy makers, as they enable them to estimate the probability that patients have or will develop a disease, will respond to a treatment, or that their disease will recur. For example, the use of mass spectrometry to develop profiles of patient serum proteins, could lead to early detection of ovarian cancer, which has the potential to reduce mortality [5, 6].

The new technological advances allow the biomedical researchers to measure the expression of ten thousands of genes, or over one million single nucleotide polymorphisms (SNPs), in a single assay. These technologies are increasingly often used with the aim to develop personalized treatments or individualized drug selection [7, 8]. For this reason class prediction studies in biomedicine are increasingly often high-dimensional: the number of variables (features) greatly exceeds the number of samples (see for example [9–13]), posing important methodological challenges. For high-dimensional predictive models the choice of an appropriate classifier, and its correct analytical validation, is crucial. For example, when several research groups were invited to build predictive models for breast cancer diagnosis based on proteomic mass spectrometry data, it was shown that no single classification algorithm performs optimally for all types of data [14]. In the current practice the genetic-based predictive modeling of common diseases is still disappointing [15].

Ensemble classifiers are combinations of many different classifiers whose outputs are combined into a single vote by majority voting or weighted majority voting. They can be useful because combining a set of classifiers can result in more accurate predictions [16]. Recently, ensemble of genetic models based on genome-wide association studies were shown to have an increased sensitivity compared to individual models, while their lower specificity appeared to affect minimally their predictive accuracy [17]. Bagging (Bootstrap Aggregating) [18] and boosting [19] are two of the most popular approaches for generating ensemble classifiers [20]. The classifiers used to construct the ensemble (base classifiers) are usually selected among classifiers that perform a little bit better than a random guess (weak classifiers). In this paper we focus on boosting, which sequentially applies the base classifier to repeatedly modified versions of the data, producing a sequence of classifiers whose final class assignment is determined by weighted majority voting. Boosting was shown to improve the accuracy of the base classifier and reduce its variance when applied to low-dimensional data [21].

Boosting was applied to high-dimensional data, using mostly gene expression data, by Ben-Dor et al. [22] and Dudoit et al. [23], concluding that the most standard boosting algorithm, AdaBoost.M1 [19, 24], does not perform well for high-dimensional data. In a more recent study, Stollhoff et al. [25], compared AdaBoost.M1 and logistic regression and concluded that while boosting of simple base classifiers can give classification rules with improved predictive ability, the logistic regression model remains a method of choice in the medical applications, since the performance of boosting classifiers was not generally superior to the performance of logistic regression. Regardless of the findings presented by [22] and [23], boosting is very popular in bioinformatics and it is often used also for high-dimensional class prediction. For example, boosting was used to predict protein disorders [26], for classifying the output from an *in silico* vaccine discovery pipeline for eukaryotic pathogens [27] or MeSH indexing based on automatically generated summaries [28].

Dettling and Bühlmann [29] proposed a boosting approach that combined a dimensionality reduction step with LogitBoost [30] and compared it to AdaBoost.M1, nearest neighbor classifier and classification and regression trees (CART) [31] in the context of tumor classification with gene expression data. The authors used LogitBoost as it was shown that for low-dimensional data it can perform slightly better than AdaBoost.M1 when the signal-to-noise ratio is small [29, 30], which is often the case with gene expression data. They used real high-dimensional data to show that their approach can outperform the other classifiers in some datasets. The presented studies however did not provide an explanation for the poor performance of AdaBoost.M1 with high-dimensional data nor did they consider other state-of-the-art boosting algorithms.

AdaBoost.M1 was shown to be equivalent to forward stagewise additive modeling using the exponential loss function [30]. Based on this finding Gradient boosting (GrBoost [32]) and Stochastic Gradient boosting (St-GrBoost, [33]) were proposed. Both techniques were demonstrated to perform well with low-dimensional data and were applied also to high-dimensional data in the context of generalized linear models [34] and generalized additive models for location, scale and shape [35]. These studies showed that gradient boosting algorithms can perform well also with high-dimensional data, however they were not systematically evaluated specifically within the class prediction framework.

In this paper we investigate how boosting algorithms are affected by high-dimensionality, limiting our interest to two-class prediction problems and class-balanced data, i.e. a situation where the number of samples from both classes is approximately the same. We first show that when it is easy to overfit the training data with the base classifier, AdaBoost.M1 and GrBoost algorithms perform exactly as their base classifiers, which can explain the poor performance of AdaBoost.M1 presented in [22, 23, 29]. We show that the proneness to overfitting data is related to the number of variables, the number of samples and the magnitude of the between class difference. Based on this finding we propose a modification of the AdaBoost.M1 algorithm and demonstrate that it outperforms the original approach when data are prone to overfitting, and performs similarly otherwise. The performance of boosting algorithms is evaluated using simulated data where we investigate the effect of the size of the training set, the magnitude of the between class difference and the number of boosting iterations. The results from our simulations are validated by using gene expression microarray datasets. Throughout the analysis we use classification and regression trees (CART) as base classifiers.

## Methods

### Classifiers

We evaluated the performance of AdaBoost.M1, gradient boosting (GrBoost), LogitBoost and of AdaBoost.M1.ICV, an algorithm that we propose in this paper, on high-dimensional data. The base classifiers were classification trees where maximum depth was set to 5 (CART(5)) or 1 (decision stump, CART(1)). The acronyms used to indicate the specific boosting algorithms are reported in Table 1, where some additional details are given. Full details about the algorithms are given in Additional file 1, here we outline only the aspects of the algorithms that are relevant for the understanding of the results.

**Table 1:** Short description of the classifiers used in the paper

Name	Base classifier	Boosting method	Number of boosting iterations^a^
CART(1)	Stump	-	-
CART(5)	CART-5	-	-
AdaBoost.M1(1)	Stump	AdaBoost.M1	10, 100, 200, 300
AdaBoost.M1(5)	CART-5	AdaBoost.M1	10, 100, 200, 300
AdaBoost.M1.ICV(5)	CART-5	AdaBoost.M1^b^	10,100, 200, 300
GrBoost(1)	Stump	Gradient Boosting	10, 100, 200, 300
GrBoost(5)	CART-5	Gradient Boosting	10, 100, 200, 300
ST-GrBoost(1)	Stump	Gradient Boosting^c^	100, 300, 500, optimal^d^
ST-GrBoost(5)	CART-5	Gradient Boosting^c^	100, 300, 500, optimal^d^
LogitBoost(1)	Stump	LogitBoost	10, 100, 200, 300

^a^This is the number of boosting iterations considered when evaluating the effect of the sample size and between class difference and in the reanalysis of real data. In the other simulation settings up to 1000 iterations were considered for each classifier

^b^Cross-validated error rate was used to update the weights

^c^In each boosting iteration 50 % of training set samples are randomly selected and used to fit the model

^d^Optimal number of boosting iterations based on out-of-bag estimate

**Fig. 1:**
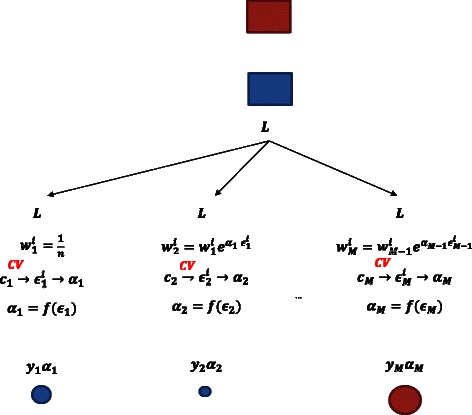
Schematic presentation of the AdaBoost.M1.ICV. With *L* we denote the training set, *n* is the size of the training set, *m* denotes the boosting iteration (*m*=1,…,*M*). For the *m*-th boosting iteration: ${w_{m}^{i}}$ are the case-specific weights for sample *i*, *c*_*m*_ is a base classifier, ${\epsilon _{m}^{i}}$ is the cross-validated error for sample *i*, *ε*_*m*_ is the cross-validated error, *α*_*m*_ denotes the classifier-specific weight and *y*_*m*_ is predicted class for a new sample at iteration *m*

Briefly, in AdaBoost.M1 at each boosting iteration the weights applied to the training observations depend on the training (re-substitution) error [20]; samples that are misclassified get larger weights and therefore the classifier focuses more on these samples in the next boosting iteration. We propose to estimate the error rate achieved by the base classifier at each boosting iteration with internal cross-validation (CV), and then use the cross-validated error rate to update the weights; the rest of the algorithm is the same as for AdaBoost.M1. A schematic presentation of the new approach, which is denoted as AdaBoost.M1.ICV, is presented in Fig. 1 and the algorithm is presented in the Additional file 1. In our analyses the internal cross-validation was performed using 5 folds.

Exponential loss was used for gradient boosting and we investigated the performance of two regularization strategies: shrinkage and sub-sampling. With sub-sampling at each boosting iteration a fraction *η* of the training data is sampled without replacement and the tree is grown using only that subsample: this approach is known as Stochastic Gradient boosting (St-GrBoost). In our simulations *η* was set to 0.5 [20]. With shrinkage the contribution of each base classifier is scaled by a factor (0<*ν*<1, *ν*=1 is no shrinkage, in our analyses the shrinkage factor was set to *ν*=0.001, 0.01, 0.1 and 1). In the low-dimensional setting it was suggested that *ν* should be set to a very small number and than one should choose the number of iterations (M) by early stopping [20]. However, smaller values of *ν* (more shrinkage) lead to larger number of boosting iterations for the same training error, so there is a trade-off between them, which can be an issue in the high-dimensional setting because of larger computational burden.

### Simulations

The simulations were performed to evaluate the performance of different boosting algorithms on high-dimensional data.

The class membership of the samples depended on some of the variables. The variables were simulated from a multivariate normal distribution, $\text {MVN}(\underline {\mathbf {\mu }_{k}},\mathbf {\Sigma })$, with $\underline {\mathbf {\mu }_{1}}=(0,\ldots,0)^{T}$ for class 1 samples and $\underline {\mathbf {\mu }_{2}}=(\mu _{2},\ldots,\mu _{2},0,\ldots,0)^{T}$ for class 2 samples; there were 100 differentially expressed variables and the variance of all variables was set to 1. The variables were grouped in blocks of size 10: the variables within the same block were correlated (*ρ*=0.8), while those from different blocks were independent (similarly as in [36, 37]). The number of simulated variables is denoted as *p*, while the number of samples in the training set is denoted as *n*_*train*_. In all settings the number of class 1 and class 2 samples was the same.

The performance of the classifiers in all simulation settings was evaluated on independent test sets containing 500 samples from each class and the results were reported as averages from 100 iterations. Next, we give the exact simulation settings for each subsection appearing in the “Results” section.

#### Performance of AdaBoost.M1 in the high-dimensional setting

The number of variables was set to *p*=1000, the number of training set samples was *n*_*train*_=50 and the difference between the classes was *μ*_2_=0.7. We considered up to *M*=1000 boosting iterations.

We performed also a set of simulations used to illustrate the effect of high-dimensionality on the overfitting of CART(5) and CART(1). We simulated independent standard normal variables (*ρ*=0), the number of variables ranged from 10 to 10000 and the size of the training set ranged from 50 to 1000. There was no difference between the classes, *μ*_2_=0. We evaluated the resubstitution error rate of CART(5) and CART(1).

#### Effect of shrinkage on Gradient boosting and Stochastic Gradient boosting

The number of variables was set to *p*=100, 1000 and 10000. The number of training set samples was *n*_*train*_=50, 100 and 500 for each *p* and the difference between the classes was *μ*_2_=0.7. We considered up to *M*=1000 boosting iterations. The smallest test set error obtained over 1000 boosting iterations was averaged over 100 simulation runs; the test set error as a function of the number of boosting iterations averaged over 100 simulation runs is provided as Additional information.

#### Boosting in the high-dimensional setting with small samples: test set error as a function of the number of boosting iterations

The number of variables was set to *p*=100, 1000 and 10000, the number of training set samples to *n*_*train*_=50 and the difference between the classes to *μ*_2_=0.7. We considered up to *M*=1000 boosting iterations.

We performed also a set of simulations where we adjusted the differences between the classes so as to achieve approximately the same test set errors using CART(5) for all values of *p*: this was achieved increasing the mean difference between classes when the number of variables was larger (*μ*_2_=0.7 for *p*=100, *μ*_2_=0.8 for *p*=1000 and *μ*_2_=1 for *p*=10000). These results are reported as Additional information.

#### Effect of the sample size, number of variables and between class difference

The number of samples in the training set was set to *n*_*train*_= 50, 100, 200, 500 and 1000. For each of these settings the number of variables was set to *p*=1000 and 2500, while for each *n*_*train*_ and *p* combination the difference between the classes was set to *μ*_2_= 0.5 or 1.

To reduce the computing time, the number of boosting iterations in each ensemble was set to 10, 100, 200 or 300; additionally we considered also 500 iterations as well as the optimal number of boosting iterations based on the out-of-bag estimate for Stochastic Gradient boosting.

#### Simulation setup with a complex separation between the classes

In this setting the mean for all variables for both classes was equal, while the variability of some variables was different in the two classes. This enabled us to simulate a data structure were samples from one class were nested within the samples from the other class. We simulated 980 or 9980 variables from MVN((50,…,50)^*T*^,*d* *i* *a* *g*(12.5,…,12.5)), while the 20 differentially expressed variables were simulated from MVN((50,…,50)^*T*^,*d* *i* *a* *g*(12.5,…,12.5)) for 50 class 1 samples and MVN((50,…,50)^*T*^,*d* *i* *a* *g*(3.125,…,3.125)) for 50 class 2 samples. Up to *M*= 500 boosting iterations were considered.

#### Determining the number of boosting iterations with cross-validation

The number of variables was set to *p*=1000, the number of training set samples was *n*_*train*_=50 and the difference between the classes was *μ*_2_=0.7.

We used 5-fold cross-validation to determine the cross-validated number of iterations, i.e. the training set was split into 5 folds, 4 folds were used to train the classifiers using *M*=1000 boosting iterations, while the left-out fold was used to estimate the classification error for each of the *M*=1,…,1000 iterations. The 5-fold cross-validated number of iterations was defined as the number of iterations where the cross-validated error rate (evaluated using the left out samples) was the smallest. In case of ties we selected the smallest number of iterations. Similarly, we determined the leave-one-out cross-validated number of iterations, where exactly one sample was included in each of the left out folds. Additionally, we report also the optimal number of iterations, which is defined as the number of iterations where the minimum test set error rate (optimal error rate) is achieved over the 1000 boosting iterations.

### Real data

We reanalyzed the breast cancer microarray gene expression data of Sotirou et al. [38], Wang et al. [39] and Ivshina et al. [40] considering the prediction of Estrogen receptor status (ER; all datasets), grade of the tumor (Grade; Ivshina and Sotiriou datasets) and relapse of the tumor (Wang dataset), see also Table 2. The data were preprocessed as described in the original publications. Missing data were present in the cDNA two-channel dataset [38]: the genes with more than 10 % of missing values were removed from the analysis, the remaining missing values were replaced with zeros. The 1000 variables exhibiting the largest variance were pre-filtered and used for further analysis. 5-fold CV was used to estimate the accuracy measures. The settings for CART and the ensemble classifiers were the same as in the simulation study. In order to account for the variability arising from random inclusion of samples in different folds the analysis was repeated 50 times and average results and standard deviations are reported. Since some dataset and/or prediction tasks exhibit high level of class-imbalance we adjusted for the possible class-imbalance bias [41] by down-sizing the majority class, i.e. in each cross-validation run, min(*n*_*min*_,*n*_*max*_) samples from the majority class were selected and included in the training set. This strategy was shown to perform well with high-dimensional data [42].

**Table 2:** Gene expression breast cancer data sets

Data set	# genes	Classification task	*n*_*min*_	*n*_*max*_	*k*_*min*_
		(minority vs. majority class)			
Ivshina	22,283	ER- or ER+	34	211	0.14
		Grade 3 or 1-2	55	234	0.19
Wang	22,283	ER- or ER+	77	209	0.27
		Relapse or no-relapse	107	179	0.37
Sotiriou	7,650	ER- or ER+	34	65	0.34
		Grade 3 or 1-2	45	54	0.45

### Evaluation of the performance of the classifiers

Five measures of classifier’s performance were considered: (i) overall predictive accuracy (PA, the number of correctly classified samples from the test set divided by the total number of samples in the test set), (ii) predictive accuracy of class 1 (PA_1_, i.e., PA evaluated using only samples from class 1), (iii) predictive accuracy of class 2 (PA_2_), (iv) Area Under the Receiver-Characteristic-Operating Curve (AUC) [43] and (v) g-means (defined as geometric average of class-specific predictive accuracies, i.e. $\text {g-means}=\sqrt {\text {PA}_{1}\cdot \text {PA}_{2}}$).

We used Wilcoxon signed-rank test to test if there was a statistically significant difference between g-means obtained with AdaBoost.M1.ICV(5) and the other classifiers across various different simulation settings (24 in total). For each classifier, the number of boosting iterations that achieved the best classification performance in terms of g-means was considered. Because of a large number of comparisons the p-values were adjusted with the Holm’s method to control the type I error [44]. An adjusted p-value of less than 0.05 was considered as statistically significant. We did not perform any statistical tests for the results obtained with the analysis of the real datasets, as there were only 6 data points for each classifier.

### Computational aspects

All the analyses were performed with R language for statistical computing, version 3.0.0 [45]. The function *LogitBoost* in package caTools was used to perform LogitBoost, functions *gbm.fit* and *gbm.perf* from gbm package to perform GrBoost and St-GrBoost, while the other ensemble classifiers were programmed in R by the authors; the R-code is available upon request.

## Results

In this section we present the results based on our simulation studies and the reanalysis of microarray gene expression data. The complete simulation settings appear in the “Methods” section (see sections with matching titles).

### Performance of AdaBoost.M1 in the high-dimensional setting

Here we report a selected series of simulation results used to illustrate the performance of AdaBoost.M1 when the data are high-dimensional (Table 3).

**Table 3:** Test-set error for different classifiers and number of boosting iterations. The table displays the test set error averaged over 100 simulation runs for different classifiers and the number of boosting iterations (M; the situation with M =1 is the performance of the base classifier) for the setting with 1000 variables and 50 samples. The difference between the classes was moderate, the correlation structure was exchangeable and there were 10 variables per block, see the “Methods” section for more details

M	AdaBoost.M1(5)	AdaBoost.M1(1)	AdaBoost.M1.ICV(5)
1	0.38	0.38	0.38
100	0.38	0.27	0.31
500	0.38	0.26	0.27
1000	0.38	0.26	0.26

AdaBoost.M1 with CART(5) (AdaBoost.M1(5)) performed the same as its base classifier; increasing the number of boosting iterations did not decrease the error on the independent test set. On the contrary, AdaBoost.M1 with stumps (AdaBoost.M1(1)) improved the performance of its base classifier and the test error substantially decreased when combining more classifiers (increasing the number of boosting iterations).

We identified the reason for the inability of AdaBoost.M1(5) to improve the performance of its base classifier in the mechanism used to obtain modified versions of the data to which the base classifier is applied. Recall that the weights applied to the training observations depend on the re-substitution error (see the “Methods” section and Additional file 1 for more details). The resubstitution error of the base classifier drops to zero when the data proneness to overfitting is large [20]; in this case the weights in AdaBoost.M1 are not updated and all subsequent base classifiers produce the same prediction results. In this simulation setting the average test set error of CART(5) was large (around 0.37) but its resubstitution error rate was zero.

**Fig. 2:**
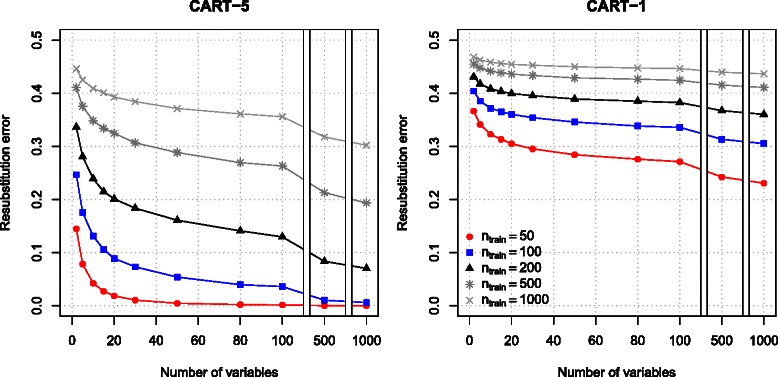
Resubstitution error rate of CART(5) (*left panels*) and stumps (CART(1), *right panels*). The figure reports mean resubstitution error rate of CART(5) and CART(1) for different number of variables and size of the training set (*n*_*train*_). The true error rate in this setting is 0.5. The results are based on 1000 simulations

We illustrate how the overfitting of CART depends on the high-dimensionality in a setting when there is no difference between the classes (test set error should be 0.5). The resubstitution error rate of CART(5) dropped to zero when the number of variables increased; zero resubstitution error rate was achieved faster when the sample size was smaller (Fig. 2; exact numerical results are reported in Additional file 2). We experimentally observed that larger trees (depth larger than 5) achieved a zero resubstitution error rate at an even smaller *p* to *n* ratio (data not shown). The error rate of stumps was non-zero even when the number of variables was large, however it was substantially smaller than the true error rate for this setting (0.5).

These results indicate that AdaBoost.M1(5) could perform better than its base classifier when the sample size is larger, as the achieved resubstitution error rate is less frequently zero. More results about the effect of the sample size appear later in the paper.

To avoid the problem arising from zero resubstitution error we propose to estimate the error rate achieved by the base classifier at each boosting iteration with cross-validation (AdaBoost.M1.ICV). This modification of the weights in the AdaBoost.M1 algorithm substantially improved its performance when using CART(5) as a base classifier (Table 3), while it performed similarly as AdaBoost.M1(1). Note that in this simulation setting the test set error of CART(5) and stumps was very similar (see the results for M =1 for AdaBoost.M1(1) and AdaBoost.M1(5), respectively, Table 3). Examples were AdaBoost.M1.ICV clearly outperforms AdaBoost.M1(1) appear later in the paper.

### Effect of shrinkage on Gradient boosting and stochastic Gradient boosting

Gradient boosting (GrBoost) has the potential of performing better than AdaBoost.M1 in our settings, since it produces classifiers that are not identical to its base classifiers, even when the training error of the base classifier is zero (see Additional file 1 for more details). In this section we present the results of the simulations performed with the aim to determine how much the classification results are affected by shrinkage, considering different values of the shrinkage parameter.

Similarly to AdaBoost.M1, when we used CART(5) with small samples (*n*_*train*_=50; Table 4). Gradient boosting performed similarly to its base classifier, regardless of the amount of shrinkage (Additional file 3). In the other situations (*n*_*train*_>50) Gradient boosting generally performed substantially better without shrinkage (*ν*=1).

**Table 4:** Test-set error for Gradient boosting and Stochastic Gradient boosting with different shrinkage parameter. In the table we report the averaged smallest test set error obtained over 1000 boosting iterations for different shrinkage parameter (*ν*), size of the training set (*n*_*train*_) and number of variables (*p*); see text for more details

		Gradient boosting								Stochastic Gradient boosting							
		CART(5)				CART(1)				CART(5)				CART(1)			
		*ν*				*ν*				*ν*				*ν*			
*n*_*train*_	*p*	0.001	0.01	0.1	1	0.001	0.01	0.1	1	0.001	0.01	0.1	1	0.001	0.01	0.1	1
50	100	0.31	0.31	0.30	**0.28**	0.30	0.23	0.21	**0.20**	0.20	0.19	0.20	**0.17**	0.22	0.18	0.17	**0.17**
50	1000	0.36	0.36	0.37	**0.36**	0.32	0.29	0.29	**0.27**	0.24	0.23	0.27	**0.22**	0.26	**0.24**	0.24	0.24
50	10000	0.40	0.41	**0.40**	0.41	0.38	0.37	0.37	**0.36**	0.30	0.30	0.34	**0.26**	0.33	0.33	**0.33**	0.34
100	100	0.26	0.21	0.19	**0.17**	0.29	0.20	0.18	**0.17**	0.17	0.16	0.16	**0.15**	0.21	0.16	**0.15**	0.16
100	1000	0.31	0.24	0.23	**0.21**	0.29	0.22	0.22	**0.21**	0.21	**0.19**	0.21	0.19	0.23	**0.19**	0.19	0.20
100	10000	0.36	0.31	0.28	**0.26**	0.30	0.27	0.27	**0.25**	0.24	**0.23**	0.27	0.25	0.25	**0.23**	0.24	0.27
500	100	0.20	0.14	0.14	**0.14**	0.24	0.15	**0.14**	0.15	0.15	**0.13**	0.13	0.14	0.19	0.13	**0.13**	0.15
500	1000	0.20	0.15	0.15	**0.14**	0.25	0.15	**0.15**	0.16	0.15	**0.13**	0.14	0.15	0.20	**0.14**	0.14	0.16
500	10000	0.21	0.16	0.15	**0.15**	0.25	0.15	**0.15**	0.16	0.16	**0.14**	0.14	0.15	0.20	**0.14**	0.15	0.17

The smallest test-set error of the classifier achieved with different amount of shrinkage is denoted in bold

Shrinkage seemed more useful with Stochastic Gradient boosting, especially when the sample size was large. In most settings the best results were obtained with *ν*=0.01. However, the results obtained with different values of the shrinkage parameter were very similar, especially when the number of boosting iterations was large (Additional file 3). When we did not use shrinkage, we observed some overfitting, i.e. after a certain number of boosting iterations the test set error tended to increase, but this was limited to situations with a large number of variables and small training sets.

Based on these results we decided that in all the subsequent simulations we would not use shrinkage with Gradient boosting and use *ν*=0.01 for Stochastic Gradient boosting. Note however, that it might be possible, that some other values of *ν* could lead to better performance in some simulation settings, suggesting that the shrinkage parameter should be estimated from the data by cross-validation, which does, however, substantially increase computation time.

### Boosting in the high-dimensional setting with small samples: test set error as a function of the number of boosting iterations

**Fig. 3:**
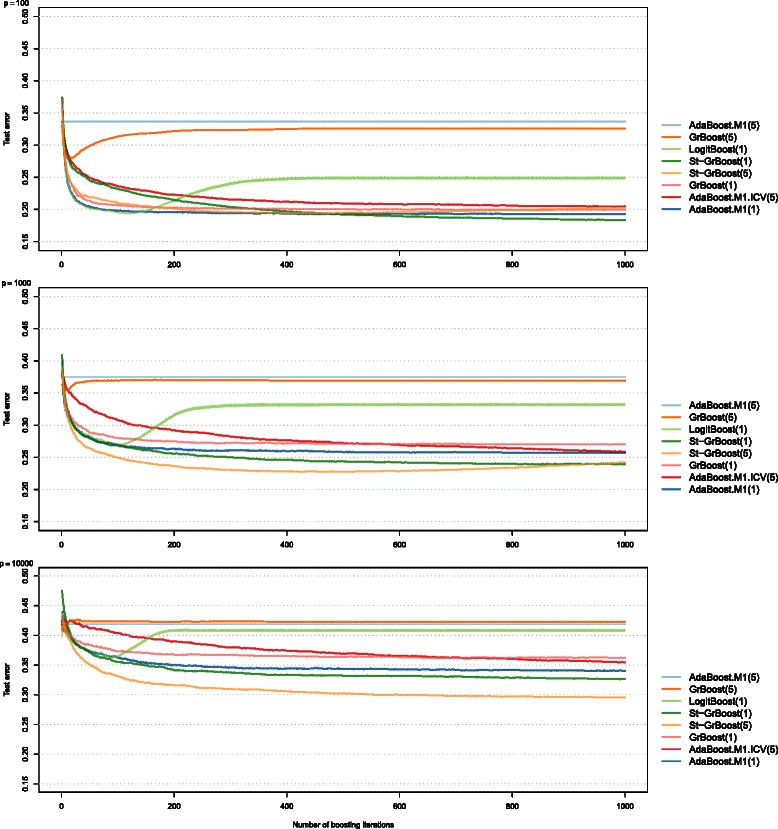
Test-set error for different classifiers, number of variables and boosting iterations. The figure reports the average test set error as a function of the number of boosting iterations for different number of variables (*p*=100, 1000 and 10000). The difference between the classes was moderate, the correlation structure was exchangeable and there were 10 variables per block and 100 differentially expressed variables, see the “Methods” section for more details

Here we report a selected series of simulation results used to illustrate the performance of various boosting algorithms when increasing the dimensionality of the feature space (Fig. 3). Figure 3 presents the test set error as a function of the number of boosting iterations. The panels refer to different number of variables and different lines represent the boosting techniques.

Boosting algorithms generally improved the performance of their base classifier; in line with the results presented in Section “Performance of AdaBoost.M1 in the high-dimensional setting”, AdaBoost.M1(5) performed as it base classified, while AdaBoost.M1(1) achieved smaller test set errors.

LogitBoost was very prone to overfitting: its test set error initially decreased with the number of boosting iterations, but around 100 iteration it increased rapidly before stabilizing again at around 200–300 iterations. Some overfitting occurred also for GrBoost(5), which was more obvious with less variables; with many variables GrBoost(5) behaved similarly to AdaBoost.M1(5).

For the other algorithms, combining more classifiers (increasing the number of boosting iterations) resulted in smaller test set errors, but increasing the number of boosting iterations beyond 200 or 300 had only a marginal effect on the test set error. The most prominent exception was AdaBoost.M1.ICV, where more iterations were needed to achieve the smallest test set error when compared with the other classifiers.

Overall, in these settings the best performance was obtained with St-GrBoost(1) (small number of variables) and St-GrBoost(5) (large number of variables).

The test set error as a function of the number of boosting iterations decreased more when there were less variables. When we adjusted the differences between the classes so as to achieve approximately the same test set errors using CART(5), regardless of the number of variables, we observed that the gain from boosting was approximately the same in all the settings (Additional file 4). Therefore, the differences could be attributed to the different power for correctly identifying the variables that were differentially expressed between the classes.

### Effect of the sample size, number of variables and between class difference

**Fig. 4:**
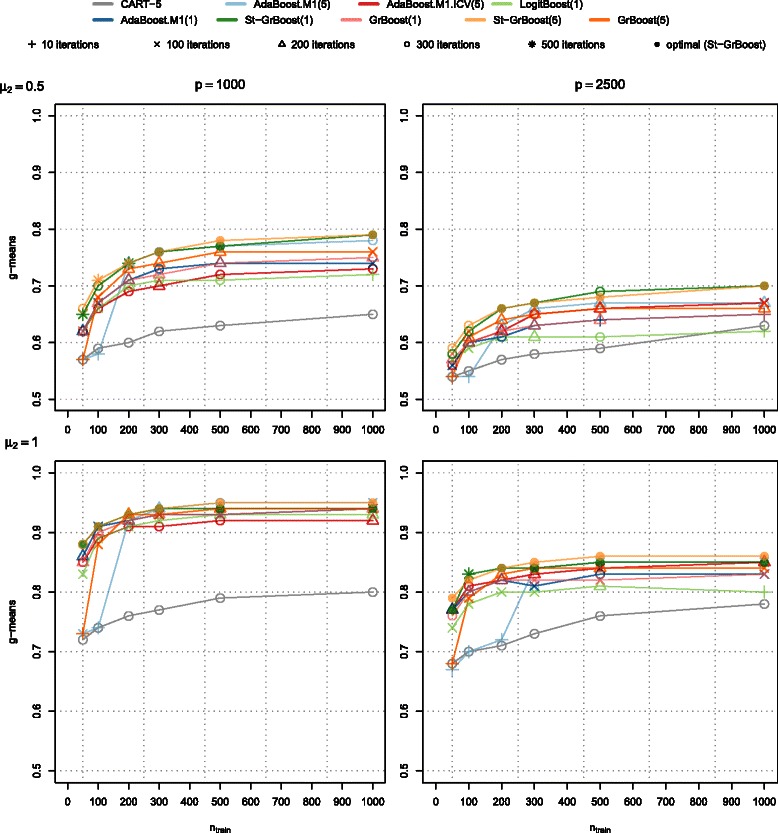
Effect of the difference between the classes, the size of the training set and the number of variables on boosting algorithms. The figure shows g-means for different size of the training set (*n*_*train*_), difference between the classes (upper panels: *μ*_2_=0.5, lower panels: *μ*_2_=1 for 100 differentially expressed variables) and the number of variables (left panels: 1000 variables, right panels: 2500 variables) for the (minimum) number of boosting iterations that produced the best classification results in terms of g-means. The training set was balanced and contained 1000 variables. Different symbols denote different number of boosting iterations, while different colors denote different ensembles

In this section we investigate how the algorithms perform when the size of the training set is increased. We also varied the difference between the classes (*μ*_2_) and the number of variables. The results are shown in Fig. 4 (left panels: 1000 variables, right panels: 2500 variables, upper panels: *μ*_2_=0.5, lower panels: *μ*_2_=1); exact numerical results as well as the results for the other performance measures are shown in Additional file 5. Different symbols denote the number of boosting iterations where the best classification result in terms of the accuracy measure was obtained.

All the algorithms performed better with bigger training sets, when the difference between classes was larger and when there were less variables that were not differentially expressed between the classes (null variables). The ability of AdaBoost.M1(5) to perform better than its base classifier depended on the sample size (generally AdaBoost.M1(5) performed the same as its base classifier with sample size smaller than 200 or 300), the number of variables (when more variables were considered, more samples were needed to see some improvement over CART(5)) and the difference between the classes (when the difference was larger more samples were needed to see substantial improvement). AdaBoost.M1.ICV(5) outperformed AdaBoost.M1(5) in the settings where the former did not perform better than its base classifier. It also performed well when there were more variables.

Similarly as AdaBoost.M1(5), GrBoost(5) was unable to improve the performance of its base classifier when the sample size was small (50 samples), but its performance improved with larger sample sizes. The performance of Gradient boosting with stumps was better than with CART(5) when there were less than 100 samples, while the results were similar for larger sample sizes. Stochastic Gradient boosting performed much better than Gradient boosting in all the settings. The performance of St-GrBoost with CART(5) and stumps was very similar (Additional file 5), the observed differences could be ascribed to simulation variability. LogitBoost in these settings performed poorly in comparison with the other classifiers.

Increasing the number of boosting iterations beyond 100 was beneficial for AdaBoost.M1.ICV(5), St-GrBoost and AdaBoost.M1(5) (limited to situations with a sufficiently large sample size, i.e. situations where AdaBoost.M1(5) was able to improve the performance of its base classifier), it only marginally improved the performance of AdaBoost.M1(1) and GrBoost(5), while it did not yield any improvement for GrBoost(1) and LogitBoost (especially when there were many variables and the size of the training sets was large). Generally, the optimal number of boosting iterations was smaller when there were more null variables; see also Additional file 4. Selecting the optimal number of boosting iterations based on the out-of-bag estimate for Stochastic Gradient boosting worked well for both stumps and CART(5) with only few exceptions.

The classifiers were also ranked by their respective geometric mean of class-specific PAs (g-means, Table 5) and the performance of AdaBoost.M1.ICV(5) was statistically compared with the performance of other classifiers. The best overall performance was obtained with St-GrBoost(5) and St-GrBoost(1); the performance of these two classifiers was significantly better than the performance of AdaBoost.M1.ICV(5). AdaBoost.M1.ICV(5) performed significantly better than CART(5) and LogitBoost, while the overall performance of AdaBoost.M1.ICV(5) was not significantly different than the performance of other classifiers.

**Table 5:** Ranking of the classifiers for different simulation settings. Ranking of the classifiers for different simulation settings; the highest g-means obtained with different number boosting iterations was considered when ranking the classifiers

The statistical comparison between AdaBoost.M1.ICV(5) and the other classifiers was performed with the Wilcoxon signed-rank test comparing their g-means; Holm’s method was used to adjust the p-values for multiple comparisons. Darker shading denotes better classifier’s performance in terms of g-means

^†^ Adjusted p-value<0.05

1 = CART(5), 2 = AdaBoost.M1(5), 3 = AdaBoost.M1.ICV(5), 4 = LogitBoost(1), 5 = AdaBoost.M1(1), 6 = St-GrBoost(1), 7 = GrBoost(1), 8 = St-GrBoost(5), 9 = GrBoost(5)

### Simulation setup with a complex separation between the classes

The simulation design used so far favored AdaBoost.M1 with stumps as base classifiers, as there were no interaction effects between the differentially expressed variables and the classes were linearly separated. We performed a limited set of simulations where the separation between the classes was more complex.

**Fig. 5:**
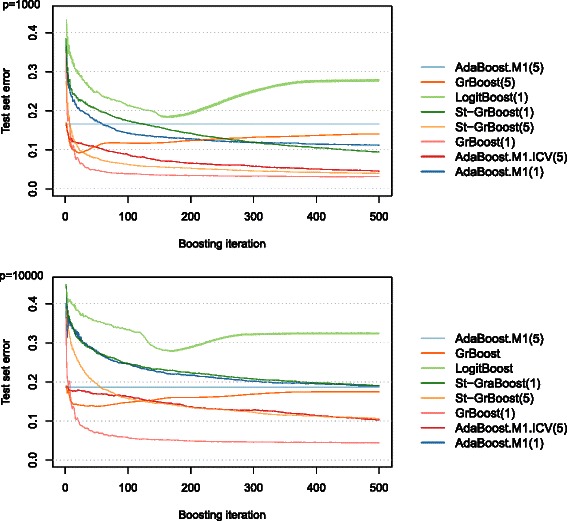
Test-set error for different classifiers and number of variables in the simulation setting with complex separation between the classes. The figure reports the average test set error for the simulation setup with the complex separation between the classes for different number of boosting iterations (from 1 to 500); upper panels: 1000 variables, lower panels: 10000 variables. See text for more details

In these settings AdaBoost.M1.ICV achieved much smaller test set error than AdaBoost.M1(1) and in general boosting algorithms using CART(5) outperformed the algorithms using stumps (Fig. 5). The exception was Gradient boosting where much better classification results were obtained with stumps. The main reason for this was that the sample size was in this setting small, hence GrBoost(5) performed similarly as its base classifier (this is consistent with the results presented so far). With larger sample sizes (*n*_*train*_≥200), GrBoost(5) outperformed GrBoost(1) (data not shown). LogitBoost performed poorly in this setting and achieved substantially higher test set error than the other classifiers.

### Determining the number of boosting iterations with cross-validation

In this section we present the results where we used cross-validation to determine the number of boosting iterations using the same settings as in Table 3. Results are summarized in Table 6.

**Table 6:** Test set error obtained with optimal number of boosting iterations (optimal), after performing 1000 boosting iterations (*M*=1000), the number of boosting iterations determined with 5-fold cross-validation (CV), leave-one-out cross-validation (LOOCV) and the number of boosting iteration determined by using out-of-bag samples (OOB)

	Optimal	*M*=1000	5-fold CV	LOOCV	OOB
GrBoost(1)	0.26 (336)	0.27	0.30 (108)	0.32 (86)	
GrBoost(5)	0.33 (6)	0.37	0.37 (10)	0.36 (16)	
St-GrBoost(1)	0.23 (594)	0.24	0.27 (224)	0.25 (425)	0.26 (286)
St-GrBoost(5)	0.22 (477)	0.24	0.26 (189)	0.24 (323)	0.24 (502)
LogitBoost	0.26 (85)	0.33	0.30 (48)	0.31 (38)	
AdaBoost.M1(1)	0.24 (343)	0.26	0.30 (155)	0.35 (75)	
AdaBoost.M1.ICV(5)	0.25 (729)	0.26	0.30 (337)	0.31 (333)	

The numbers in the brackets are the number of boosting iterations averaged over 100 simulation runs

With the exception of GrBoost(5) (which in this setting was unable to improve the performance of its base classifier), and LogitBoost (that suffered from overfitting), it was better to use 1000 boosting iterations instead of selecting its number by cross-validation; the former approach yielded smaller test set errors, which were only slightly larger than the optimal test set errors.

The cross-validated number of iterations was generally much smaller than the optimal number of iterations; consequently, the test set errors were larger than the optimal (about 0.05 larger for 5-fold CV). Overall, LOOCV and 5-fold CV performed similarly. The best performance was obtained with LOOCV for Stochastic Gradient Boosting and with 5-fold CV for the other algorithms; however, the differences were large (>0.05) only for AdaBoost.M1(1).

### Summary of the main results

- AdaBoost.M1(5) performed similarly as its base classifier when the number of samples was small (*n*_*train*_≤200, depending on the number of variables and the magnitude of the between class difference). A similar behavior was observed also for GrBoost(5), but this was limited to situations with a very small sample size (*n*_*train*_=50).
- AdaBoost.M1.ICV(5) outperformed AdaBoost.M1(5) in the setting with small sample size and performed similarly otherwise. The method performed similarly as AdaBoost.M1(1). We showed that AdaBoost.M1.ICV(5) can outperform AdaBoost.M1(1) using an example where the separation between the classes was complex.
- Shrinkage did not improve the performance of Gradient boosting, the only exception was the setting with a large sample size (500 samples) when using stumps as base classifiers. Shrinkage improved the performance of Stochastic Gradient boosting, which could in some settings (large number of variables and small sample size) overfit when shrinkage was not used. However, the performance of Stochastic Gradient boosting was good and in general it was better than the performance of the other boosting algorithms.
- LogitBoost was likely to overfit when performing a large number of boosting iterations. However, even when using a small number of boosting iteration the performance of LogitBoost was poor when compared with the other methods, especially when the number of variables and the training sample size were large.
- Using cross-validation to determine the number of boosting iterations underestimated the optimal number of iterations needed to obtain the smallest test set error and the classification results were therefore suboptimal. With the exception of LogitBoost and GrBoost(5) it was better to use a fixed large number of boosting iterations instead of cross-validation.

### Analysis of real datasets

The boosting algorithms were used to reanalyze three breast cancer microarray gene expression data.

We ranked the classifiers by their g-means, considering the highest g-means obtained with different number of boosting iterations (Table 7). Overall, the best performance was obtained with St-GrBoost(5) (median rank 1), closely followed by St-GrBoost(1) (median rank 2). The overall performance of the other classifiers was similar, with the exception of CART(5) and AdaBoost.M1(5) that performed poorly when compared with the other classifiers.

**Table 7:** Ranking of the classifiers for real breast cancer microarray datasets. The table reports the ranking of the classifiers when considering the highest g-means obtained with different number boosting iterations for different datasets/classification tasks

With n we denote the number of samples used in the analysis after randomly down-sizing the majority class; g-means is the averaged cross-validated g-means of CART(5). Darker shading denotes better classifier’s performance in terms of its g-means

1 = CART(5), 2 = AdaBoost.M1(5), 3 = AdaBoost.M1.ICV(5), 4 = AdaBoost.M1(1), 5 = LogitBoost(1), 6 = St-GrBoost(1), 7 = GrBoost(1), 8 = St-GrBoost(5), 9 = GrBoost(5)

AdaBoost.M1(5) achieved better classification results than its base classifier only for the Wang dataset (for the prediction of Relapse), which was expected as this was the largest dataset and the prediction task was hard (Additional file 6, Table 7). For the other tasks it performed worse than AdaBoost.M1.ICV and AdaBoost.M1(1). GrBoost(5) also performed as its base classifier for the Sotiriou dataset (prediction of ER but not Grade), which is in line with our simulation results where we showed that GrBoost(5) does not perform better than its base classifier when the sample size is small and/or when the difference between the classes is larger (note that the ER prediction is a much easier prediction task than the prediction of Grade). The best results were obtained with Stochastic Gradient boosting. This is in line with our simulation results where we observed that the algorithm performs well when learning from few data. In general, the performance of Stochastic Gradient boosting obtained when using CART(5) and stumps as base classifiers was very similar and did not differ by more than 0.01 for all datasets/classification tasks.

## Discussion

In this paper we evaluated the performance of various boosting algorithms for two-class prediction problems with high-dimensional data, presenting a series of simulation studies and re-analyses of real high-dimensional data sets. We proposed a novel version of the AdaBoost.M1 algorithm, which can be useful for high-dimensional data.

AdaBoost.M1 was previously reported to perform poorly with high-dimensional data [22, 23]; our results show that when the number of variables is much larger than the number of samples the poor performance of AdaBoost.M1 can be often explained by the fact that even apparently weak base classifiers can overfit the data and achieve a perfect prediction of the training set samples (i.e., a zero resubstitution error). Often the samples from a small training set can be accurately separated by a weak classifier, which is based on the combination of few variables selected among the thousands being measured; while this is seldom the case for low-dimensional data, for high-dimensional data this happens often even when the true differences between the classes are small or non existent.

The practical consequence of this is that there is no advantage in using boosting instead of its base classifier: the weights in AdaBoost.M1 are not updated and all the subsequent base classifiers produce the same prediction results. The use of weaker base classifiers can diminish this problem. For example, on real microarray data, comprising about a hundred samples and thousands of variables, we observed that generally AdaBoost.M1 performs better if the base classifier is a classification tree with with only one split (stump) rather than a larger tree (CART(5) in our analyses). Our simulation results with thousand variables indicate that base classifiers stronger than stumps can be beneficial, not overfitting the training data, only if more than hundred samples from each class are included in the training set as in this case the overfitting of the base classifier is smaller.

To overcome these problems we propose AdaBoost.M1.ICV, a modification of the AdaBoost.M1 algorithm that uses the cross-validated error rate for the update of the weights in the boosting algorithm. We show that in the high-dimensional setting with small samples AdaBoost.M1.ICV can outperform AdaBoost.M1 with larger trees as base classifiers. With AdaBoost.M1.ICV it is feasible to use base classifiers that would otherwise overfit the high-dimensional training data and impair the performance of boosting. This can be beneficial for the prediction problems that cannot be accurately addressed using very weak classifiers, as it was illustrated using data where the separation between classes was more complex.

Gradient boosting suffers from problems that are similar to those outlined for AdaBoost.M1: they are less severe as they arise for smaller sample sizes, larger number of variables, bigger between classes differences. The reason why Gradient boosting performs poorly with small samples is that the class probabilities are in the high-dimensional setting severely overfitted, similarly as the resubstitution error used by AdaBoost.M1. As a consequence the decrease of the loss function is marginal and the updates to the final score are negligible. Similarly as observed for AdaBoost.M1, this problem can be diminished by using weaker base classifiers (stumps) as in this case the overfitting of the class probabilities for the training data is less severe. The prediction results of gradient boosting depend on the choice of the value of the shrinking parameter. In the low-dimensional setting shrinkage improves the performance of Gradient boosting [32] and it is suggested that the best strategy for low-dimensional data is to set the shrinkage parameter to a very small value and then choose the number of boosting iterations by early stopping [20]. Our results show that Gradient boosting with high-dimensional data generally performs better without shrinkage.

Stochastic Gradient boosting performed better than Gradient boosting and overall, it achieved the best performance among the algorithms that were considered. The reason for its better performance can be explained by the mechanism used to update the score in the gradient boosting algorithm: only a fraction of samples is used to train the base classifier, but the score update is based on all the samples. Therefore, the update depends partly on the data used to train the classifier and partly on the left out samples. In a way this strategy is similar to the AdaBoost.M1.ICV, where cross-validated estimates were used for the updates. It was observed that Stochastic Gradient boosting performs poorly without shrinkage on low-dimensional data [20]. We observed a similar problem also for high-dimensional data, where shrinkage prevents overfitting of Stochastic Gradient boosting; the classifier overfits if the amount of shrinkage is too small, especially when there are many variables, however using too much shrinkage generally worsens the performance of the classifier. Our experimental results show that Stochastic Gradient boosting with *ν*=0.01 works well in most settings.

For low-dimensional data boosting was shown to be very robust to overfitting [46]. Our results with high-dimensional data show that LogitBoost overfits when the number of boosting iterations is large; generally the minimum test set error rate is achieved using less than 100 boosting iterations, performing more iterations leads to a substantial increase of the test set error. A similar behavior was observed also in the low-dimensional setting [47].

The other algorithms do not suffer from overfitting even after performing 1000 iterations; importantly, performing more than few hundreds boosting iterations generally only marginally affects the test set error. AdaBoost.M1.ICV achieves the minimum test set error more slowly than the other algorithms, however after many boosting iterations the ICV approach performs similarly to best performing algorithms.

The number of boosting iterations needed to achieve the best predictive performance varies substantially across different simulation settings, which suggests that the optimal number of boosting iterations should be estimated from the data for all the boosting algorithms. We addressed this issue by using cross-validation to determine the optimal number of boosting iterations. Our results indicate that this approach underestimates the optimal number of boosting iterations when the size of the dataset is small and, as a consequence, the classification performance of the algorithms is suboptimal.

The reason why this happens with small size of the training set is that the same cross-validated error rate is obtained for many different number of boosting iterations. In other words, the relatively continuous test set error function is estimated by the the cross-validated error, which is a step function (with minimal steps of 1/*n*_*train*_). We selected the smallest number of iterations in case of ties and therefore, the optimization based on a step function underestimated the optimum. In practice the problem is less important for larger sample sizes (data not shown) as in this case the function of the cross-validated error becomes more continuous. Other approaches for the determination of the optimal number of boosting iterations depend mostly on some likelihood based information criteria (for example, AIC or BIC [48, 49]). In our opinion, this approach is problematic for high-dimensional data as the class probabilities, and therefore also the likelihood, suffer from severe overfitting, even in the low-dimensional setting [50]. This suggests that the approaches based on likelihood criteria will tend to severely underestimate the optimal number of boosting iterations.

Mease and Wyner [51] showed that, for low-dimensional data, boosting with larger trees outperforms boosting with smaller trees. They argue that the reason why boosting is more efficient with larger trees is that they are less prone to overfitting than boosting with smaller trees, and provide some simulation results to support their argument. Our results for Stochastic Gradient boosting to some extent disagree with the explanation of Mease and Wyner as we observed that, using the same small amount of shrinkage, St-GrBoost(5) can overfit in some settings while St-GrBoost(1) does not.

Others argued that larger trees can capture higher-level interaction effects among the variables, while stumps can only capture the main effects but perform best with boosting methods when the generative model is additive [20].

Our limited exploration of the effect of the size of the trees on boosting performance showed that the overfitting of the base classifier hinders the performance of boosting and should be avoided or controlled (as discussed previously). Smaller trees are less prone to overfitting and are therefore preferable for AdaBoost.M1 and Gradient Boosting, unless the data are generated by a very complex model that cannot be accurately captured by combining simple base classifiers. For Stochastic Gradient boosting, which embeds some control for the overfitting of the base classifier, stumps and larger trees performed similarly when a large number of boosting iterations was performed: larger trees performed better with fewer iterations. This can easily be explained by noting that in our simulation study the differences between the classes were additive, i.e. we only considered main effects in our simulation study. Because of this, the differences between the classes could have been equally well explained by combining many classifiers where each considered only one variable or combining fewer classifiers where each considered more variables. However, we showed that also Stochastic Boosting can benefit from larger trees when the data generating process is complex.

We did not systematically evaluate the effect of the actual tree size, as we only looked at stumps and CART with settings resulting in relatively small trees. However, we can reasonably expect that AdaBoost.M1 with large trees as base classifiers will not be effective in the high-dimensional setting, unless the sample size is very large, which is uncommon in the practical applications. We constrained the trees to be of a fixed size, as proposed by [20], rather than using pruning. The reason was two-fold: trees grown without pruning are computationally more efficient, and as it was shown for the low-dimensional data that the trees obtained by pruning tend to be much too large [20]. In light of the results on the resubstitution error of CART(5) we expect that for high-dimensional data the trees obtained by pruning will tend to be even larger than for the low-dimensional data.

In the simulation study we did not perform any type of variable selection, as it is embedded in the classification trees with a predefined size. For example, stumps use only one variable that gives the best split of the training set samples in two nodes, while in our implementation CART(5) used at most 31 variables (in practice the actual number of used variables was even smaller, due to the other stopping criteria). The results for the two-class prediction tasks presented in [29] show that the reduction of the variable space does not significantly affect the results when data are class-balanced. In our reanalysis of breast cancer datasets we performed class-independent pre-filtering (1000 variables exhibiting the largest variance were considered) which was used manly for the purpose of reducing the computing time.

## Conclusions

AdaBoost.M1 with large trees does not perform better than its base classifier when data are high-dimensional. We showed that large trees achieve perfect prediction of the training set samples even when there is no true difference between the classes, therefore the weights used in AdaBoost.M1 are not updated and the boosted classifier yields exactly the same prediction result as its base classifier. A similar issue is observed also for Gradient boosting when used with few training data. One way to diminish this problem is by using weaker base classifiers, i.e. smaller trees where the extent of overfitting is smaller. If the differences between the classes cannot be accurately captured by weak base classifiers, i.e. in settings with higher level interaction effects between the variables or complex boundaries, this problem has to be accounted for in the boosting algorithm. We propose a modification of AdaBoost.M1 algorithm where we use cross-validated error rate when updating the weights. This approach performs well in our simulation study and is robust to overfitting even when the number of boosting iterations is very large. Stochastic Gradient boosting can also avoid the problems arising from overfitting of its base classifier: only a fraction of the samples is used to train the base classifier, while the score updates are based on all the samples. Overall, Stochastic Gradient boosting with the shrinkage parameter set to a small value achieved the best classification performance in our simulation study as well as using real high-dimensional microarray data.

## Additional files

Additional file 1
**Detailed description of the classifiers.** In the Additional file we provide a description of each classifier used in the paper. (PDF 201 kb)

Additional file 2
**Resubstitution error rate of CART and decision stumps (1 table).** In the Additional file we report the mean and maximum resubstitution error rate for different number of variables (*p*) and number of samples (*n*_*train*_) in the setting where there is no difference between the classes (the true error rate in this setting is 0.5). (PDF 38 kb)

Additional file 3
**Test set error as a function of the number of boosting iterations for different gradient boosting algorithms (4 figures).** In the Additional file we present the average test set error obtained with different gradient boosting algorithms where we varied the size of the training set (*n*_*train*_, rows), number of variables (*p*, columns) and the shrinkage parameter (*ν*). (PDF 2386 kb)

Additional file 4
**Over-fitting in the high-dimensional setting (1 figure).** The figure in the Additional file reports the average test set error for the example presented in the main text for different number of variables (*p*=100, 1000 and 10000) and boosting iterations (from 1 to 1000), where the difference between the differentially expressed variables increased with increasing number of variables, assuring that a single CART had roughly the same predictive power in all settings (*μ*_2_=0.7, 0.8, 1, for *p*=100, 1000 and 10000, respectively). See text for more details. (PDF 384 kb)

Additional file 5
**Simulation results obtained on simulated data (4 tables).** In the Additional file we report the accuracy measures (predictive accuracy - PA, predictive accuracy for class 1 - PA_1_, predictive accuracy for class 2 - PA_2_, g-means and AUC) obtained by training various boosting algorithms on class-balanced data (*k*_1_=0.5) when changing the size of the training set (*n*_*train*_). Results are reported for the situation where the difference was small (*μ*_2_=0.5) or moderate (*μ*_2_=1); there were 1000 or 2500 variables (*p*) for each sample in the training set containing *n*_*train*_ samples. (PDF 142 kb)

Additional file 6
**Results obtained by reanalyzing real microarray datasets (1 table).** The table in the Additional file reports the performance measures (predictive accuracy - PA, predictive accuracy for class 1 - PA_1_, predictive accuracy for class 2 - PA_2_, g-means and AUC) for different ensemble classifiers and datasets/prediction tasks; darker shading denotes better performance in terms of the relevant accuracy measure. (PDF 111 kb)

## Abbreviations

ICV: Internal cross-validation
CART: Classification and regression trees
PA: Predictive accuracy
AUC: Area under the ROC curve
CV: Cross validation
GrBoost: Gradient boosting
St-GrBoost: Stochastic Gradient boosting
