# Supplementary material for: Boosting for high-dimensional two-class prediction
Source: BMC Bioinformatics. 2015 Sep 21;16:300. doi: 10.1186/s12859-015-0723-9 (PMC4578758; doi:10.1186/s12859-015-0723-9)
Supplement: Additional file 1 — Detailed description of the classifiers. In the Additional file we provide a description of each classifier used in the paper. (PDF 201 kb) [file 12859_2015_723_MOESM1_ESM.pdf]

Description of the classifiers

Notation

With x_{ij} we denote the value of variable j ($j = 1, ..., p$) for sample i ($i = 1, ..., n$); $\mathbf{x}_i$ is the set of variables for sample i and $\mathbf{x}$ is the set of variables for all samples. Some of the n samples are known to belong to Class 1 (n_1 samples) and the others to Class 2 (n_2, $n_1+n_2 = n$); the proportion of samples from Class 1 and 2 is denoted by $k_1 = n_1/n$ and $k_2 = n_2/n$, respectively, and $y_i = \{-1, 1\}$ is the class membership for sample i. Let $\widehat{y_i} = c(\mathbf{x}_i)$ be the predicted class for sample i.

Classifiers

CART [1] and decision stumps (classification trees with only one split, referred to as stumps throughout the paper and denoted by CART(1)) were used as base classifiers. In CART(5) we did not use pruning, however the maximum depth of any node of the final tree was set to 5 (resulting in a relatively small tree), the complexity parameter was -1, there had to be at least two samples in the node to attempt the split and the Gini index was used as an impurity measure; the tuning parameters of CART were the same for all boosting methods. Next we present a short description of each classifier used in the paper.

LogitBoost [2]. The classifier fits an additive logistic regression model by stagewise optimization of the binomial log-likelihood; more details can be found in [2]. We used function *LogitBoost* in package caTools that uses decision stumps as weak classifiers. Samples with equal probability of classification in each class were randomly assigned to one of the classes.

AdaBoost.M1(5) [3, 4]. The algorithm starts by training CART(5) (c_1) on the original training set, using weights $w_1(i) = \frac{1}{n}$, for $i = 1, ..., n$. For classifier c_1 the re-substitution error rate is estimated as $\epsilon_1 = \sum_{i=1}^{n} w_1(i) z_i$, where z_i is the indicator for a miss-classified sample i, i.e. $z_i = 1$ if $c_1(\mathbf{x}_i) \neq y_i$ and zero otherwise ($c_1(\mathbf{x}_i)$ is predicted class for sample i based on classifier c_1). New weights are then defined as

$$w_{t+1}(i) = \frac{w_t(i)\exp(\alpha_t z_i)}{Z_t}, \quad (1)$$

where $\alpha_t = ln(\frac{1-\epsilon_t}{\epsilon_t})$, $t = 1, ..., T$ (T is the number of boosting iterations), and Z_t is a normalization factor assuring that the sum of new weights is equal to one ($\sum_{i=1}^{n} w_{t+1}(i) = 1$). The final classification rule after T boosting iterations is then given by

$$\mathcal{C}(\mathbf{x}) = \text{sign}\left(\sum_{t=1}^{T} \alpha_t c_t(\mathbf{x})\right). \quad (2)$$

Note that when $\epsilon_t = 0$, the updated weights are the same as the original weights ($w_{t+1}(i) = w_t$, for $i = 1, ..., n$) and therefore $c_{t+1} = c_t$ for all subsequent iterations.

AdaBoost.M1(1). Decision stumps (CART(1)) with Gini index as impurity measure were used as a weak classifier in AdaBoost.M1.

GrBoost(5) [5]. We used function *gbm.fit* to fit the model. CART(5) with the same settings as described for AdaBoost.M1(5) was used as a base classifier. Unless not stated otherwise, the shrinkage parameter was set to 1 (we performed a limited set of analyses with other values of the shrinkage parameter but generally obtained worse results) and exponential loss was minimized. Since the output of the classifier is a probability for classification to class k ($\widehat{p}_k$), the final class assignment was defined as, classify to class 1 if $\widehat{p}_1 > 0.5$, to class 2 if $\widehat{p}_1 < 0.5$ and classify at random otherwise. More details on the method can be found in [6].

GrBoost(1) [5]. Stump was used as a base classifiers in Gradient Boosting. The other settings were the same as for GrBoost(5).

St-GrBoost(5) [7]. Instead of using the entire training set to fit the model in each boosting iterations 50% of the samples were randomly selected and included to fit the model. Function *gbm.fit* from the gbm package was used to train the classifier and function *gbm.perf* was used to determine the optimal number of boosting iterations based on the out-of-bag estimate. Shrinkage parameter was set to 0.01 unless not stated otherwise (we performed a limited set of simulations with other values of the shrinkage parameter but generally obtained worse results). CART(5) was used as a base classifier. More details can be found in [6].

St-GrBoost(1) [7]. Stump were used as a base classifier in Stochastic Gradient Boosting, the other settings were the same as described for St-GrBoost.

AdaBoost.M1.ICV. Instead of using the re-substitution error rate when updating the weights in AdaBoost.M1 algorithm we used cross-validated error rate. The training set in each boosting iteration was split into k subsets (the size and the level of class-imbalance in each subset was approximately the same) and then each of the k subsets was left out wile the rest of the subsets were used for training classifier c_t, using weights $w_t(i)$. The classifier was then used to predict the class for the left out samples ($\widehat{y_i}^{\mathrm{CV}}$). The cross-validated error rate was then calculated as $\epsilon_t^{\mathrm{CV}} = \sum_{i=1}^{n} w_t(i) z_i^{\mathrm{CV}}$, where $z_i^{\mathrm{CV}} = 1$ if $\widehat{y_i}^{\mathrm{CV}} \neq y_i$ and zero otherwise. z_i^{CV} and ϵ_t^{CV} were then used to update the weights and to calculate α_t; the final classification rule was then the same as in AdaBoost.M1.

Algorithm 1 AdaBoost.M1.ICV.

1: Initialize the observation weights $w_i = \frac{1}{n}$, $i = 1, 2, ..., n$.
2: $m = 0$
repeat
3: $m = m + 1$
4: Fit a regression tree (CART(5), $c_m(\mathbf{x})$) to the training data using weights w_i.
5: Compute a cross-validated error rate of $c_m(\mathbf{x})$,

$$\epsilon_m^{\mathrm{CV}} = \sum_{i=1}^{n} w_i z_i^{\mathrm{CV}},$$

where $z_i^{\mathrm{CV}} = 1$ if $\widehat{y_i}^{\mathrm{CV}} \neq y_i$ and zero otherwise.
6: Compute $\alpha_m = \log \frac{1-\epsilon_m^{\mathrm{CV}}}{\epsilon_m^{\mathrm{CV}}}$.
7: Set $w_i \leftarrow w_i \cdot \exp(\alpha_m \cdot z_i^{\mathrm{CV}})$, $i = 1, ..., n$.
until $m = M$
6: Output

$$\mathcal{C}(\mathbf{x}) = \mathrm{sign}(\sum_{m=1}^{M} \alpha_m c_m(\mathbf{x})).$$

References

[1] Breiman L, Friedman JH, Olshen RA, Stone CJ: *Classification and Regression Trees.* Pacific Grove, California: Wadsworth & Brooks 1984.

[2] Friedman J, Hastie T, Tibshirani R: **Additive Logistic Regression: a Statistical View of Boosting**. *The Annals of Statistics* 2000, **38**(2).

[3] Freund Y, Schapire RE: **Experiments with a New Boosting Algorithm**. In *Proceedings of the Thirteenth International Conference on Machine Learning* 1996:148–156.

[4] Schapire R: **The boosting approach to machine learning: An overview**. In *MSRI Workshop on Nonlinear Estimation and Classification* 2001.

[5] Friedman JH: **Greedy Function Approximation: A Gradient Boosting Machine**. *Annals of Statistics* 2000, **29**:1189–1232.

[6] Hastie T, Tibshirani R, Friedman J: *The Elements of Statistical Learning: Data Mining, Inference, and Prediction.* New York: Springer 2003.

[7] Friedman JH: **Stochastic Gradient Boosting**. *Computational Statistics and Data Analysis* 1999, **38**:367–378.
