# Supplementary material for: Boosting for high-dimensional two-class prediction
Source: BMC Bioinformatics. 2015 Sep 21;16:300. doi: 10.1186/s12859-015-0723-9 (PMC4578758; doi:10.1186/s12859-015-0723-9)
Supplement: Additional file 2 — Resubstitution error rate of CART and decision stumps (1 table). In the Additional file we report the mean and maximum resubstitution error rate for different number of variables (p) and number of samples (n train) in the setting where there is no difference between the classes (the true error rate in this setting is 0.5). (PDF 38 kb) [file 12859_2015_723_MOESM2_ESM.pdf]

Table 1: Training error rate achieved with CART(5) and CART(1). In the table we report the mean and maximum training error rate for different number of variables ($p$) in the setting when there is no difference between the classes (the true error rate in this setting is 0.5).

| | | | Number of variables | | | | | | | | | |
|---|---|---|---|---|---|---|---|---|---|---|---|---|
| | $n_{train}$ | | 10 | 15 | 20 | 30 | 50 | 80 | 100 | 500 | 1,000 | 10,000 |
| CART(5) | 50 | mean | 0.09 | 0.06 | 0.04 | 0.03 | 0.02 | 0.01 | 0.01 | 0.00 | 0.00 | 0.00 |
| | | max | 0.28 | 0.24 | 0.2 | 0.16 | 0.12 | 0.1 | 0.08 | 0.04 | 0.02 | 0.00 |
| | 100 | mean | 0.16 | 0.12 | 0.11 | 0.09 | 0.06 | 0.05 | 0.04 | 0.01 | 0.01 | 0.00 |
| | | max | 0.34 | 0.31 | 0.3 | 0.27 | 0.28 | 0.22 | 0.2 | 0.11 | 0.1 | 0.04 |
| | 200 | mean | 0.24 | 0.21 | 0.19 | 0.17 | 0.15 | 0.13 | 0.12 | 0.07 | 0.06 | 0.03 |
| | | max | 0.41 | 0.38 | 0.38 | 0.38 | 0.32 | 0.28 | 0.31 | 0.25 | 0.21 | 0.16 |
| | 500 | mean | 0.34 | 0.32 | 0.31 | 0.29 | 0.27 | 0.25 | 0.25 | 0.2 | 0.18 | 0.13 |
| | | max | 0.45 | 0.45 | 0.42 | 0.43 | 0.42 | 0.41 | 0.41 | 0.35 | 0.34 | 0.3 |
| | 1000 | mean | 0.39 | 0.38 | 0.37 | 0.36 | 0.34 | 0.33 | 0.33 | 0.29 | 0.28 | 0.23 |
| | | max | 0.47 | 0.47 | 0.47 | 0.46 | 0.45 | 0.45 | 0.43 | 0.41 | 0.4 | 0.36 |
| CART(1) | 50 | mean | 0.34 | 0.32 | 0.31 | 0.31 | 0.29 | 0.28 | 0.28 | 0.25 | 0.23 | 0.2 |
| | | max | 0.34 | 0.32 | 0.31 | 0.31 | 0.29 | 0.28 | 0.28 | 0.25 | 0.23 | 0.2 |
| | 100 | mean | 0.39 | 0.38 | 0.37 | 0.37 | 0.36 | 0.35 | 0.35 | 0.32 | 0.31 | 0.29 |
| | | max | 0.39 | 0.38 | 0.37 | 0.37 | 0.36 | 0.35 | 0.35 | 0.32 | 0.31 | 0.29 |
| | 200 | mean | 0.42 | 0.42 | 0.41 | 0.41 | 0.4 | 0.4 | 0.4 | 0.38 | 0.37 | 0.35 |
| | | max | 0.42 | 0.42 | 0.41 | 0.41 | 0.4 | 0.4 | 0.4 | 0.38 | 0.37 | 0.35 |
| | 500 | mean | 0.46 | 0.45 | 0.45 | 0.45 | 0.44 | 0.44 | 0.44 | 0.43 | 0.43 | 0.41 |
| | | max | 0.46 | 0.45 | 0.45 | 0.45 | 0.44 | 0.44 | 0.44 | 0.43 | 0.43 | 0.41 |
| | 1000 | mean | 0.47 | 0.47 | 0.47 | 0.47 | 0.46 | 0.46 | 0.46 | 0.45 | 0.45 | 0.44 |
| | | max | 0.47 | 0.47 | 0.47 | 0.47 | 0.46 | 0.46 | 0.46 | 0.45 | 0.45 | 0.44 |
