# Supplementary material for: Boosting for high-dimensional two-class prediction
Source: BMC Bioinformatics. 2015 Sep 21;16:300. doi: 10.1186/s12859-015-0723-9 (PMC4578758; doi:10.1186/s12859-015-0723-9)

Figure 1: Average test set error obtained with GrBoost(5).

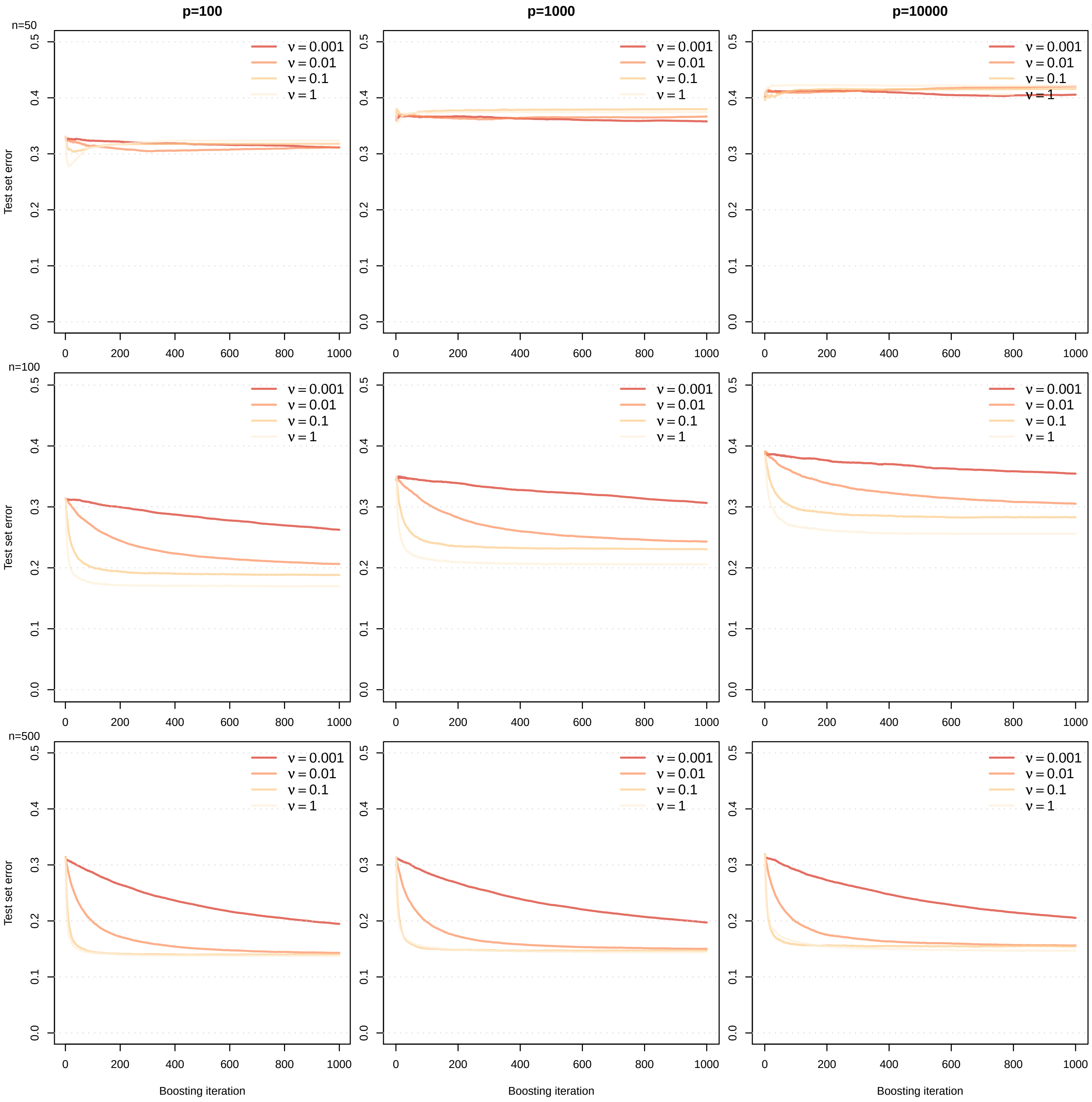

Figure 2: Average test set error obtained with GrBoost(1).

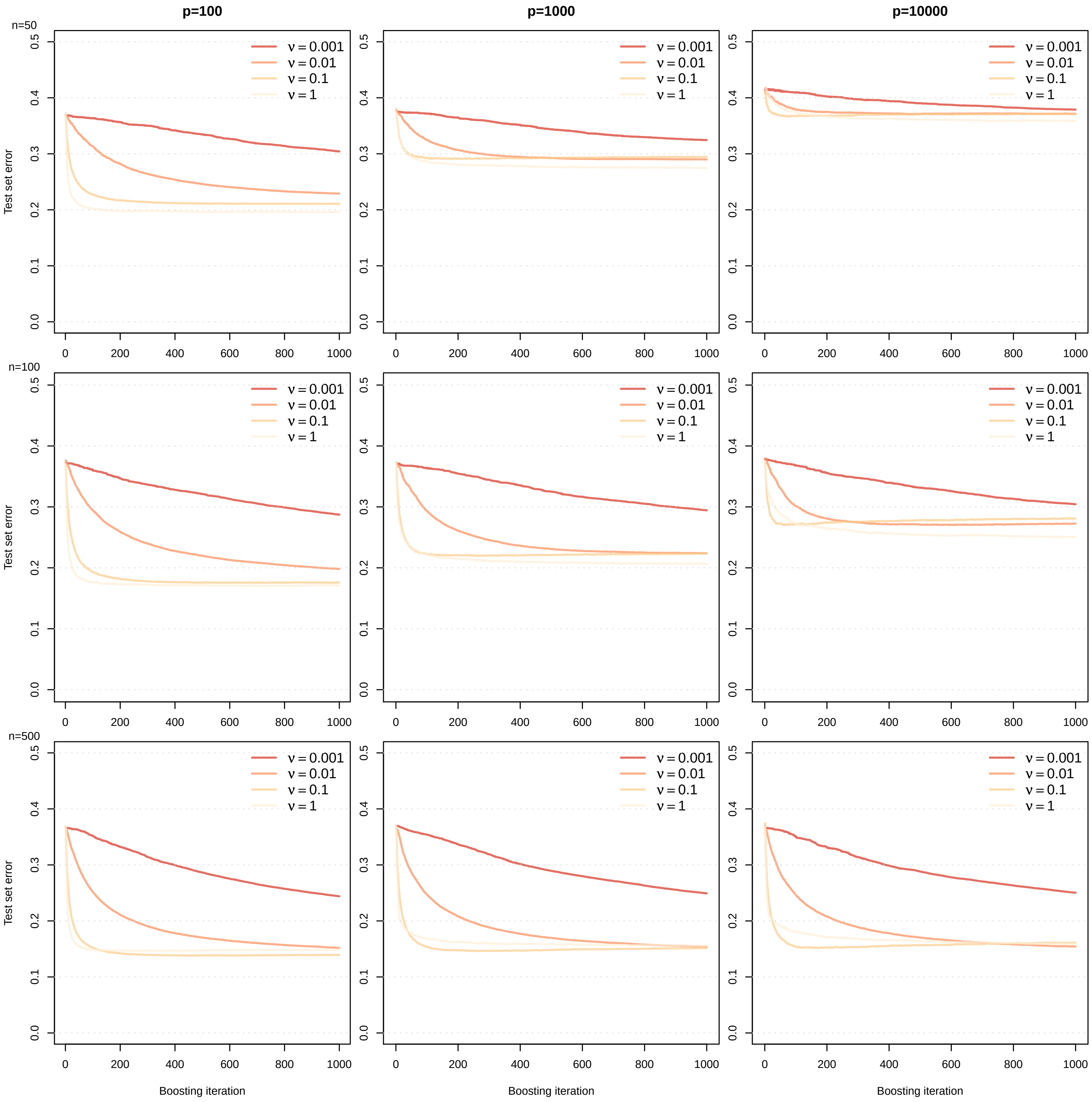

Figure 3: Average test set error obtained with St-GrBoost(5).

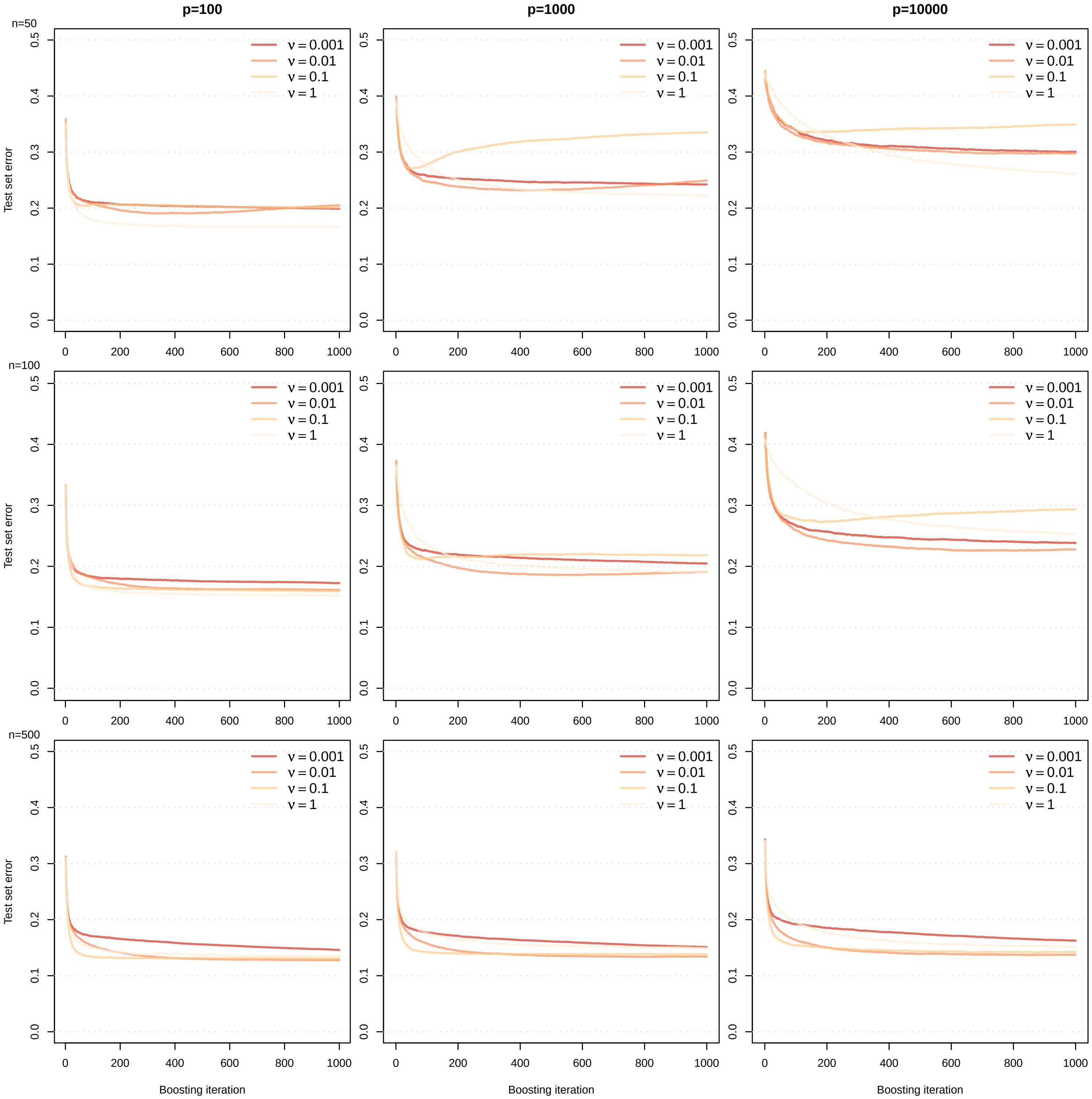

Figure 4: Average test set error obtained with St-GrBoost(1).

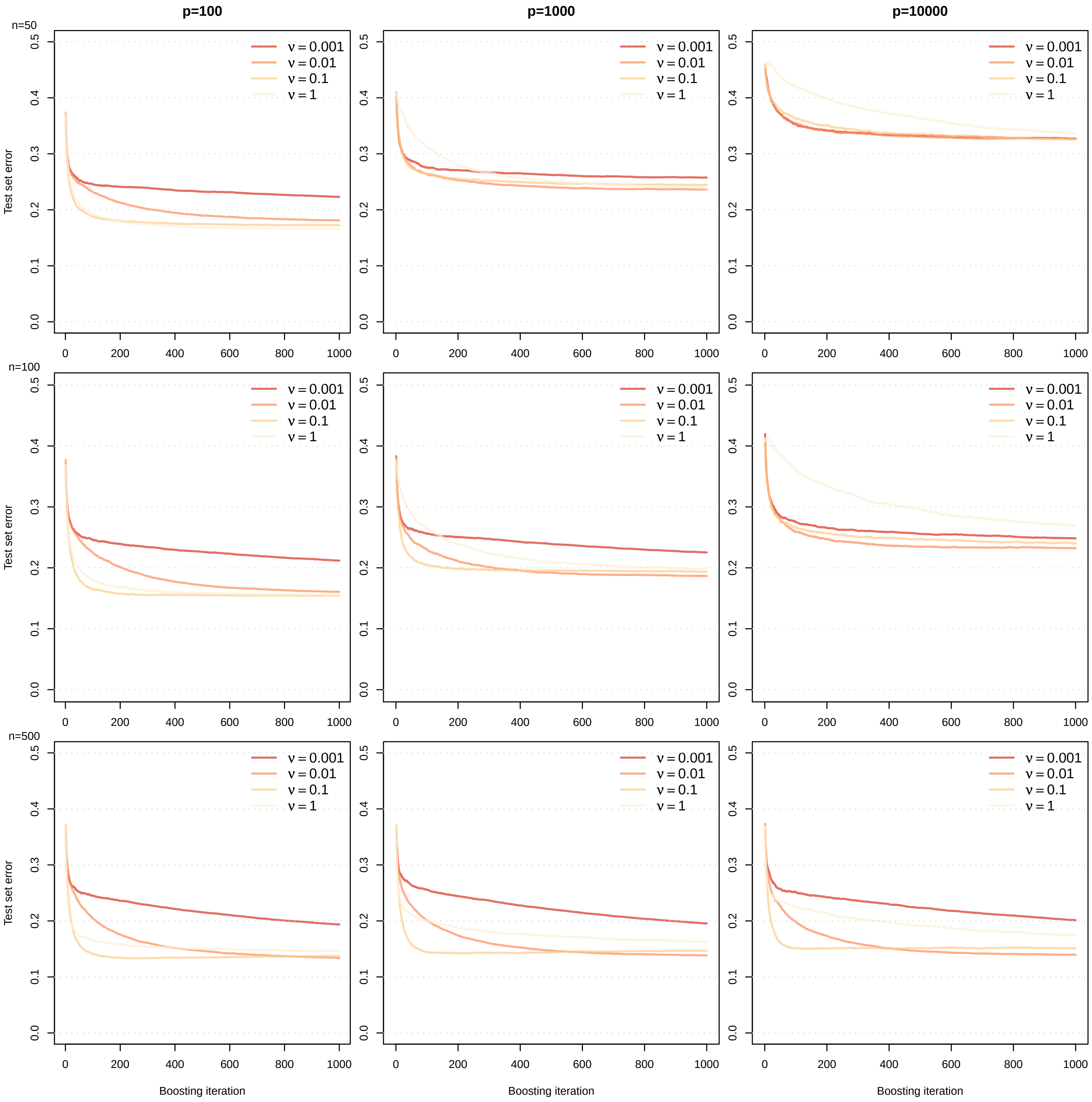

Supplement: Additional file 3 — Test set error as a function of the number of boosting iterations for different gradient boosting algorithms (4 figures). In the Additional file we present the average test set error obtained with different gradient boosting algorithms where we varied the size of the training set (n train, rows), number of variables (p, columns) and the shrinkage parameter (ν). (PDF 2386 kb) [file 12859_2015_723_MOESM3_ESM.pdf]
