# Supplementary figures and images for: Boosting for high-dimensional two-class prediction
Source: BMC Bioinformatics. 2015 Sep 21;16:300. doi: 10.1186/s12859-015-0723-9 (PMC4578758; doi:10.1186/s12859-015-0723-9)

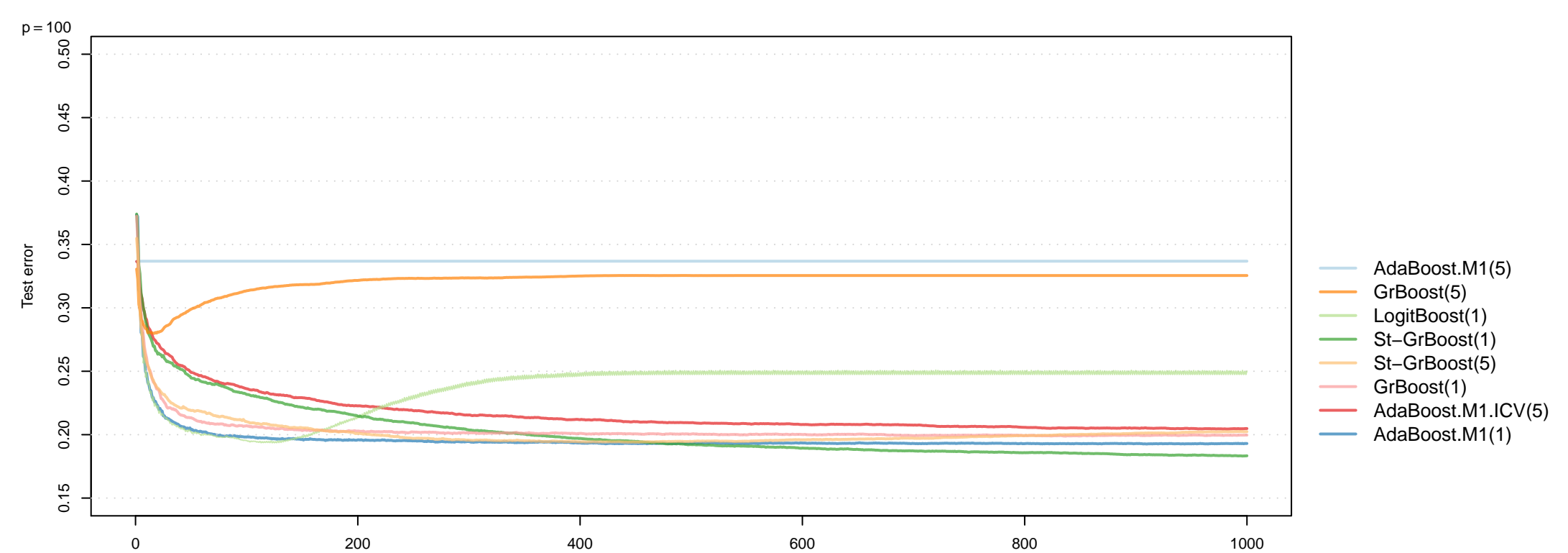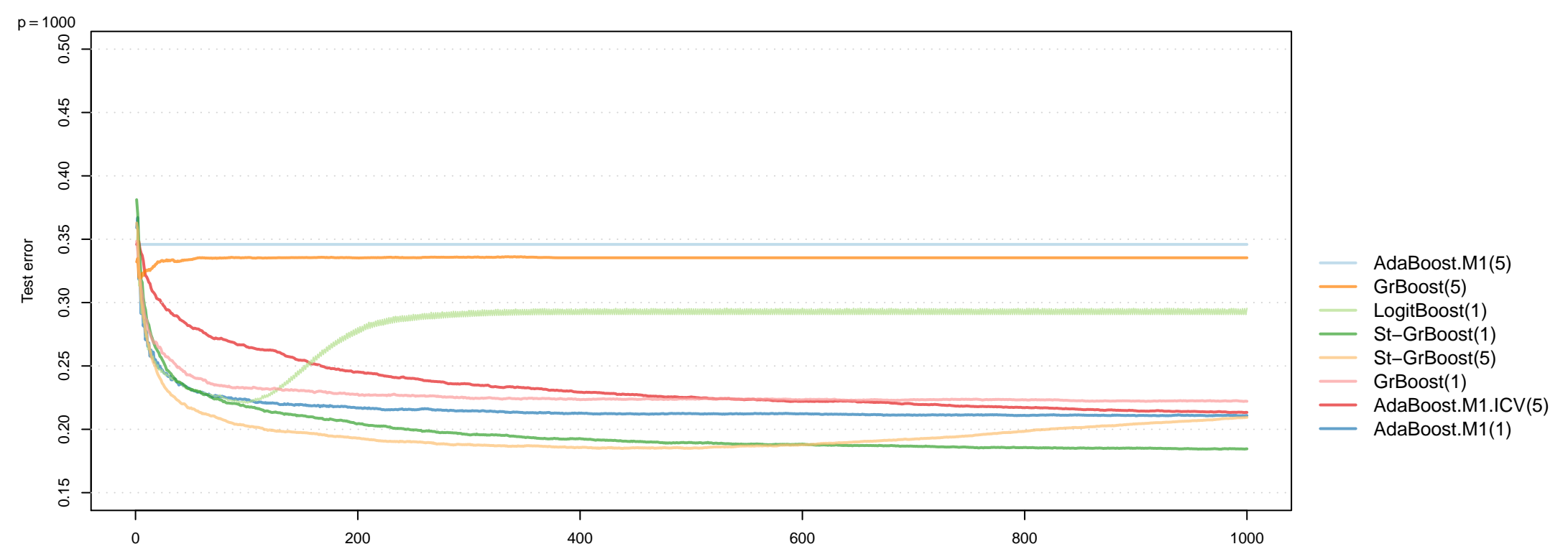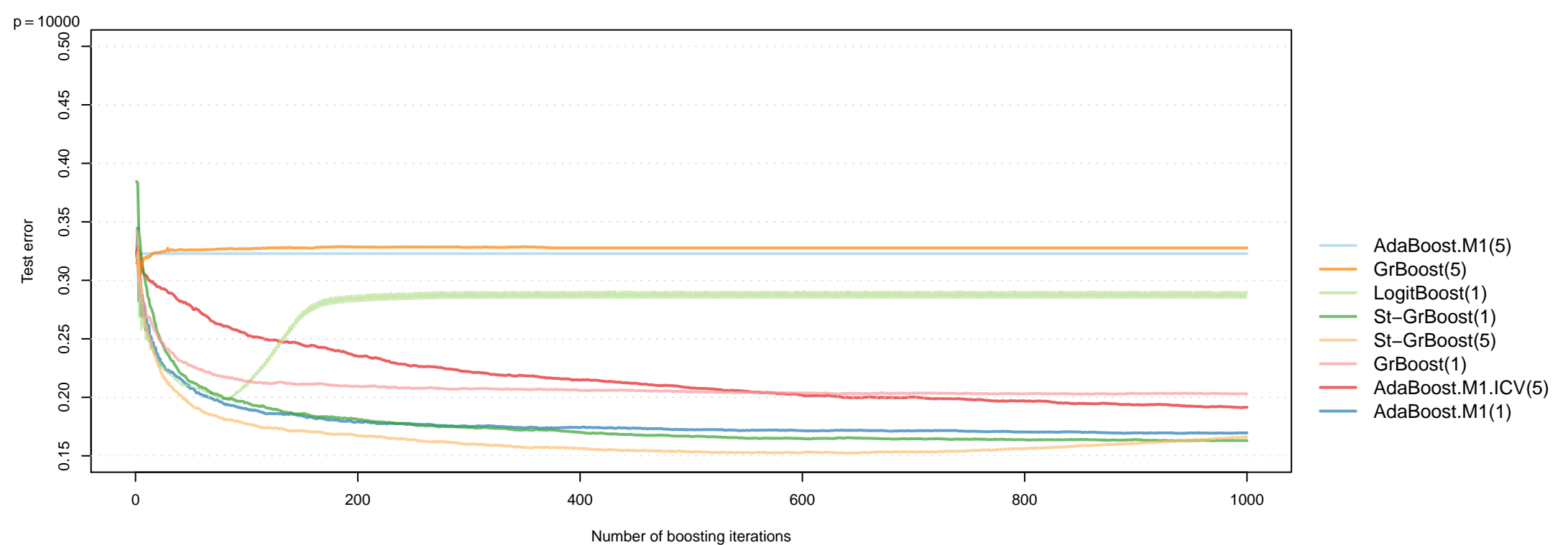

Supplement: Additional file 4 — Over-fitting in the high-dimensional setting (1 figure). The figure in the Additional file reports the average test set error for the example presented in the main text for different number of variables (p=100, 1000 and 10000) and boosting iterations (from 1 to 1000), where the difference between the differentially expressed variables increased with increasing number of variables, assuring that a single CART had roughly the same predictive power in all settings (μ 2=0.7, 0.8, 1, for p=100, 1000 and 10000, respectively). See text for more details. (PDF 384 kb) [file 12859_2015_723_MOESM4_ESM.pdf]
