# Supplementary material for: Boosting for high-dimensional two-class prediction
Source: BMC Bioinformatics. 2015 Sep 21;16:300. doi: 10.1186/s12859-015-0723-9 (PMC4578758; doi:10.1186/s12859-015-0723-9)
Supplement: Additional file 5 — Simulation results obtained on simulated data (4 tables). In the Additional file we report the accuracy measures (predictive accuracy - PA, predictive accuracy for class 1 - PA1, predictive accuracy for class 2 - PA2, g-means and AUC) obtained by training various boosting algorithms on class-balanced data (k 1=0.5) when changing the size of the training set (n train). Results are reported for the situation where the difference was small (μ 2=0.5) or moderate (μ 2=1); there were 1000 or 2500 variables (p) for each sample in the training set containing n train samples. (PDF 142 kb) [file 12859_2015_723_MOESM5_ESM.pdf]

Table 1. Performance of the classifiers for a small difference between the classes ($\mu_2 = 0.5$, $p = 1,000$). The table reports predictive accuracy (PA), predictive accuracy for class 1 and class 2 (PA_1, PA_2), g-means and AUC. Training sets contained 1,000 variables; see the main text for details.

n_{train}		CART(5)	AdaBoost.M1(5).10	AdaBoost.M1(5).100	AdaBoost.M1(5).200	AdaBoost.M1(5).300	AdaBoost.M1.ICV(5).10	AdaBoost.M1.ICV(5).100	AdaBoost.M1.ICV(5).200	AdaBoost.M1.ICV(5).300	LogitBoost(1).10	LogitBoost(1).100	LogitBoost(1).200	LogitBoost(1).300	AdaBoost.M1(1).10	AdaBoost.M1(1).100	AdaBoost.M1(1).200	AdaBoost.M1(1).300	St-GrBoost(1).opt	St-GrBoost(1).100	St-GrBoost(1).300	St-GrBoost(1).500	GrBoost(1).10	GrBoost(1).100	GrBoost(1).200	GrBoost(1).300	St-GrBoost(5).opt	St-GrBoost(5).100	St-GrBoost(5).300	St-GrBoost(5).500	GrBoost(5).10	GrBoost(5).100	GrBoost(5).200	GrBoost(5).300
50	PA	0.58	0.58	0.57	0.57	0.57	0.58	0.59	0.61	0.62	0.59	0.62	0.62	0.62	0.59	0.61	0.62	0.63	0.61	0.64	0.65	0.65	0.59	0.62	0.63	0.63	0.64	0.65	0.66	0.67	0.58	0.56	0.56	0.56
		(0.04)	(0.04)	(0.04)	(0.04)	(0.04)	(0.04)	(0.02)	(0.03)	(0.02)	(0.04)	(0.03)	(0.03)	(0.04)	(0.04)	(0.04)	(0.03)	(0.03)	(0.06)	(0.03)	(0.03)	(0.03)	(0.04)	(0.03)	(0.03)	(0.03)	(0.06)	(0.03)	(0.03)	(0.03)	(0.04)	(0.04)	(0.04)	(0.04)
	PA_1	0.59	0.58	0.57	0.57	0.57	0.57	0.58	0.61	0.6	0.59	0.61	0.61	0.61	0.6	0.6	0.63	0.63	0.63	0.65	0.66	0.67	0.59	0.61	0.61	0.61	0.64	0.64	0.65	0.66	0.57	0.56	0.56	0.56
		(0.09)	(0.08)	(0.08)	(0.08)	(0.08)	(0.08)	(0.08)	(0.06)	(0.07)	(0.07)	(0.07)	(0.07)	(0.07)	(0.08)	(0.06)	(0.07)	(0.06)	(0.09)	(0.07)	(0.07)	(0.06)	(0.08)	(0.06)	(0.06)	(0.06)	(0.09)	(0.07)	(0.07)	(0.07)	(0.08)	(0.08)	(0.08)	(0.08)
	PA_2	0.57	0.57	0.58	0.58	0.58	0.58	0.61	0.61	0.65	0.59	0.63	0.63	0.63	0.58	0.62	0.62	0.62	0.6	0.63	0.64	0.64	0.59	0.63	0.64	0.64	0.63	0.66	0.67	0.67	0.58	0.57	0.56	0.57
		(0.08)	(0.08)	(0.08)	(0.08)	(0.08)	(0.07)	(0.06)	(0.05)	(0.06)	(0.07)	(0.06)	(0.06)	(0.06)	(0.08)	(0.06)	(0.07)	(0.07)	(0.1)	(0.07)	(0.07)	(0.06)	(0.08)	(0.07)	(0.08)	(0.08)	(0.1)	(0.07)	(0.06)	(0.06)	(0.08)	(0.08)	(0.08)	(0.08)
	g-means	0.57	0.57	0.57	0.57	0.57	0.57	0.59	0.61	0.62	0.59	0.62	0.61	0.61	0.59	0.61	0.62	0.62	0.61	0.63	0.64	0.65	0.59	0.62	0.62	0.62	0.63	0.65	0.66	0.66	0.57	0.56	0.56	0.56
		(0.04)	(0.04)	(0.04)	(0.04)	(0.04)	(0.04)	(0.02)	(0.03)	(0.02)	(0.04)	(0.03)	(0.04)	(0.04)	(0.04)	(0.04)	(0.03)	(0.03)	(0.07)	(0.04)	(0.03)	(0.03)	(0.04)	(0.03)	(0.04)	(0.03)	(0.06)	(0.03)	(0.03)	(0.03)	(0.04)	(0.04)	(0.04)	(0.04)
	AUC	0.58	0.58	0.57	0.57	0.57	0.61	0.63	0.65	0.67	0.63	0.67	0.66	0.66	0.63	0.65	0.67	0.68	0.66	0.69	0.71	0.71	0.62	0.67	0.68	0.67	0.69	0.71	0.73	0.73	0.6	0.58	0.58	0.58
		(0.04)	(0.04)	(0.04)	(0.04)	(0.04)	(0.05)	(0.03)	(0.04)	(0.03)	(0.05)	(0.05)	(0.05)	(0.05)	(0.05)	(0.05)	(0.05)	(0.05)	(0.08)	(0.04)	(0.04)	(0.04)	(0.05)	(0.05)	(0.04)	(0.04)	(0.08)	(0.04)	(0.04)	(0.04)	(0.05)	(0.04)	(0.04)	(0.04)
100	PA	0.59	0.59	0.59	0.59	0.59	0.6	0.63	0.65	0.66	0.62	0.66	0.67	0.66	0.63	0.67	0.67	0.68	0.69	0.68	0.7	0.7	0.62	0.66	0.67	0.68	0.7	0.69	0.71	0.71	0.63	0.68	0.68	0.69
		(0.02)	(0.03)	(0.03)	(0.03)	(0.03)	(0.03)	(0.04)	(0.02)	(0.03)	(0.03)	(0.03)	(0.03)	(0.03)	(0.03)	(0.03)	(0.03)	(0.03)	(0.03)	(0.03)	(0.03)	(0.03)	(0.03)	(0.03)	(0.03)	(0.03)	(0.02)	(0.02)	(0.02)	(0.02)	(0.03)	(0.03)	(0.03)	(0.03)
	PA_1	0.59	0.59	0.58	0.59	0.59	0.6	0.61	0.65	0.66	0.62	0.66	0.65	0.65	0.62	0.67	0.68	0.68	0.68	0.68	0.7	0.7	0.62	0.66	0.67	0.67	0.7	0.69	0.7	0.71	0.64	0.68	0.69	0.69
		(0.05)	(0.05)	(0.05)	(0.06)	(0.06)	(0.06)	(0.07)	(0.05)	(0.06)	(0.06)	(0.05)	(0.05)	(0.05)	(0.07)	(0.03)	(0.05)	(0.05)	(0.05)	(0.05)	(0.05)	(0.05)	(0.08)	(0.05)	(0.05)	(0.05)	(0.05)	(0.05)	(0.05)	(0.05)	(0.05)	(0.04)	(0.05)	(0.04)
	PA_2	0.59	0.58	0.59	0.59	0.59	0.6	0.65	0.64	0.66	0.62	0.67	0.68	0.68	0.64	0.67	0.67	0.68	0.69	0.69	0.7	0.71	0.63	0.66	0.67	0.68	0.7	0.69	0.71	0.71	0.63	0.67	0.68	0.68
		(0.06)	(0.06)	(0.05)	(0.05)	(0.05)	(0.06)	(0.04)	(0.05)	(0.06)	(0.06)	(0.05)	(0.06)	(0.06)	(0.07)	(0.05)	(0.05)	(0.04)	(0.05)	(0.05)	(0.05)	(0.05)	(0.08)	(0.06)	(0.06)	(0.06)	(0.04)	(0.04)	(0.04)	(0.04)	(0.05)	(0.06)	(0.05)	(0.05)
	g-means	0.59	0.58	0.58	0.58	0.58	0.6	0.63	0.64	0.66	0.62	0.66	0.66	0.66	0.63	0.67	0.67	0.67	0.69	0.68	0.7	0.7	0.62	0.66	0.67	0.67	0.7	0.69	0.7	0.71	0.63	0.68	0.68	0.68
		(0.02)	(0.03)	(0.03)	(0.03)	(0.03)	(0.03)	(0.04)	(0.02)	(0.03)	(0.03)	(0.03)	(0.03)	(0.03)	(0.03)	(0.03)	(0.03)	(0.03)	(0.03)	(0.03)	(0.03)	(0.03)	(0.03)	(0.03)	(0.03)	(0.03)	(0.03)	(0.02)	(0.02)	(0.02)	(0.03)	(0.03)	(0.03)	(0.03)
	AUC	0.59	0.59	0.59	0.59	0.59	0.64	0.68	0.7	0.72	0.67	0.73	0.72	0.72	0.68	0.73	0.74	0.74	0.76	0.75	0.77	0.78	0.67	0.73	0.74	0.74	0.78	0.76	0.78	0.78	0.68	0.74	0.74	0.74
		(0.02)	(0.03)	(0.04)	(0.04)	(0.04)	(0.04)	(0.05)	(0.03)	(0.03)	(0.04)	(0.03)	(0.03)	(0.04)	(0.04)	(0.03)	(0.03)	(0.03)	(0.04)	(0.03)	(0.03)	(0.03)	(0.04)	(0.04)	(0.04)	(0.04)	(0.03)	(0.03)	(0.03)	(0.03)	(0.04)	(0.04)	(0.03)	(0.03)
200	PA	0.6	0.66	0.73	0.74	0.74	0.64	0.66	0.68	0.69	0.66	0.69	0.7	0.7	0.68	0.7	0.71	0.71	0.74	0.71	0.73	0.74	0.66	0.7	0.71	0.71	0.74	0.72	0.74	0.74	0.68	0.72	0.73	0.73
		(0.02)	(0.02)	(0.02)	(0.02)	(0.02)	(0.02)	(0.02)	(0.03)	(0.03)	(0.03)	(0.02)	(0.02)	(0.02)	(0.02)	(0.02)	(0.02)	(0.02)	(0.02)	(0.03)	(0.02)	(0.02)	(0.02)	(0.02)	(0.02)	(0.02)	(0.02)	(0.02)	(0.02)	(0.02)	(0.02)	(0.02)	(0.02)	(0.02)
	PA_1	0.6	0.65	0.73	0.74	0.74	0.63	0.64	0.66	0.67	0.65	0.68	0.69	0.69	0.68	0.69	0.71	0.71	0.74	0.7	0.73	0.74	0.67	0.7	0.71	0.71	0.75	0.73	0.74	0.75	0.68	0.72	0.73	0.73
		(0.05)	(0.04)	(0.04)	(0.03)	(0.03)	(0.05)	(0.05)	(0.05)	(0.05)	(0.05)	(0.04)	(0.04)	(0.03)	(0.06)	(0.04)	(0.04)	(0.04)	(0.04)	(0.04)	(0.04)	(0.04)	(0.07)	(0.03)	(0.03)	(0.03)	(0.03)	(0.04)	(0.03)	(0.03)	(0.04)	(0.03)	(0.03)	(0.03)
	PA_2	0.61	0.66	0.73	0.74	0.75	0.64	0.69	0.71	0.71	0.66	0.69	0.71	0.7	0.68	0.71	0.71	0.71	0.73	0.71	0.73	0.74	0.66	0.7	0.7	0.71	0.74	0.72	0.74	0.74	0.67	0.72	0.73	0.73
		(0.05)	(0.04)	(0.03)	(0.03)	(0.03)	(0.05)	(0.03)	(0.04)	(0.05)	(0.05)	(0.04)	(0.04)	(0.04)	(0.06)	(0.04)	(0.04)	(0.04)	(0.04)	(0.05)	(0.04)	(0.04)	(0.07)	(0.04)	(0.04)	(0.04)	(0.03)	(0.04)	(0.03)	(0.03)	(0.04)	(0.04)	(0.04)	(0.04)
	g-means	0.6	0.66	0.73	0.74	0.74	0.63	0.66	0.68	0.69	0.65	0.69	0.7	0.7	0.68	0.7	0.71	0.71	0.73	0.71	0.73	0.74	0.66	0.7	0.71	0.71	0.74	0.72	0.74	0.74	0.67	0.72	0.73	0.73
		(0.02)	(0.02)	(0.02)	(0.02)	(0.02)	(0.03)	(0.02)	(0.03)	(0.03)	(0.03)	(0.02)	(0.02)	(0.02)	(0.02)	(0.02)	(0.02)	(0.02)	(0.02)	(0.03)	(0.02)	(0.02)	(0.02)	(0.02)	(0.02)	(0.02)	(0.02)	(0.02)	(0.02)	(0.02)	(0.02)	(0.02)	(0.02)	(0.02)
	AUC	0.6	0.71	0.8	0.82	0.82	0.69	0.73	0.75	0.76	0.72	0.75	0.77	0.76	0.74	0.77	0.78	0.78	0.81	0.78	0.81	0.82	0.72	0.77	0.78	0.78	0.82	0.8	0.82	0.82	0.74	0.8	0.8	0.8
		(0.03)	(0.03)	(0.02)	(0.02)	(0.02)	(0.03)	(0.02)	(0.04)	(0.03)	(0.03)	(0.03)	(0.03)	(0.02)	(0.03)	(0.03)	(0.02)	(0.02)	(0.02)	(0.03)	(0.02)	(0.02)	(0.03)	(0.02)	(0.02)	(0.02)	(0.02)	(0.02)	(0.02)	(0.02)	(0.02)	(0.02)	(0.02)	(0.02)
300	PA	0.62	0.66	0.74	0.75	0.76	0.65	0.69	0.7	0.7	0.67	0.69	0.7	0.71	0.7	0.71	0.72	0.73	0.76	0.71	0.75	0.76	0.69	0.71	0.72	0.72	0.76	0.74	0.76	0.76	0.69	0.74	0.74	0.74
		(0.02)	(0.02)	(0.02)	(0.02)	(0.02)	(0.02)	(0.02)	(0.01)	(0.02)	(0.03)	(0.02)	(0.02)	(0.02)	(0.02)	(0.01)	(0.02)	(0.02)	(0.02)	(0.03)	(0.02)	(0.02)	(0.02)	(0.02)	(0.02)	(0.02)	(0.02)	(0.02)	(0.02)	(0.02)	(0.02)	(0.02)	(0.02)	(0.02)
	PA_1	0.62	0.66	0.74	0.75	0.76	0.66	0.68	0.69	0.7	0.68	0.69	0.7	0.71	0.69	0.73	0.72	0.72	0.76	0.71	0.75	0.76	0.7	0.72	0.72	0.73	0.76	0.74	0.76	0.76	0.69	0.74	0.74	0.74
		(0.04)	(0.03)	(0.03)	(0.03)	(0.03)	(0.05)	(0.05)	(0.04)	(0.04)	(0.05)	(0.04)	(0.03)	(0.03)	(0.05)	(0.03)	(0.03)	(0.03)	(0.03)	(0.04)	(0.03)	(0.03)	(0.06)	(0.03)	(0.03)	(0.03)	(0.03)	(0.03)	(0.03)	(0.03)	(0.03)	(0.03)	(0.03)	(0.03)
	PA_2	0.62	0.66	0.74	0.75	0.76	0.65	0.7	0.71	0.7	0.67	0.69	0.7	0.71	0.7	0.7	0.72	0.73	0.76	0.71	0.75	0.76	0.68	0.71	0.72	0.72	0.76	0.74	0.75	0.76	0.68	0.74	0.74	0.74
		(0.04)	(0.04)	(0.03)	(0.03)	(0.03)	(0.04)	(0.05)	(0.04)	(0.05)	(0.05)	(0.03)	(0.03)	(0.03)	(0.05)	(0.02)	(0.03)	(0.03)	(0.03)	(0.03)	(0.03)	(0.03)	(0.06)	(0.03)	(0.03)	(0.03)	(0.03)	(0.04)	(0.03)	(0.03)	(0.03)	(0.03)	(0.03)	(0.03)
	g-means	0.62	0.66	0.74	0.75	0.76	0.65	0.69	0.7	0.7	0.67	0.69	0.7	0.71	0.69	0.71	0.72	0.73	0.76	0.71	0.75	0.76	0.69	0.71	0.72	0.72	0.76	0.74	0.76	0.76	0.69	0.73	0.74	0.74
		(0.02)	(0.02)	(0.02)	(0.02)	(0.02)	(0.02)	(0.02)	(0.01)	(0.02)	(0.03)	(0.02)	(0.02)	(0.02)	(0.02)	(0.01)	(0.02)	(0.02)	(0.02)	(0.03)	(0.02)	(0.02)	(0.02)	(0.02)	(0.02)	(0.02)	(0.02)	(0.02)	(0.02)	(0.02)	(0.02)	(0.02)	(0.02)	(0.02)
	AUC	0.61	0.72	0.82	0.83	0.84	0.71	0.76	0.77	0.78	0.74	0.76	0.77	0.78	0.77	0.79	0.8	0.8	0.84	0.79	0.83	0.84	0.75	0.79	0.8	0.8	0.84	0.82	0.84	0.84	0.75	0.81	0.82	0.82
		(0.03)	(0.02)	(0.02)	(0.01)	(0.01)	(0.02)	(0.02)	(0.02)	(0.02)	(0.03)	(0.03)	(0.02)	(0.02)	(0.03)	(0.02)	(0.02)	(0.02)	(0.02)	(0.02)	(0.02)	(0.02)	(0.02)	(0.02)	(0.02)	(0.02)	(0.02)	(0.02)	(0.02)	(0.02)	(0.02)	(0.02)	(0.02)	(0.02)
500	PA	0.63	0.68	0.75	0.76	0.77	0.68	0.7	0.71	0.72	0.7	0.69	0.7	0.71	0.71	0.73	0.74	0.74	0.77	0.73	0.76	0.77	0.71	0.73	0.74	0.74	0.78	0.76	0.77	0.78	0.71	0.75	0.76	0.76
		(0.02)	(0.02)	(0.01)	(0.01)	(0.01)	(0.02)	(0.02)	(0.02)	(0.03)	(0.02)	(0.02)	(0.02)	(0.02)	(0.02)	(0.01)	(0.02)	(0.02)	(0.01)	(0.03)	(0.02)	(0.02)	(0.02)	(0.02)	(0.02)	(0.01)	(0.02)	(0.02)	(0.02)	(0.01)	(0.02)	(0.02)	(0.02)	(0.01)
	PA_1	0.64	0.68	0.75	0.76	0.77	0.68	0.69	0.7	0.72	0.7	0.69	0.7	0.71	0.72	0.72	0.74	0.74	0.78	0.73	0.76	0.77	0.71	0.73	0.74	0.74	0.78	0.76	0.78	0.78	0.71	0.75	0.76	0.76
		(0.04)	(0.03)	(0.02)	(0.02)	(0.02)	(0.04)	(0.04)	(0.05)	(0.05)	(0.04)	(0.03)	(0.03)	(0.03)	(0.05)	(0.02)	(0.03)	(0.03)	(0.02)	(0.03)	(0.03)	(0.03)	(0.05)	(0.03)	(0.02)	(0.03)	(0.02)	(0.03)	(0.02)	(0.02)	(0.03)	(0.03)	(0.03)	(0.03)
	PA_2	0.62	0.68	0.75	0.76	0.77	0.67	0.72	0.72	0.72	0.71	0.69	0.7	0.71	0.7	0.75	0.74	0.74	0.77	0.72	0.76	0.77	0.7	0.73	0.74	0.74	0.77	0.75	0.77	0.77	0.71	0.75	0.76	0.76
		(0.05)	(0.03)	(0.02)	(0.02)	(0.02)	(0.04)	(0.03)	(0.05)	(0.03)	(0.04)	(0.03)	(0.03)	(0.03)	(0.05)	(0.03)	(0.03)	(0.04)	(0.02)	(0.04)	(0.03)	(0.02)	(0.05)	(0.03)	(0.03)	(0.03)	(0.03)	(0.03)	(0.03)	(0.03)	(0.03)	(0.03)	(0.02)	(0.02)
	g-means	0.63	0.68	0.75	0.76	0.77	0.68	0.7	0.71	0.72	0.7	0.69	0.7	0.71	0.71	0.73	0.74	0.74	0.77	0.72	0.76	0.77	0.71	0.73	0.74	0.74	0.78	0.75	0.77	0.78	0.71	0.75	0.76	0.76
		(0.02)	(0.02)	(0.01)	(0.01)	(0.01)	(0.02)	(0.02)	(0.02)	(0.03)	(0.02)	(0.02)	(0.02)	(0.02)	(0.02)	(0.01)	(0.02)	(0.02)	(0.01)	(0.03)	(0.02)	(0.02)	(0.02)	(0.02)	(0.02)	(0.01)	(0.02)	(0.02)	(0.02)	(0.01)	(0.02)	(0.02)	(0.02)	(0.01)
	AUC	0.63	0.74	0.83	0.84	0.85	0.74	0.78	0.78	0.79	0.78	0.76	0.77	0.78	0.79	0.81	0.82	0.82	0.86	0.8	0.84	0.85	0.78	0.81	0.81	0.82	0.86	0.84	0.85	0.86	0.78	0.83	0.83	0.83
		(0.02)	(0.02)	(0.01)	(0.01)	(0.01)	(0.02)	(0.02)	(0.02)	(0.02)	(0.02)	(0.02)	(0.02)	(0.02)	(0.02)	(0.01)	(0.01)	(0.01)	(0.01)	(0.02)	(0.02)	(0.01)	(0.02)	(0.02)	(0.02)	(0.02)	(0.01)	(0.02)	(0.02)	(0.01)	(0.02)	(0.02)	(0.01)	(0.01)
1000	PA	0.65	0.7	0.76	0.77	0.78	0.7	0.72	0.72	0.73	0.72	0.69	0.69	0.7	0.72	0.74	0.74	0.74	0.79	0.73	0.76	0.78	0.73	0.74	0.75	0.75	0.79	0.77	0.79	0.79	0.73	0.76	0.76	0.76
		(0.02)	(0.02)	(0.01)	(0.01)	(0.01)	(0.02)	(0.02)	(0.02)	(0.01)	(0.02)	(0.02)	(0.02)	(0.02)	(0.01)	(0.01)	(0.02)	(0.02)	(0.01)	(0.02)	(0.02)	(0.01)	(0.01)	(0.01)	(0.01)	(0.01)	(0.01)	(0.02)	(0.01)	(0.01)	(0.01)	(0.01)	(0.01)	(0.01)
	PA_1	0.65	0.7	0.76	0.77	0.78	0.7	0.71	0.73	0.74	0.72	0.69	0.69	0.7	0.72	0.76	0.74	0.74	0.79	0.73	0.77	0.78	0.73	0.74	0.75	0.75	0.79	0.77	0.78	0.79	0.73	0.75	0.76	0.76
		(0.04)	(0.03)	(0.02)	(0.02)	(0.02)	(0.03)	(0.04)	(0.04)	(0.04)	(0.04)	(0.03)	(0.03)	(0.03)	(0.05)	(0.05)	(0.05)	(0.06)	(0.02)	(0.03)	(0.02)	(0.02)	(0.05)	(0.02)	(0.02)	(0.02)	(0.02)	(0.02)	(0.02)	(0.02)	(0.02)	(0.02)	(0.02)	(0.02)
	PA_2	0.65	0.7	0.76	0.77	0.77	0.71	0.72	0.71	0.72	0.72	0.69	0.69	0.7	0.73	0.73	0.75	0.75	0.79	0.72	0.76	0.78	0.72	0.74	0.75	0.75	0.79	0.77	0.79	0.79	0.72	0.76	0.76	0.76
		(0.04)	(0.03)	(0.02)	(0.02)	(0.02)	(0.04)	(0.05)	(0.05)	(0.04)	(0.03)	(0.03)	(0.03)	(0.02)	(0.05)	(0.05)	(0.05)	(0.05)	(0.02)	(0.03)	(0.02)	(0.02)	(0.05)	(0.02)	(0.02)	(0.03)	(0.02)	(0.02)	(0.02)	(0.02)	(0.02)	(0.02)	(0.02)	(0.02)
	g-means	0.65	0.7	0.76	0.77	0.78	0.7	0.72	0.72	0.73	0.72	0.69	0.69	0.7	0.72	0.74	0.74	0.74	0.79	0.73	0.76	0.78	0.72	0.74	0.75	0.75	0.79	0.77	0.79	0.79	0.73	0.76	0.76	0.76
		(0.02)	(0.02)	(0.01)	(0.01)	(0.01)	(0.02)	(0.02)	(0.02)	(0.01)	(0.02)	(0.02)	(0.02)	(0.02)	(0.02)	(0.01)	(0.02)	(0.02)	(0.01)	(0.03)	(0.02)	(0.01)	(0.02)	(0.01)	(0.01)	(0.01)	(0.01)	(0.02)	(0.01)	(0.01)	(0.01)	(0.01)	(0.01)	(0.01)
	AUC	0.67	0.77	0.84	0.85	0.86	0.77	0.8	0.8	0.81	0.8	0.76	0.76	0.77	0.8	0.82	0.82	0.82	0.87	0.8	0.85	0.86	0.8	0.82	0.83	0.83	0.87	0.85	0.87	0.87	0.8	0.83	0.84	0.84
		(0.02)	(0.02)	(0.01)	(0.01)	(0.01)	(0.02)	(0.02)	(0.02)	(0.02)	(0.02)	(0.02)	(0.02)	(0.02)	(0.02)	(0.01)	(0.01)	(0.01)	(0.01)	(0.02)	(0.01)	(0.01)	(0.01)	(0.01)	(0.01)	(0.01)	(0.01)	(0.01)	(0.01)	(0.01)	(0.02)	(0.01)	(0.01)	(0.01)

Table 2. Performance of the classifiers for a moderate difference between the classes ($\mu_2 = 1$, $p = 1,000$). The table reports predictive accuracy (PA), predictive accuracy for class 1 and class 2 (PA_1, PA_2), g-means and AUC. Training sets contained 1,000 variables; see the main text for details.

n_{train}		CART(5)	AdaBoost.M1(5).10	AdaBoost.M1(5).100	AdaBoost.M1(5).200	AdaBoost.M1(5).300	AdaBoost.M1.ICV(5).10	AdaBoost.M1.ICV(5).100	AdaBoost.M1.ICV(5).200	AdaBoost.M1.ICV(5).300	LogitBoost(1).10	LogitBoost(1).100	LogitBoost(1).200	LogitBoost(1).300	AdaBoost.M1(1).10	AdaBoost.M1(1).100	AdaBoost.M1(1).200	AdaBoost.M1(1).300	St-GrBoost(1).opt	St-GrBoost(1).100	St-GrBoost(1).300	St-GrBoost(1).500	GrBoost(1).10	GrBoost(1).100	GrBoost(1).200	GrBoost(1).300	St-GrBoost(5).opt	St-GrBoost(5).100	St-GrBoost(5).300	St-GrBoost(5).500	GrBoost(5).10	GrBoost(5).100	GrBoost(5).200	GrBoost(5).300
50	PA	0.73	0.72	0.73	0.73	0.73	0.76	0.82	0.83	0.85	0.8	0.83	0.82	0.82	0.8	0.85	0.86	0.86	0.88	0.85	0.87	0.88	0.79	0.84	0.85	0.85	0.87	0.86	0.88	0.88	0.73	0.72	0.72	0.71
		(0.03)	(0.03)	(0.03)	(0.03)	(0.03)	(0.04)	(0.03)	(0.03)	(0.03)	(0.04)	(0.03)	(0.04)	(0.04)	(0.04)	(0.04)	(0.03)	(0.03)	(0.03)	(0.04)	(0.03)	(0.03)	(0.04)	(0.03)	(0.03)	(0.03)	(0.04)	(0.04)	(0.04)	(0.04)	(0.04)	(0.04)	(0.04)	(0.04)
	PA_1	0.73	0.73	0.74	0.74	0.74	0.76	0.82	0.82	0.85	0.79	0.81	0.8	0.8	0.81	0.85	0.86	0.85	0.88	0.85	0.87	0.88	0.79	0.84	0.85	0.85	0.87	0.86	0.88	0.88	0.73	0.72	0.72	0.71
		(0.07)	(0.07)	(0.07)	(0.07)	(0.07)	(0.07)	(0.07)	(0.04)	(0.05)	(0.06)	(0.05)	(0.06)	(0.06)	(0.07)	(0.05)	(0.05)	(0.05)	(0.04)	(0.06)	(0.05)	(0.04)	(0.07)	(0.05)	(0.05)	(0.05)	(0.05)	(0.05)	(0.05)	(0.05)	(0.07)	(0.07)	(0.07)	(0.07)
	PA_2	0.72	0.72	0.71	0.71	0.71	0.76	0.82	0.84	0.86	0.81	0.85	0.83	0.84	0.79	0.85	0.86	0.86	0.88	0.85	0.87	0.88	0.79	0.84	0.84	0.84	0.87	0.86	0.88	0.88	0.73	0.71	0.71	0.71
		(0.08)	(0.08)	(0.07)	(0.07)	(0.07)	(0.06)	(0.07)	(0.06)	(0.05)	(0.05)	(0.06)	(0.07)	(0.06)	(0.06)	(0.06)	(0.05)	(0.05)	(0.05)	(0.06)	(0.05)	(0.05)	(0.07)	(0.05)	(0.05)	(0.05)	(0.05)	(0.06)	(0.05)	(0.05)	(0.07)	(0.06)	(0.06)	(0.06)
	g-means	0.72	0.72	0.73	0.73	0.73	0.76	0.82	0.83	0.85	0.8	0.83	0.81	0.82	0.8	0.85	0.86	0.86	0.88	0.85	0.87	0.88	0.79	0.84	0.85	0.85	0.87	0.86	0.88	0.88	0.73	0.71	0.71	0.71
		(0.03)	(0.03)	(0.03)	(0.03)	(0.03)	(0.04)	(0.03)	(0.03)	(0.03)	(0.04)	(0.03)	(0.04)	(0.04)	(0.04)	(0.04)	(0.03)	(0.03)	(0.03)	(0.04)	(0.03)	(0.03)	(0.04)	(0.03)	(0.03)	(0.03)	(0.04)	(0.04)	(0.04)	(0.04)	(0.04)	(0.04)	(0.04)	(0.04)
	AUC	0.73	0.72	0.73	0.73	0.73	0.84	0.91	0.92	0.94	0.88	0.9	0.87	0.87	0.89	0.93	0.94	0.94	0.95	0.93	0.95	0.95	0.87	0.92	0.92	0.92	0.95	0.94	0.95	0.96	0.8	0.76	0.75	0.75
		(0.03)	(0.03)	(0.03)	(0.03)	(0.03)	(0.04)	(0.02)	(0.03)	(0.02)	(0.04)	(0.04)	(0.06)	(0.06)	(0.04)	(0.03)	(0.02)	(0.02)	(0.02)	(0.03)	(0.02)	(0.02)	(0.04)	(0.03)	(0.03)	(0.03)	(0.03)	(0.03)	(0.02)	(0.02)	(0.05)	(0.04)	(0.04)	(0.04)
100	PA	0.74	0.74	0.74	0.74	0.74	0.81	0.86	0.88	0.89	0.84	0.89	0.88	0.88	0.84	0.91	0.9	0.9	0.91	0.86	0.9	0.91	0.83	0.89	0.9	0.9	0.91	0.89	0.91	0.91	0.83	0.88	0.88	0.88
		(0.03)	(0.03)	(0.03)	(0.03)	(0.03)	(0.03)	(0.03)	(0.02)	(0.01)	(0.02)	(0.01)	(0.02)	(0.02)	(0.02)	(0.01)	(0.01)	(0.01)	(0.01)	(0.03)	(0.02)	(0.02)	(0.03)	(0.02)	(0.02)	(0.02)	(0.03)	(0.03)	(0.02)	(0.02)	(0.03)	(0.04)	(0.05)	(0.05)
	PA_1	0.74	0.74	0.73	0.73	0.73	0.81	0.85	0.87	0.89	0.83	0.87	0.87	0.87	0.85	0.91	0.9	0.9	0.91	0.86	0.9	0.91	0.84	0.89	0.9	0.9	0.9	0.88	0.9	0.91	0.83	0.88	0.88	0.88
		(0.05)	(0.05)	(0.06)	(0.06)	(0.06)	(0.04)	(0.04)	(0.03)	(0.02)	(0.04)	(0.03)	(0.03)	(0.03)	(0.04)	(0.03)	(0.02)	(0.02)	(0.03)	(0.05)	(0.03)	(0.03)	(0.06)	(0.03)	(0.03)	(0.03)	(0.04)	(0.05)	(0.04)	(0.04)	(0.05)	(0.05)	(0.05)	(0.05)
	PA_2	0.74	0.74	0.74	0.74	0.74	0.81	0.87	0.89	0.9	0.85	0.9	0.89	0.89	0.84	0.9	0.9	0.9	0.92	0.86	0.9	0.91	0.82	0.89	0.9	0.9	0.91	0.89	0.91	0.91	0.83	0.88	0.88	0.88
		(0.06)	(0.06)	(0.06)	(0.06)	(0.06)	(0.05)	(0.04)	(0.03)	(0.03)	(0.04)	(0.03)	(0.03)	(0.03)	(0.05)	(0.02)	(0.02)	(0.02)	(0.02)	(0.04)	(0.03)	(0.03)	(0.06)	(0.03)	(0.03)	(0.03)	(0.03)	(0.03)	(0.02)	(0.02)	(0.05)	(0.05)	(0.05)	(0.06)
	g-means	0.74	0.74	0.74	0.74	0.74	0.81	0.86	0.88	0.89	0.84	0.89	0.88	0.88	0.84	0.91	0.9	0.9	0.91	0.86	0.9	0.91	0.83	0.89	0.9	0.9	0.91	0.89	0.91	0.91	0.83	0.88	0.88	0.88
		(0.03)	(0.03)	(0.03)	(0.03)	(0.03)	(0.03)	(0.03)	(0.02)	(0.01)	(0.02)	(0.01)	(0.02)	(0.02)	(0.02)	(0.01)	(0.01)	(0.01)	(0.01)	(0.03)	(0.02)	(0.02)	(0.03)	(0.02)	(0.02)	(0.02)	(0.03)	(0.03)	(0.02)	(0.02)	(0.03)	(0.04)	(0.05)	(0.05)
	AUC	0.74	0.74	0.74	0.74	0.74	0.89	0.94	0.95	0.96	0.92	0.96	0.94	0.94	0.93	0.97	0.97	0.97	0.97	0.94	0.96	0.97	0.91	0.96	0.96	0.96	0.97	0.96	0.97	0.97	0.91	0.94	0.93	0.93
		(0.03)	(0.03)	(0.03)	(0.03)	(0.03)	(0.03)	(0.02)	(0.01)	(0.01)	(0.02)	(0.01)	(0.02)	(0.02)	(0.02)	(0.01)	(0.01)	(0.01)	(0.01)	(0.02)	(0.01)	(0.01)	(0.02)	(0.01)	(0.01)	(0.01)	(0.01)	(0.02)	(0.01)	(0.01)	(0.04)	(0.05)	(0.05)	(0.05)
200	PA	0.76	0.86	0.91	0.92	0.92	0.85	0.88	0.9	0.91	0.86	0.91	0.91	0.91	0.87	0.91	0.92	0.92	0.93	0.87	0.91	0.93	0.85	0.91	0.92	0.92	0.93	0.91	0.93	0.93	0.88	0.92	0.93	0.93
		(0.02)	(0.04)	(0.05)	(0.06)	(0.06)	(0.02)	(0.02)	(0.01)	(0.01)	(0.02)	(0.01)	(0.01)	(0.01)	(0.02)	(0.01)	(0.01)	(0.01)	(0.01)	(0.03)	(0.01)	(0.01)	(0.02)	(0.01)	(0.01)	(0.01)	(0.01)	(0.02)	(0.01)	(0.01)	(0.01)	(0.01)	(0.01)	(0.01)
	PA_1	0.77	0.86	0.91	0.91	0.92	0.85	0.88	0.9	0.9	0.86	0.91	0.9	0.9	0.86	0.91	0.92	0.92	0.94	0.88	0.92	0.93	0.86	0.91	0.92	0.92	0.93	0.91	0.93	0.93	0.88	0.92	0.93	0.93
		(0.04)	(0.04)	(0.06)	(0.06)	(0.06)	(0.03)	(0.03)	(0.02)	(0.03)	(0.03)	(0.02)	(0.02)	(0.03)	(0.04)	(0.02)	(0.02)	(0.02)	(0.02)	(0.03)	(0.02)	(0.02)	(0.04)	(0.02)	(0.02)	(0.02)	(0.02)	(0.03)	(0.02)	(0.02)	(0.03)	(0.02)	(0.02)	(0.02)
	PA_2	0.76	0.85	0.91	0.92	0.92	0.85	0.88	0.9	0.92	0.87	0.92	0.91	0.91	0.87	0.92	0.92	0.92	0.93	0.87	0.91	0.92	0.85	0.91	0.92	0.92	0.93	0.91	0.93	0.93	0.87	0.92	0.92	0.93
		(0.04)	(0.05)	(0.06)	(0.06)	(0.06)	(0.03)	(0.03)	(0.02)	(0.02)	(0.03)	(0.02)	(0.02)	(0.02)	(0.03)	(0.02)	(0.02)	(0.02)	(0.02)	(0.04)	(0.02)	(0.02)	(0.04)	(0.02)	(0.02)	(0.02)	(0.02)	(0.03)	(0.02)	(0.02)	(0.02)	(0.02)	(0.02)	(0.02)
	g-means	0.76	0.86	0.91	0.92	0.92	0.85	0.88	0.9	0.91	0.86	0.91	0.91	0.91	0.87	0.91	0.92	0.92	0.93	0.87	0.91	0.93	0.85	0.91	0.92	0.92	0.93	0.91	0.93	0.93	0.88	0.92	0.93	0.93
		(0.02)	(0.04)	(0.06)	(0.06)	(0.06)	(0.02)	(0.02)	(0.01)	(0.01)	(0.02)	(0.01)	(0.01)	(0.01)	(0.02)	(0.01)	(0.01)	(0.01)	(0.01)	(0.03)	(0.01)	(0.01)	(0.02)	(0.01)	(0.01)	(0.01)	(0.01)	(0.02)	(0.01)	(0.01)	(0.01)	(0.01)	(0.01)	(0.01)
	AUC	0.76	0.93	0.96	0.97	0.97	0.92	0.95	0.96	0.97	0.94	0.97	0.97	0.96	0.94	0.98	0.98	0.98	0.98	0.95	0.97	0.98	0.93	0.97	0.98	0.98	0.98	0.97	0.98	0.98	0.95	0.98	0.97	0.97
		(0.03)	(0.04)	(0.05)	(0.05)	(0.05)	(0.02)	(0.02)	(0.01)	(0)	(0.01)	(0.01)	(0.01)	(0.01)	(0.01)	(0)	(0)	(0)	(0)	(0.02)	(0.01)	(0)	(0.01)	(0.01)	(0.01)	(0.01)	(0.01)	(0.01)	(0.01)	(0.01)	(0.01)	(0.01)	(0.01)	(0.01)
300	PA	0.77	0.88	0.93	0.94	0.94	0.86	0.89	0.91	0.91	0.87	0.92	0.92	0.91	0.87	0.93	0.93	0.93	0.94	0.88	0.92	0.93	0.87	0.92	0.93	0.93	0.94	0.92	0.94	0.94	0.89	0.93	0.93	0.93
		(0.02)	(0.01)	(0.02)	(0.02)	(0.02)	(0.02)	(0.01)	(0.01)	(0.01)	(0.02)	(0.01)	(0.01)	(0.01)	(0.01)	(0.01)	(0.01)	(0.01)	(0.01)	(0.03)	(0.01)	(0.01)	(0.02)	(0.01)	(0.01)	(0.01)	(0.01)	(0.01)	(0.01)	(0.01)	(0.01)	(0.01)	(0.01)	(0.01)
	PA_1	0.78	0.88	0.93	0.94	0.94	0.86	0.9	0.91	0.91	0.87	0.91	0.91	0.91	0.87	0.93	0.93	0.93	0.94	0.88	0.92	0.93	0.86	0.92	0.93	0.93	0.94	0.92	0.94	0.94	0.89	0.93	0.93	0.93
		(0.03)	(0.02)	(0.02)	(0.02)	(0.02)	(0.03)	(0.02)	(0.03)	(0.02)	(0.03)	(0.02)	(0.02)	(0.02)	(0.03)	(0.02)	(0.02)	(0.01)	(0.01)	(0.03)	(0.02)	(0.01)	(0.04)	(0.02)	(0.02)	(0.02)	(0.01)	(0.02)	(0.02)	(0.02)	(0.02)	(0.01)	(0.01)	(0.01)
	PA_2	0.77	0.88	0.93	0.94	0.94	0.86	0.89	0.9	0.91	0.87	0.92	0.92	0.92	0.87	0.93	0.93	0.93	0.94	0.88	0.92	0.93	0.87	0.92	0.92	0.93	0.94	0.92	0.93	0.94	0.89	0.93	0.93	0.93
		(0.04)	(0.02)	(0.02)	(0.02)	(0.02)	(0.02)	(0.02)	(0.02)	(0.02)	(0.03)	(0.02)	(0.02)	(0.02)	(0.03)	(0.02)	(0.02)	(0.02)	(0.02)	(0.03)	(0.02)	(0.02)	(0.04)	(0.02)	(0.02)	(0.02)	(0.01)	(0.02)	(0.02)	(0.01)	(0.02)	(0.01)	(0.01)	(0.01)
	g-means	0.77	0.88	0.93	0.94	0.94	0.86	0.89	0.9	0.91	0.87	0.92	0.92	0.91	0.87	0.93	0.93	0.93	0.94	0.88	0.92	0.93	0.87	0.92	0.93	0.93	0.94	0.92	0.94	0.94	0.89	0.93	0.93	0.93
		(0.02)	(0.01)	(0.02)	(0.02)	(0.02)	(0.02)	(0.01)	(0.01)	(0.01)	(0.02)	(0.01)	(0.01)	(0.01)	(0.01)	(0.01)	(0.01)	(0.01)	(0.01)	(0.03)	(0.01)	(0.01)	(0.02)	(0.01)	(0.01)	(0.01)	(0.01)	(0.01)	(0.01)	(0.01)	(0.01)	(0.01)	(0.01)	(0.01)
	AUC	0.76	0.95	0.98	0.98	0.98	0.93	0.96	0.97	0.97	0.95	0.97	0.97	0.97	0.95	0.98	0.98	0.98	0.98	0.95	0.98	0.98	0.94	0.98	0.98	0.98	0.99	0.98	0.98	0.99	0.96	0.98	0.98	0.98
		(0.03)	(0.01)	(0.02)	(0.02)	(0.02)	(0.01)	(0.01)	(0.01)	(0)	(0.01)	(0.01)	(0.01)	(0.01)	(0.01)	(0)	(0)	(0)	(0)	(0.02)	(0.01)	(0.01)	(0.01)	(0)	(0)	(0)	(0)	(0.01)	(0)	(0)	(0.01)	(0)	(0)	(0.01)
500	PA	0.79	0.89	0.94	0.94	0.95	0.88	0.9	0.91	0.92	0.88	0.92	0.93	0.92	0.88	0.93	0.93	0.93	0.94	0.89	0.93	0.93	0.87	0.93	0.93	0.93	0.95	0.93	0.94	0.94	0.9	0.93	0.94	0.94
		(0.02)	(0.01)	(0.01)	(0.01)	(0.01)	(0.01)	(0.02)	(0.01)	(0.01)	(0.01)	(0.01)	(0.01)	(0.01)	(0.01)	(0.01)	(0.01)	(0.01)	(0.01)	(0.02)	(0.01)	(0.01)	(0.02)	(0.01)	(0.01)	(0.01)	(0.01)	(0.01)	(0.01)	(0.01)	(0.01)	(0.01)	(0.01)	(0.01)
	PA_1	0.79	0.89	0.94	0.95	0.95	0.88	0.9	0.92	0.91	0.88	0.92	0.92	0.92	0.88	0.92	0.93	0.93	0.94	0.89	0.92	0.93	0.88	0.93	0.93	0.93	0.95	0.92	0.94	0.94	0.9	0.93	0.94	0.94
		(0.03)	(0.02)	(0.01)	(0.01)	(0.01)	(0.02)	(0.02)	(0.01)	(0.03)	(0.02)	(0.01)	(0.01)	(0.01)	(0.03)	(0.02)	(0.02)	(0.02)	(0.01)	(0.03)	(0.01)	(0.01)	(0.04)	(0.01)	(0.01)	(0.01)	(0.01)	(0.02)	(0.01)	(0.01)	(0.02)	(0.01)	(0.01)	(0.01)
	PA_2	0.79	0.89	0.94	0.94	0.95	0.88	0.9	0.91	0.92	0.88	0.92	0.93	0.93	0.88	0.93	0.93	0.93	0.94	0.89	0.93	0.94	0.87	0.93	0.93	0.93	0.95	0.93	0.94	0.94	0.9	0.93	0.94	0.94
		(0.03)	(0.02)	(0.01)	(0.01)	(0.01)	(0.02)	(0.03)	(0.02)	(0.02)	(0.02)	(0.02)	(0.01)	(0.01)	(0.03)	(0.02)	(0.01)	(0.01)	(0.02)	(0.03)	(0.02)	(0.01)	(0.04)	(0.01)	(0.01)	(0.01)	(0.01)	(0.02)	(0.01)	(0.01)	(0.02)	(0.01)	(0.01)	(0.02)
	g-means	0.79	0.89	0.94	0.94	0.95	0.88	0.9	0.91	0.92	0.88	0.92	0.93	0.92	0.88	0.93	0.93	0.93	0.94	0.89	0.93	0.93	0.87	0.93	0.93	0.93	0.95	0.93	0.94	0.94	0.9	0.93	0.94	0.94
		(0.02)	(0.01)	(0.01)	(0.01)	(0.01)	(0.01)	(0.02)	(0.01)	(0.01)	(0.01)	(0.01)	(0.01)	(0.01)	(0.01)	(0.01)	(0.01)	(0.01)	(0.01)	(0.02)	(0.01)	(0.01)	(0.02)	(0.01)	(0.01)	(0.01)	(0.01)	(0.01)	(0.01)	(0.01)	(0.01)	(0.01)	(0.01)	(0.01)
	AUC	0.79	0.96	0.99	0.99	0.99	0.95	0.96	0.97	0.98	0.95	0.98	0.98	0.98	0.95	0.98	0.98	0.98	0.99	0.96	0.98	0.98	0.94	0.98	0.98	0.98	0.99	0.98	0.99	0.99	0.96	0.98	0.98	0.98
		(0.02)	(0.01)	(0)	(0)	(0)	(0.01)	(0.01)	(0.01)	(0.01)	(0.01)	(0)	(0)	(0)	(0.01)	(0)	(0)	(0)	(0)	(0.01)	(0.01)	(0.01)	(0.01)	(0)	(0)	(0)	(0)	(0.01)	(0)	(0)	(0.01)	(0)	(0)	(0)
1000	PA	0.8	0.9	0.95	0.95	0.95	0.89	0.91	0.92	0.92	0.89	0.92	0.93	0.93	0.88	0.94	0.94	0.94	0.94	0.9	0.93	0.94	0.89	0.93	0.94	0.94	0.95	0.93	0.95	0.95	0.9	0.94	0.94	0.94
		(0.01)	(0.01)	(0.01)	(0.01)	(0.01)	(0.01)	(0.01)	(0.01)	(0.01)	(0.01)	(0.01)	(0.01)	(0.01)	(0.01)	(0.01)	(0.01)	(0.01)	(0.01)	(0.02)	(0.01)	(0.01)	(0.01)	(0.01)	(0.01)	(0.01)	(0.01)	(0.01)	(0.01)	(0.01)	(0.01)	(0.01)	(0.01)	(0.01)
	PA_1	0.8	0.9	0.94	0.95	0.95	0.89	0.91	0.92	0.92	0.88	0.92	0.92	0.93	0.88	0.94	0.94	0.94	0.95	0.9	0.93	0.94	0.89	0.93	0.94	0.94	0.95	0.94	0.95	0.95	0.9	0.94	0.94	0.94
		(0.03)	(0.02)	(0.01)	(0.01)	(0.01)	(0.02)	(0.02)	(0.02)	(0.02)	(0.02)	(0.02)	(0.02)	(0.01)	(0.02)	(0.02)	(0.01)	(0.01)	(0.01)	(0.02)	(0.01)	(0.01)	(0.03)	(0.01)	(0.01)	(0.01)	(0.01)	(0.01)	(0.01)	(0.01)	(0.01)	(0.01)	(0.01)	(0.01)
	PA_2	0.8	0.9	0.95	0.95	0.95	0.89	0.91	0.92	0.92	0.89	0.92	0.93	0.93	0.88	0.94	0.94	0.94	0.94	0.9	0.93	0.94	0.88	0.93	0.93	0.94	0.95	0.93	0.94	0.95	0.9	0.94	0.94	0.94
		(0.03)	(0.02)	(0.01)	(0.01)	(0.01)	(0.02)	(0.02)	(0.03)	(0.02)	(0.02)	(0.01)	(0.01)	(0.01)	(0.02)	(0.01)	(0.01)	(0.01)	(0.01)	(0.02)	(0.01)	(0.01)	(0.03)	(0.01)	(0.01)	(0.01)	(0.01)	(0.01)	(0.01)	(0.01)	(0.01)	(0.01)	(0.01)	(0.01)
	g-means	0.8	0.9	0.95	0.95	0.95	0.89	0.91	0.92	0.92	0.89	0.92	0.93	0.93	0.88	0.94	0.94	0.94	0.94	0.9	0.93	0.94	0.89	0.93	0.94	0.94	0.95	0.93	0.95	0.95	0.9	0.94	0.94	0.94
		(0.01)	(0.01)	(0.01)	(0.01)	(0.01)	(0.01)	(0.01)	(0.01)	(0.01)	(0.01)	(0.01)	(0.01)	(0.01)	(0.01)	(0.01)	(0.01)	(0.01)	(0.01)	(0.02)	(0.01)	(0.01)	(0.01)	(0.01)	(0.01)	(0.01)	(0.01)	(0.01)	(0.01)	(0.01)	(0.01)	(0.01)	(0.01)	(0.01)
	AUC	0.82	0.97	0.99	0.99	0.99	0.95	0.97	0.98	0.98	0.96	0.98	0.98	0.98	0.96	0.99	0.99	0.99	0.99	0.97	0.98	0.99	0.95	0.98	0.98	0.98	0.99	0.98	0.99	0.99	0.97	0.99	0.99	0.99
		(0.02)	(0.01)	(0)	(0)	(0)	(0.01)	(0.01)	(0.01)	(0)	(0.01)	(0)	(0)	(0)	(0.01)	(0)	(0)	(0)	(0)	(0.01)	(0)	(0)	(0.01)	(0)	(0)	(0)	(0)	(0)	(0)	(0)	(0)	(0)	(0)	(0)

Table 3. Performance of the classifiers for a small difference between the classes and a large number of variables ($\mu_2 = 0.5$, $p = 2,500$). The table reports predictive accuracy (PA), predictive accuracy for class 1 and class 2 (PA_1, PA_2), g-means and AUC. Training sets contained 2,500 variables; see the main text for details.

n_{train}		CART(5)	AdaBoost.M1(5).10	AdaBoost.M1(5).100	AdaBoost.M1(5).200	AdaBoost.M1(5).300	AdaBoost.M1.ICV(5).10	AdaBoost.M1.ICV(5).100	AdaBoost.M1.ICV(5).200	AdaBoost.M1.ICV(5).300	LogitBoost(1).10	LogitBoost(1).100	LogitBoost(1).200	LogitBoost(1).300	AdaBoost.M1(1).10	AdaBoost.M1(1).100	AdaBoost.M1(1).200	AdaBoost.M1(1).300	St-GrBoost(1).opt	St-GrBoost(1).100	St-GrBoost(1).300	St-GrBoost(1).500	GrBoost(1).10	GrBoost(1).100	GrBoost(1).200	GrBoost(1).300	St-GrBoost(5).opt	St-GrBoost(5).100	St-GrBoost(5).300	St-GrBoost(5).500	GrBoost(5).10	GrBoost(5).100	GrBoost(5).200	GrBoost(5).300
50	PA	0.55	0.54	0.54	0.54	0.54	0.54	0.55	0.56	0.56	0.55	0.57	0.57	0.57	0.54	0.56	0.56	0.56	0.55	0.58	0.58	0.58	0.55	0.57	0.57	0.57	0.57	0.58	0.59	0.59	0.54	0.54	0.54	0.54
		(0.04)	(0.03)	(0.03)	(0.03)	(0.03)	(0.03)	(0.03)	(0.04)	(0.04)	(0.03)	(0.03)	(0.03)	(0.03)	(0.04)	(0.03)	(0.03)	(0.03)	(0.05)	(0.04)	(0.04)	(0.04)	(0.03)	(0.03)	(0.03)	(0.03)	(0.05)	(0.04)	(0.04)	(0.04)	(0.04)	(0.04)	(0.04)	(0.04)
	PA_1	0.54	0.54	0.54	0.54	0.54	0.56	0.56	0.57	0.58	0.53	0.56	0.56	0.56	0.56	0.57	0.58	0.58	0.53	0.57	0.58	0.58	0.54	0.56	0.56	0.56	0.57	0.59	0.6	0.6	0.54	0.53	0.53	0.53
		(0.08)	(0.08)	(0.08)	(0.08)	(0.08)	(0.07)	(0.06)	(0.05)	(0.06)	(0.07)	(0.07)	(0.06)	(0.06)	(0.07)	(0.06)	(0.06)	(0.07)	(0.13)	(0.09)	(0.08)	(0.07)	(0.08)	(0.07)	(0.07)	(0.07)	(0.1)	(0.08)	(0.07)	(0.07)	(0.07)	(0.07)	(0.07)	(0.07)
	PA_2	0.55	0.54	0.54	0.54	0.54	0.51	0.54	0.54	0.55	0.56	0.58	0.58	0.58	0.52	0.54	0.54	0.54	0.57	0.58	0.58	0.58	0.56	0.57	0.58	0.58	0.56	0.58	0.58	0.59	0.55	0.55	0.55	0.55
		(0.07)	(0.06)	(0.06)	(0.06)	(0.06)	(0.08)	(0.06)	(0.06)	(0.06)	(0.07)	(0.07)	(0.06)	(0.06)	(0.08)	(0.06)	(0.06)	(0.06)	(0.11)	(0.08)	(0.07)	(0.07)	(0.08)	(0.06)	(0.06)	(0.07)	(0.09)	(0.08)	(0.07)	(0.06)	(0.07)	(0.08)	(0.07)	(0.07)
	g-means	0.54	0.54	0.54	0.54	0.54	0.53	0.55	0.55	0.56	0.54	0.57	0.57	0.57	0.54	0.56	0.56	0.56	0.54	0.57	0.58	0.58	0.55	0.56	0.57	0.57	0.56	0.58	0.59	0.59	0.54	0.54	0.54	0.54
		(0.04)	(0.03)	(0.03)	(0.03)	(0.03)	(0.03)	(0.03)	(0.04)	(0.04)	(0.03)	(0.03)	(0.03)	(0.03)	(0.04)	(0.03)	(0.03)	(0.03)	(0.06)	(0.04)	(0.04)	(0.04)	(0.03)	(0.03)	(0.03)	(0.03)	(0.05)	(0.04)	(0.04)	(0.04)	(0.04)	(0.04)	(0.04)	(0.04)
	AUC	0.55	0.54	0.54	0.54	0.54	0.56	0.57	0.58	0.59	0.57	0.6	0.59	0.59	0.56	0.58	0.59	0.59	0.57	0.61	0.61	0.61	0.57	0.59	0.6	0.6	0.59	0.62	0.63	0.63	0.56	0.55	0.55	0.55
		(0.04)	(0.03)	(0.03)	(0.03)	(0.03)	(0.04)	(0.04)	(0.05)	(0.05)	(0.04)	(0.04)	(0.05)	(0.05)	(0.05)	(0.04)	(0.04)	(0.04)	(0.07)	(0.05)	(0.05)	(0.05)	(0.04)	(0.04)	(0.04)	(0.04)	(0.06)	(0.05)	(0.05)	(0.05)	(0.05)	(0.04)	(0.04)	(0.04)
100	PA	0.55	0.54	0.54	0.54	0.54	0.56	0.59	0.6	0.61	0.57	0.59	0.59	0.59	0.57	0.59	0.6	0.6	0.59	0.61	0.62	0.62	0.57	0.6	0.6	0.6	0.62	0.62	0.63	0.63	0.58	0.61	0.61	0.61
		(0.03)	(0.03)	(0.03)	(0.03)	(0.03)	(0.02)	(0.03)	(0.03)	(0.03)	(0.03)	(0.03)	(0.03)	(0.03)	(0.02)	(0.03)	(0.02)	(0.02)	(0.05)	(0.03)	(0.03)	(0.03)	(0.04)	(0.03)	(0.03)	(0.04)	(0.04)	(0.03)	(0.03)	(0.03)	(0.03)	(0.03)	(0.03)	(0.03)
	PA_1	0.56	0.54	0.54	0.54	0.54	0.54	0.57	0.58	0.59	0.56	0.59	0.59	0.59	0.55	0.58	0.59	0.58	0.58	0.61	0.62	0.61	0.57	0.6	0.61	0.61	0.63	0.63	0.63	0.63	0.57	0.6	0.6	0.6
		(0.05)	(0.06)	(0.06)	(0.06)	(0.06)	(0.06)	(0.04)	(0.05)	(0.05)	(0.06)	(0.05)	(0.05)	(0.05)	(0.06)	(0.04)	(0.03)	(0.04)	(0.11)	(0.06)	(0.05)	(0.05)	(0.08)	(0.05)	(0.06)	(0.05)	(0.06)	(0.06)	(0.05)	(0.05)	(0.06)	(0.06)	(0.06)	(0.06)
	PA_2	0.54	0.54	0.54	0.54	0.54	0.59	0.61	0.62	0.63	0.57	0.59	0.59	0.59	0.59	0.61	0.61	0.61	0.61	0.61	0.62	0.62	0.57	0.59	0.6	0.6	0.61	0.62	0.62	0.62	0.58	0.62	0.62	0.62
		(0.06)	(0.06)	(0.06)	(0.06)	(0.06)	(0.08)	(0.05)	(0.04)	(0.05)	(0.06)	(0.05)	(0.06)	(0.06)	(0.06)	(0.04)	(0.04)	(0.04)	(0.09)	(0.06)	(0.05)	(0.05)	(0.09)	(0.05)	(0.05)	(0.05)	(0.07)	(0.06)	(0.05)	(0.05)	(0.06)	(0.05)	(0.06)	(0.06)
	g-means	0.55	0.54	0.54	0.54	0.54	0.56	0.59	0.6	0.6	0.56	0.59	0.59	0.59	0.57	0.59	0.6	0.6	0.59	0.61	0.62	0.61	0.57	0.59	0.6	0.6	0.62	0.62	0.63	0.63	0.58	0.6	0.61	0.61
		(0.03)	(0.03)	(0.03)	(0.03)	(0.03)	(0.02)	(0.03)	(0.03)	(0.03)	(0.03)	(0.03)	(0.03)	(0.03)	(0.03)	(0.03)	(0.02)	(0.02)	(0.07)	(0.03)	(0.03)	(0.03)	(0.04)	(0.03)	(0.03)	(0.04)	(0.04)	(0.03)	(0.03)	(0.03)	(0.03)	(0.03)	(0.03)	(0.03)
	AUC	0.55	0.55	0.55	0.55	0.55	0.59	0.63	0.64	0.65	0.59	0.62	0.63	0.63	0.6	0.63	0.64	0.64	0.63	0.66	0.66	0.66	0.6	0.63	0.64	0.64	0.66	0.67	0.68	0.68	0.61	0.64	0.64	0.64
		(0.03)	(0.03)	(0.03)	(0.03)	(0.03)	(0.03)	(0.04)	(0.04)	(0.04)	(0.04)	(0.04)	(0.04)	(0.04)	(0.03)	(0.04)	(0.03)	(0.03)	(0.07)	(0.04)	(0.04)	(0.04)	(0.05)	(0.04)	(0.04)	(0.04)	(0.06)	(0.04)	(0.04)	(0.04)	(0.04)	(0.04)	(0.04)	(0.04)
200	PA	0.57	0.57	0.62	0.63	0.63	0.59	0.61	0.62	0.63	0.59	0.6	0.61	0.61	0.6	0.6	0.6	0.61	0.66	0.65	0.66	0.66	0.6	0.62	0.62	0.62	0.66	0.66	0.66	0.66	0.61	0.63	0.64	0.64
		(0.02)	(0.03)	(0.03)	(0.04)	(0.04)	(0.02)	(0.03)	(0.02)	(0.03)	(0.02)	(0.02)	(0.02)	(0.02)	(0.02)	(0.02)	(0.02)	(0.02)	(0.02)	(0.02)	(0.02)	(0.02)	(0.02)	(0.02)	(0.02)	(0.02)	(0.02)	(0.02)	(0.02)	(0.02)	(0.03)	(0.03)	(0.02)	(0.02)
	PA_1	0.57	0.58	0.63	0.64	0.64	0.59	0.62	0.63	0.63	0.59	0.6	0.61	0.61	0.6	0.62	0.62	0.62	0.65	0.65	0.66	0.65	0.6	0.62	0.63	0.63	0.66	0.66	0.66	0.66	0.6	0.64	0.65	0.65
		(0.05)	(0.06)	(0.04)	(0.05)	(0.05)	(0.06)	(0.06)	(0.04)	(0.04)	(0.05)	(0.04)	(0.04)	(0.04)	(0.06)	(0.04)	(0.04)	(0.04)	(0.04)	(0.05)	(0.04)	(0.04)	(0.08)	(0.04)	(0.04)	(0.04)	(0.04)	(0.04)	(0.04)	(0.04)	(0.04)	(0.04)	(0.04)	(0.04)
	PA_2	0.58	0.57	0.61	0.62	0.63	0.58	0.6	0.62	0.62	0.59	0.61	0.61	0.61	0.61	0.59	0.59	0.6	0.66	0.66	0.66	0.66	0.61	0.61	0.61	0.62	0.66	0.65	0.66	0.66	0.61	0.63	0.64	0.64
		(0.05)	(0.05)	(0.04)	(0.04)	(0.04)	(0.06)	(0.08)	(0.06)	(0.06)	(0.05)	(0.05)	(0.04)	(0.05)	(0.06)	(0.04)	(0.04)	(0.04)	(0.04)	(0.04)	(0.04)	(0.04)	(0.07)	(0.04)	(0.04)	(0.04)	(0.04)	(0.04)	(0.04)	(0.04)	(0.04)	(0.04)	(0.04)	(0.04)
	g-means	0.57	0.57	0.62	0.63	0.63	0.59	0.61	0.62	0.62	0.59	0.6	0.61	0.61	0.6	0.6	0.6	0.61	0.66	0.65	0.66	0.66	0.6	0.62	0.62	0.62	0.66	0.65	0.66	0.66	0.61	0.63	0.64	0.64
		(0.02)	(0.03)	(0.03)	(0.04)	(0.04)	(0.02)	(0.03)	(0.02)	(0.03)	(0.02)	(0.02)	(0.02)	(0.02)	(0.02)	(0.02)	(0.02)	(0.02)	(0.02)	(0.02)	(0.02)	(0.02)	(0.03)	(0.02)	(0.02)	(0.02)	(0.02)	(0.02)	(0.02)	(0.02)	(0.03)	(0.03)	(0.02)	(0.02)
	AUC	0.57	0.61	0.67	0.68	0.69	0.61	0.66	0.67	0.68	0.63	0.64	0.66	0.66	0.64	0.64	0.65	0.65	0.72	0.71	0.72	0.72	0.64	0.66	0.67	0.67	0.72	0.71	0.72	0.72	0.65	0.69	0.69	0.69
		(0.03)	(0.03)	(0.04)	(0.04)	(0.04)	(0.03)	(0.03)	(0.03)	(0.03)	(0.03)	(0.03)	(0.03)	(0.03)	(0.02)	(0.03)	(0.03)	(0.03)	(0.02)	(0.02)	(0.02)	(0.02)	(0.03)	(0.03)	(0.03)	(0.03)	(0.03)	(0.03)	(0.03)	(0.03)	(0.03)	(0.03)	(0.03)	(0.03)
300	PA	0.58	0.6	0.65	0.66	0.66	0.61	0.64	0.65	0.65	0.59	0.6	0.61	0.61	0.62	0.62	0.63	0.63	0.67	0.66	0.67	0.67	0.62	0.62	0.63	0.63	0.68	0.67	0.67	0.67	0.62	0.64	0.65	0.65
		(0.02)	(0.02)	(0.02)	(0.02)	(0.02)	(0.02)	(0.02)	(0.02)	(0.02)	(0.02)	(0.02)	(0.02)	(0.02)	(0.03)	(0.02)	(0.02)	(0.01)	(0.02)	(0.02)	(0.02)	(0.02)	(0.02)	(0.02)	(0.02)	(0.02)	(0.02)	(0.02)	(0.02)	(0.02)	(0.02)	(0.02)	(0.02)	(0.02)
	PA_1	0.59	0.6	0.65	0.66	0.66	0.61	0.62	0.63	0.65	0.59	0.6	0.6	0.61	0.6	0.62	0.62	0.63	0.68	0.67	0.68	0.67	0.62	0.62	0.63	0.63	0.67	0.67	0.67	0.67	0.62	0.65	0.65	0.65
		(0.05)	(0.04)	(0.04)	(0.03)	(0.03)	(0.04)	(0.05)	(0.05)	(0.05)	(0.05)	(0.04)	(0.04)	(0.03)	(0.06)	(0.03)	(0.03)	(0.03)	(0.04)	(0.05)	(0.04)	(0.03)	(0.06)	(0.04)	(0.04)	(0.03)	(0.03)	(0.03)	(0.03)	(0.03)	(0.03)	(0.03)	(0.03)	(0.03)
	PA_2	0.58	0.6	0.65	0.66	0.66	0.61	0.65	0.66	0.65	0.6	0.6	0.61	0.62	0.64	0.62	0.63	0.63	0.67	0.66	0.67	0.67	0.61	0.63	0.63	0.63	0.68	0.67	0.68	0.67	0.61	0.64	0.64	0.64
		(0.05)	(0.04)	(0.04)	(0.03)	(0.03)	(0.04)	(0.04)	(0.05)	(0.05)	(0.05)	(0.04)	(0.04)	(0.04)	(0.06)	(0.03)	(0.02)	(0.03)	(0.04)	(0.04)	(0.03)	(0.03)	(0.06)	(0.03)	(0.03)	(0.03)	(0.03)	(0.03)	(0.03)	(0.03)	(0.04)	(0.03)	(0.04)	(0.04)
	g-means	0.58	0.6	0.65	0.66	0.66	0.61	0.64	0.64	0.65	0.59	0.6	0.61	0.61	0.62	0.62	0.63	0.63	0.67	0.66	0.67	0.67	0.62	0.62	0.63	0.63	0.67	0.67	0.67	0.67	0.62	0.64	0.65	0.65
		(0.02)	(0.02)	(0.02)	(0.02)	(0.02)	(0.02)	(0.02)	(0.02)	(0.02)	(0.02)	(0.02)	(0.02)	(0.02)	(0.03)	(0.02)	(0.02)	(0.01)	(0.02)	(0.02)	(0.02)	(0.02)	(0.02)	(0.02)	(0.02)	(0.02)	(0.02)	(0.02)	(0.02)	(0.02)	(0.02)	(0.02)	(0.02)	(0.02)
	AUC	0.58	0.64	0.7	0.72	0.72	0.65	0.69	0.7	0.71	0.64	0.64	0.65	0.66	0.67	0.67	0.68	0.68	0.74	0.73	0.74	0.74	0.66	0.67	0.68	0.68	0.74	0.73	0.74	0.73	0.66	0.7	0.7	0.7
		(0.03)	(0.02)	(0.02)	(0.02)	(0.02)	(0.03)	(0.03)	(0.03)	(0.03)	(0.03)	(0.03)	(0.03)	(0.03)	(0.03)	(0.02)	(0.02)	(0.02)	(0.02)	(0.02)	(0.02)	(0.02)	(0.03)	(0.02)	(0.02)	(0.02)	(0.02)	(0.02)	(0.02)	(0.02)	(0.02)	(0.02)	(0.02)	(0.02)
500	PA	0.6	0.6	0.66	0.67	0.67	0.62	0.65	0.66	0.66	0.61	0.6	0.6	0.61	0.64	0.64	0.64	0.64	0.68	0.67	0.69	0.69	0.63	0.63	0.64	0.64	0.68	0.68	0.68	0.68	0.62	0.65	0.65	0.66
		(0.02)	(0.02)	(0.02)	(0.02)	(0.02)	(0.02)	(0.02)	(0.02)	(0.02)	(0.02)	(0.02)	(0.02)	(0.02)	(0.02)	(0.02)	(0.02)	(0.02)	(0.02)	(0.02)	(0.02)	(0.02)	(0.02)	(0.02)	(0.02)	(0.02)	(0.02)	(0.02)	(0.02)	(0.02)	(0.02)	(0.02)	(0.02)	(0.02)
	PA_1	0.6	0.61	0.66	0.67	0.67	0.63	0.66	0.66	0.66	0.61	0.6	0.6	0.61	0.64	0.63	0.63	0.63	0.68	0.67	0.69	0.69	0.63	0.63	0.64	0.64	0.69	0.68	0.69	0.68	0.62	0.65	0.65	0.65
		(0.05)	(0.03)	(0.02)	(0.03)	(0.03)	(0.04)	(0.04)	(0.04)	(0.04)	(0.05)	(0.03)	(0.03)	(0.03)	(0.06)	(0.02)	(0.02)	(0.03)	(0.03)	(0.03)	(0.03)	(0.03)	(0.06)	(0.03)	(0.03)	(0.03)	(0.03)	(0.03)	(0.03)	(0.03)	(0.03)	(0.03)	(0.03)	(0.03)
	PA_2	0.59	0.6	0.65	0.67	0.67	0.61	0.65	0.66	0.67	0.61	0.6	0.6	0.61	0.65	0.64	0.64	0.64	0.68	0.67	0.69	0.69	0.63	0.63	0.64	0.64	0.68	0.68	0.68	0.68	0.63	0.65	0.65	0.66
		(0.05)	(0.03)	(0.03)	(0.03)	(0.03)	(0.04)	(0.03)	(0.03)	(0.03)	(0.05)	(0.03)	(0.03)	(0.03)	(0.05)	(0.04)	(0.04)	(0.03)	(0.03)	(0.03)	(0.03)	(0.03)	(0.06)	(0.03)	(0.03)	(0.03)	(0.03)	(0.03)	(0.03)	(0.03)	(0.03)	(0.03)	(0.03)	(0.03)
	g-means	0.59	0.6	0.66	0.67	0.67	0.62	0.65	0.66	0.66	0.6	0.6	0.6	0.61	0.64	0.64	0.64	0.64	0.68	0.67	0.69	0.69	0.63	0.63	0.64	0.64	0.68	0.68	0.68	0.68	0.62	0.65	0.65	0.66
		(0.02)	(0.02)	(0.02)	(0.02)	(0.02)	(0.02)	(0.02)	(0.02)	(0.02)	(0.02)	(0.02)	(0.02)	(0.02)	(0.02)	(0.02)	(0.02)	(0.02)	(0.02)	(0.02)	(0.02)	(0.02)	(0.02)	(0.02)	(0.02)	(0.02)	(0.02)	(0.02)	(0.02)	(0.02)	(0.02)	(0.02)	(0.02)	(0.02)
	AUC	0.6	0.64	0.71	0.73	0.74	0.67	0.71	0.72	0.72	0.65	0.64	0.65	0.65	0.7	0.69	0.69	0.69	0.75	0.74	0.75	0.75	0.68	0.68	0.69	0.69	0.75	0.75	0.75	0.75	0.67	0.7	0.71	0.71
		(0.03)	(0.02)	(0.02)	(0.02)	(0.02)	(0.03)	(0.02)	(0.02)	(0.02)	(0.02)	(0.02)	(0.02)	(0.02)	(0.02)	(0.03)	(0.02)	(0.02)	(0.02)	(0.02)	(0.02)	(0.02)	(0.02)	(0.02)	(0.02)	(0.02)	(0.02)	(0.02)	(0.02)	(0.02)	(0.02)	(0.02)	(0.02)	(0.02)
1000	PA	0.63	0.61	0.66	0.67	0.68	0.64	0.67	0.67	0.67	0.62	0.6	0.6	0.6	0.65	0.65	0.65	0.65	0.7	0.68	0.7	0.7	0.65	0.64	0.65	0.64	0.7	0.69	0.7	0.7	0.64	0.65	0.66	0.66
		(0.02)	(0.02)	(0.01)	(0.01)	(0.01)	(0.02)	(0.02)	(0.02)	(0.02)	(0.02)	(0.02)	(0.02)	(0.02)	(0.01)	(0.01)	(0.01)	(0.01)	(0.01)	(0.02)	(0.01)	(0.01)	(0.02)	(0.02)	(0.02)	(0.02)	(0.01)	(0.02)	(0.01)	(0.01)	(0.02)	(0.02)	(0.01)	(0.01)
	PA_1	0.63	0.62	0.67	0.67	0.67	0.64	0.66	0.67	0.67	0.62	0.6	0.6	0.59	0.66	0.65	0.65	0.65	0.7	0.68	0.7	0.7	0.65	0.65	0.65	0.65	0.7	0.69	0.7	0.7	0.64	0.66	0.66	0.66
		(0.04)	(0.03)	(0.02)	(0.02)	(0.02)	(0.04)	(0.04)	(0.03)	(0.03)	(0.04)	(0.03)	(0.03)	(0.03)	(0.05)	(0.03)	(0.03)	(0.02)	(0.02)	(0.03)	(0.02)	(0.02)	(0.05)	(0.03)	(0.03)	(0.03)	(0.02)	(0.02)	(0.02)	(0.02)	(0.02)	(0.03)	(0.03)	(0.03)
	PA_2	0.63	0.61	0.66	0.67	0.68	0.65	0.68	0.67	0.67	0.63	0.6	0.61	0.61	0.65	0.64	0.64	0.65	0.69	0.68	0.69	0.69	0.65	0.64	0.65	0.64	0.7	0.69	0.7	0.69	0.64	0.65	0.65	0.66
		(0.04)	(0.03)	(0.02)	(0.02)	(0.02)	(0.04)	(0.04)	(0.04)	(0.04)	(0.04)	(0.03)	(0.03)	(0.03)	(0.05)	(0.03)	(0.02)	(0.02)	(0.03)	(0.03)	(0.03)	(0.03)	(0.05)	(0.03)	(0.03)	(0.03)	(0.03)	(0.03)	(0.03)	(0.03)	(0.03)	(0.02)	(0.02)	(0.02)
	g-means	0.63	0.61	0.66	0.67	0.67	0.64	0.67	0.67	0.67	0.62	0.6	0.6	0.6	0.65	0.64	0.65	0.65	0.7	0.68	0.69	0.7	0.65	0.64	0.65	0.64	0.7	0.69	0.7	0.7	0.64	0.65	0.66	0.66
		(0.02)	(0.02)	(0.01)	(0.01)	(0.01)	(0.02)	(0.02)	(0.02)	(0.02)	(0.02)	(0.02)	(0.02)	(0.02)	(0.01)	(0.01)	(0.01)	(0.01)	(0.01)	(0.02)	(0.01)	(0.01)	(0.02)	(0.02)	(0.02)	(0.02)	(0.01)	(0.02)	(0.01)	(0.01)	(0.02)	(0.02)	(0.01)	(0.01)
	AUC	0.65	0.66	0.72	0.73	0.74	0.69	0.73	0.73	0.73	0.68	0.64	0.64	0.64	0.71	0.7	0.7	0.7	0.77	0.75	0.76	0.77	0.71	0.7	0.7	0.7	0.77	0.76	0.77	0.76	0.69	0.71	0.72	0.72
		(0.02)	(0.02)	(0.02)	(0.02)	(0.02)	(0.02)	(0.02)	(0.02)	(0.02)	(0.02)	(0.02)	(0.02)	(0.02)	(0.02)	(0.02)	(0.02)	(0.02)	(0.01)	(0.02)	(0.01)	(0.01)	(0.02)	(0.02)	(0.02)	(0.02)	(0.02)	(0.02)	(0.02)	(0.02)	(0.02)	(0.02)	(0.02)	(0.02)

Table 4. Performance of the classifiers for a moderate difference between the classes and a large number of variables ($\mu_2 = 1$, $p = 2,500$). The table reports predictive accuracy (PA), predictive accuracy for class 1 and class 2 (PA_1, PA_2), g-means and AUC. Training sets contained 2,500 variables; see the main text for details.

n_{train}		CART(5)	AdaBoost.M1(5).10	AdaBoost.M1(5).100	AdaBoost.M1(5).200	AdaBoost.M1(5).300	AdaBoost.M1.ICV(5).10	AdaBoost.M1.ICV(5).100	AdaBoost.M1.ICV(5).200	AdaBoost.M1.ICV(5).300	LogitBoost(1).10	LogitBoost(1).100	LogitBoost(1).200	LogitBoost(1).300	AdaBoost.M1(1).10	AdaBoost.M1(1).100	AdaBoost.M1(1).200	AdaBoost.M1(1).300	St-GrBoost(1).opt	St-GrBoost(1).100	St-GrBoost(1).300	St-GrBoost(1).500	GrBoost(1).10	GrBoost(1).100	GrBoost(1).200	GrBoost(1).300	St-GrBoost(5).opt	St-GrBoost(5).100	St-GrBoost(5).300	St-GrBoost(5).500	GrBoost(5).10	GrBoost(5).100	GrBoost(5).200	GrBoost(5).300
50	PA	0.69	0.68	0.68	0.68	0.68	0.71	0.76	0.77	0.78	0.71	0.74	0.74	0.74	0.72	0.76	0.77	0.77	0.78	0.77	0.78	0.78	0.72	0.75	0.76	0.76	0.79	0.78	0.79	0.79	0.68	0.67	0.67	0.67
		(0.04)	(0.05)	(0.05)	(0.05)	(0.05)	(0.04)	(0.03)	(0.03)	(0.02)	(0.04)	(0.03)	(0.03)	(0.03)	(0.06)	(0.04)	(0.04)	(0.04)	(0.04)	(0.04)	(0.04)	(0.04)	(0.04)	(0.04)	(0.04)	(0.04)	(0.04)	(0.03)	(0.03)	(0.03)	(0.05)	(0.05)	(0.05)	(0.05)
	PA_1	0.67	0.67	0.67	0.67	0.67	0.71	0.75	0.76	0.77	0.7	0.73	0.72	0.72	0.71	0.76	0.77	0.77	0.78	0.77	0.78	0.78	0.73	0.75	0.76	0.76	0.79	0.79	0.8	0.79	0.68	0.67	0.67	0.67
		(0.08)	(0.11)	(0.11)	(0.11)	(0.11)	(0.08)	(0.07)	(0.06)	(0.06)	(0.06)	(0.06)	(0.06)	(0.06)	(0.1)	(0.08)	(0.07)	(0.07)	(0.06)	(0.07)	(0.06)	(0.06)	(0.07)	(0.06)	(0.06)	(0.06)	(0.06)	(0.06)	(0.06)	(0.06)	(0.08)	(0.08)	(0.08)	(0.08)
	PA_2	0.7	0.69	0.69	0.69	0.69	0.72	0.77	0.78	0.78	0.72	0.75	0.75	0.75	0.73	0.76	0.77	0.76	0.77	0.76	0.77	0.78	0.71	0.75	0.76	0.76	0.79	0.78	0.79	0.79	0.67	0.66	0.66	0.66
		(0.07)	(0.08)	(0.08)	(0.08)	(0.08)	(0.07)	(0.07)	(0.07)	(0.06)	(0.06)	(0.06)	(0.06)	(0.06)	(0.05)	(0.04)	(0.04)	(0.05)	(0.07)	(0.07)	(0.07)	(0.07)	(0.09)	(0.06)	(0.06)	(0.06)	(0.06)	(0.06)	(0.06)	(0.06)	(0.08)	(0.09)	(0.09)	(0.09)
	g-means	0.68	0.67	0.67	0.67	0.67	0.71	0.76	0.77	0.77	0.71	0.74	0.73	0.73	0.72	0.76	0.77	0.77	0.77	0.76	0.77	0.77	0.72	0.75	0.75	0.76	0.79	0.78	0.79	0.79	0.68	0.66	0.66	0.66
		(0.04)	(0.05)	(0.05)	(0.05)	(0.05)	(0.04)	(0.03)	(0.03)	(0.03)	(0.04)	(0.03)	(0.04)	(0.04)	(0.06)	(0.04)	(0.04)	(0.04)	(0.04)	(0.04)	(0.04)	(0.04)	(0.04)	(0.04)	(0.04)	(0.04)	(0.04)	(0.03)	(0.03)	(0.04)	(0.05)	(0.05)	(0.05)	(0.05)
	AUC	0.69	0.68	0.68	0.68	0.68	0.79	0.84	0.86	0.86	0.78	0.81	0.79	0.79	0.8	0.85	0.85	0.85	0.86	0.86	0.86	0.86	0.79	0.83	0.83	0.82	0.88	0.87	0.88	0.88	0.74	0.7	0.7	0.7
		(0.04)	(0.05)	(0.05)	(0.05)	(0.05)	(0.04)	(0.04)	(0.03)	(0.03)	(0.04)	(0.04)	(0.05)	(0.05)	(0.06)	(0.04)	(0.04)	(0.04)	(0.04)	(0.03)	(0.03)	(0.03)	(0.05)	(0.04)	(0.05)	(0.04)	(0.03)	(0.03)	(0.03)	(0.03)	(0.05)	(0.05)	(0.04)	(0.04)
100	PA	0.7	0.7	0.7	0.7	0.7	0.75	0.79	0.8	0.81	0.74	0.78	0.78	0.78	0.76	0.79	0.8	0.8	0.83	0.81	0.82	0.83	0.76	0.79	0.8	0.8	0.82	0.81	0.82	0.82	0.77	0.79	0.79	0.79
		(0.04)	(0.03)	(0.03)	(0.03)	(0.03)	(0.03)	(0.02)	(0.02)	(0.02)	(0.03)	(0.02)	(0.02)	(0.02)	(0.02)	(0.02)	(0.02)	(0.02)	(0.02)	(0.03)	(0.02)	(0.02)	(0.03)	(0.02)	(0.02)	(0.02)	(0.02)	(0.03)	(0.02)	(0.03)	(0.03)	(0.04)	(0.04)	(0.04)
	PA_1	0.7	0.68	0.68	0.68	0.68	0.74	0.8	0.81	0.81	0.73	0.78	0.77	0.77	0.75	0.78	0.79	0.8	0.82	0.8	0.82	0.82	0.76	0.79	0.8	0.8	0.82	0.81	0.82	0.82	0.77	0.79	0.79	0.79
		(0.06)	(0.06)	(0.06)	(0.06)	(0.06)	(0.05)	(0.04)	(0.03)	(0.04)	(0.06)	(0.04)	(0.04)	(0.04)	(0.04)	(0.03)	(0.04)	(0.03)	(0.04)	(0.04)	(0.04)	(0.03)	(0.07)	(0.04)	(0.04)	(0.04)	(0.04)	(0.04)	(0.04)	(0.04)	(0.05)	(0.05)	(0.05)	(0.05)
	PA_2	0.7	0.72	0.72	0.72	0.72	0.76	0.79	0.8	0.81	0.75	0.79	0.79	0.79	0.76	0.8	0.81	0.81	0.83	0.81	0.83	0.83	0.77	0.8	0.8	0.8	0.82	0.81	0.82	0.82	0.76	0.79	0.79	0.8
		(0.06)	(0.06)	(0.06)	(0.06)	(0.06)	(0.05)	(0.04)	(0.04)	(0.04)	(0.05)	(0.04)	(0.04)	(0.04)	(0.04)	(0.03)	(0.03)	(0.03)	(0.04)	(0.05)	(0.04)	(0.04)	(0.07)	(0.04)	(0.04)	(0.04)	(0.04)	(0.04)	(0.04)	(0.04)	(0.05)	(0.05)	(0.05)	(0.05)
	g-means	0.7	0.7	0.7	0.7	0.7	0.75	0.79	0.8	0.81	0.74	0.78	0.78	0.78	0.76	0.79	0.8	0.8	0.82	0.81	0.82	0.83	0.76	0.79	0.8	0.8	0.82	0.81	0.82	0.82	0.76	0.79	0.79	0.79
		(0.04)	(0.04)	(0.04)	(0.04)	(0.04)	(0.03)	(0.02)	(0.02)	(0.02)	(0.03)	(0.02)	(0.02)	(0.02)	(0.02)	(0.02)	(0.02)	(0.02)	(0.02)	(0.03)	(0.02)	(0.02)	(0.03)	(0.02)	(0.02)	(0.02)	(0.02)	(0.03)	(0.02)	(0.03)	(0.03)	(0.04)	(0.04)	(0.04)
	AUC	0.7	0.71	0.71	0.71	0.71	0.82	0.87	0.88	0.89	0.82	0.87	0.85	0.85	0.84	0.88	0.88	0.88	0.91	0.89	0.91	0.91	0.84	0.88	0.88	0.88	0.91	0.9	0.91	0.91	0.84	0.86	0.85	0.85
		(0.04)	(0.04)	(0.04)	(0.04)	(0.04)	(0.03)	(0.02)	(0.02)	(0.02)	(0.03)	(0.02)	(0.03)	(0.03)	(0.03)	(0.02)	(0.02)	(0.02)	(0.02)	(0.02)	(0.02)	(0.02)	(0.03)	(0.02)	(0.02)	(0.02)	(0.02)	(0.02)	(0.02)	(0.02)	(0.04)	(0.04)	(0.04)	(0.04)
200	PA	0.71	0.72	0.72	0.72	0.72	0.78	0.81	0.82	0.82	0.76	0.8	0.8	0.8	0.79	0.81	0.82	0.82	0.84	0.82	0.84	0.84	0.79	0.81	0.82	0.82	0.84	0.83	0.84	0.84	0.8	0.82	0.83	0.83
		(0.03)	(0.04)	(0.04)	(0.04)	(0.04)	(0.02)	(0.02)	(0.02)	(0.02)	(0.02)	(0.02)	(0.02)	(0.02)	(0.02)	(0.02)	(0.02)	(0.02)	(0.01)	(0.02)	(0.01)	(0.01)	(0.02)	(0.02)	(0.01)	(0.01)	(0.01)	(0.02)	(0.01)	(0.01)	(0.02)	(0.02)	(0.02)	(0.02)
	PA_1	0.71	0.71	0.7	0.7	0.7	0.77	0.79	0.8	0.81	0.76	0.79	0.79	0.79	0.77	0.8	0.81	0.81	0.84	0.82	0.84	0.84	0.79	0.82	0.82	0.82	0.84	0.83	0.84	0.84	0.8	0.82	0.83	0.83
		(0.05)	(0.05)	(0.05)	(0.05)	(0.05)	(0.04)	(0.05)	(0.04)	(0.04)	(0.04)	(0.03)	(0.03)	(0.03)	(0.05)	(0.04)	(0.03)	(0.04)	(0.02)	(0.04)	(0.03)	(0.02)	(0.05)	(0.03)	(0.03)	(0.03)	(0.02)	(0.03)	(0.02)	(0.02)	(0.04)	(0.03)	(0.03)	(0.03)
	PA_2	0.72	0.74	0.74	0.74	0.74	0.79	0.82	0.83	0.83	0.76	0.8	0.8	0.8	0.8	0.82	0.82	0.82	0.84	0.82	0.84	0.84	0.78	0.81	0.82	0.82	0.84	0.82	0.84	0.84	0.79	0.82	0.83	0.83
		(0.04)	(0.06)	(0.06)	(0.06)	(0.06)	(0.04)	(0.04)	(0.03)	(0.03)	(0.04)	(0.03)	(0.03)	(0.03)	(0.04)	(0.02)	(0.02)	(0.02)	(0.03)	(0.03)	(0.03)	(0.03)	(0.05)	(0.03)	(0.03)	(0.03)	(0.03)	(0.03)	(0.03)	(0.03)	(0.03)	(0.03)	(0.03)	(0.03)
	g-means	0.71	0.72	0.72	0.72	0.72	0.78	0.81	0.82	0.82	0.76	0.8	0.8	0.8	0.79	0.81	0.82	0.82	0.84	0.82	0.84	0.84	0.79	0.81	0.82	0.82	0.84	0.83	0.84	0.84	0.79	0.82	0.83	0.83
		(0.03)	(0.04)	(0.04)	(0.04)	(0.04)	(0.02)	(0.02)	(0.02)	(0.02)	(0.02)	(0.02)	(0.02)	(0.02)	(0.02)	(0.02)	(0.02)	(0.02)	(0.01)	(0.02)	(0.01)	(0.01)	(0.02)	(0.02)	(0.01)	(0.01)	(0.01)	(0.02)	(0.01)	(0.01)	(0.02)	(0.02)	(0.02)	(0.02)
	AUC	0.71	0.79	0.79	0.79	0.79	0.86	0.89	0.9	0.9	0.84	0.88	0.88	0.88	0.87	0.89	0.9	0.9	0.92	0.9	0.92	0.92	0.87	0.9	0.9	0.9	0.92	0.91	0.92	0.92	0.88	0.9	0.9	0.89
		(0.03)	(0.06)	(0.06)	(0.06)	(0.06)	(0.02)	(0.02)	(0.02)	(0.02)	(0.02)	(0.02)	(0.02)	(0.02)	(0.02)	(0.02)	(0.02)	(0.02)	(0.01)	(0.02)	(0.01)	(0.01)	(0.02)	(0.01)	(0.01)	(0.01)	(0.01)	(0.01)	(0.01)	(0.01)	(0.02)	(0.01)	(0.01)	(0.02)
300	PA	0.73	0.8	0.84	0.84	0.84	0.79	0.82	0.83	0.83	0.77	0.8	0.8	0.8	0.79	0.81	0.81	0.81	0.84	0.82	0.84	0.84	0.79	0.82	0.82	0.82	0.85	0.84	0.85	0.85	0.81	0.83	0.83	0.84
		(0.02)	(0.02)	(0.01)	(0.01)	(0.01)	(0.02)	(0.02)	(0.02)	(0.02)	(0.02)	(0.02)	(0.02)	(0.02)	(0.02)	(0.01)	(0.01)	(0.01)	(0.01)	(0.02)	(0.01)	(0.01)	(0.02)	(0.01)	(0.02)	(0.02)	(0.01)	(0.02)	(0.01)	(0.01)	(0.01)	(0.01)	(0.01)	(0.01)
	PA_1	0.73	0.79	0.84	0.84	0.84	0.78	0.82	0.83	0.83	0.77	0.8	0.8	0.8	0.8	0.81	0.81	0.81	0.84	0.81	0.84	0.84	0.79	0.81	0.82	0.82	0.85	0.84	0.85	0.85	0.81	0.83	0.83	0.84
		(0.04)	(0.02)	(0.02)	(0.02)	(0.02)	(0.03)	(0.03)	(0.02)	(0.02)	(0.03)	(0.03)	(0.03)	(0.03)	(0.03)	(0.02)	(0.03)	(0.03)	(0.02)	(0.03)	(0.02)	(0.02)	(0.06)	(0.03)	(0.03)	(0.03)	(0.02)	(0.03)	(0.02)	(0.02)	(0.03)	(0.03)	(0.03)	(0.03)
	PA_2	0.73	0.8	0.83	0.84	0.84	0.79	0.82	0.82	0.82	0.77	0.8	0.81	0.81	0.78	0.81	0.81	0.81	0.85	0.82	0.84	0.85	0.79	0.82	0.82	0.82	0.85	0.84	0.84	0.85	0.81	0.83	0.84	0.84
		(0.04)	(0.02)	(0.02)	(0.02)	(0.02)	(0.04)	(0.02)	(0.03)	(0.03)	(0.04)	(0.03)	(0.03)	(0.03)	(0.03)	(0.03)	(0.02)	(0.03)	(0.02)	(0.03)	(0.02)	(0.02)	(0.05)	(0.03)	(0.03)	(0.03)	(0.02)	(0.03)	(0.02)	(0.02)	(0.02)	(0.02)	(0.02)	(0.02)
	g-means	0.73	0.8	0.84	0.84	0.84	0.79	0.82	0.83	0.83	0.77	0.8	0.8	0.8	0.79	0.81	0.81	0.81	0.84	0.82	0.84	0.84	0.79	0.82	0.82	0.82	0.85	0.84	0.85	0.85	0.81	0.83	0.83	0.84
		(0.02)	(0.02)	(0.01)	(0.01)	(0.01)	(0.02)	(0.02)	(0.02)	(0.02)	(0.02)	(0.02)	(0.02)	(0.02)	(0.02)	(0.01)	(0.01)	(0.01)	(0.01)	(0.02)	(0.01)	(0.01)	(0.02)	(0.02)	(0.02)	(0.02)	(0.01)	(0.02)	(0.01)	(0.01)	(0.01)	(0.01)	(0.01)	(0.01)
	AUC	0.72	0.88	0.92	0.92	0.92	0.86	0.9	0.91	0.91	0.85	0.88	0.89	0.88	0.87	0.89	0.89	0.9	0.92	0.9	0.92	0.92	0.87	0.9	0.9	0.9	0.93	0.92	0.93	0.93	0.89	0.91	0.91	0.9
		(0.03)	(0.02)	(0.01)	(0.01)	(0.01)	(0.02)	(0.01)	(0.01)	(0.01)	(0.02)	(0.02)	(0.01)	(0.01)	(0.02)	(0.01)	(0.01)	(0.01)	(0.01)	(0.02)	(0.01)	(0.01)	(0.02)	(0.01)	(0.01)	(0.01)	(0.01)	(0.01)	(0.01)	(0.01)	(0.01)	(0.01)	(0.01)	(0.01)
500	PA	0.76	0.8	0.85	0.85	0.85	0.81	0.84	0.84	0.84	0.79	0.79	0.81	0.81	0.81	0.82	0.82	0.83	0.85	0.82	0.85	0.85	0.8	0.82	0.82	0.82	0.86	0.85	0.85	0.86	0.81	0.83	0.84	0.84
		(0.02)	(0.02)	(0.01)	(0.01)	(0.01)	(0.02)	(0.01)	(0.01)	(0.01)	(0.02)	(0.02)	(0.02)	(0.02)	(0.01)	(0.01)	(0.01)	(0.01)	(0.01)	(0.02)	(0.01)	(0.01)	(0.02)	(0.01)	(0.01)	(0.01)	(0.01)	(0.01)	(0.01)	(0.01)	(0.01)	(0.01)	(0.01)	(0.01)
	PA_1	0.76	0.81	0.85	0.85	0.85	0.81	0.84	0.85	0.84	0.79	0.8	0.81	0.81	0.81	0.82	0.82	0.82	0.85	0.82	0.84	0.85	0.79	0.82	0.82	0.83	0.86	0.85	0.86	0.86	0.81	0.83	0.84	0.84
		(0.03)	(0.02)	(0.02)	(0.02)	(0.02)	(0.02)	(0.02)	(0.02)	(0.02)	(0.03)	(0.02)	(0.02)	(0.02)	(0.03)	(0.02)	(0.02)	(0.02)	(0.02)	(0.03)	(0.02)	(0.02)	(0.04)	(0.02)	(0.02)	(0.02)	(0.02)	(0.02)	(0.02)	(0.02)	(0.02)	(0.02)	(0.02)	(0.02)
	PA_2	0.75	0.8	0.84	0.85	0.85	0.81	0.83	0.84	0.84	0.79	0.79	0.81	0.81	0.81	0.82	0.82	0.83	0.85	0.83	0.85	0.85	0.8	0.82	0.82	0.82	0.86	0.85	0.85	0.86	0.81	0.83	0.84	0.84
		(0.03)	(0.03)	(0.02)	(0.02)	(0.02)	(0.02)	(0.03)	(0.03)	(0.03)	(0.03)	(0.03)	(0.02)	(0.02)	(0.04)	(0.02)	(0.02)	(0.02)	(0.02)	(0.03)	(0.02)	(0.02)	(0.04)	(0.02)	(0.02)	(0.02)	(0.02)	(0.02)	(0.02)	(0.02)	(0.02)	(0.02)	(0.02)	(0.02)
	g-means	0.76	0.8	0.85	0.85	0.85	0.81	0.84	0.84	0.84	0.79	0.79	0.81	0.81	0.81	0.82	0.82	0.83	0.85	0.82	0.85	0.85	0.8	0.82	0.82	0.82	0.86	0.85	0.85	0.86	0.81	0.83	0.84	0.84
		(0.02)	(0.02)	(0.01)	(0.01)	(0.01)	(0.02)	(0.01)	(0.01)	(0.01)	(0.02)	(0.02)	(0.02)	(0.02)	(0.01)	(0.01)	(0.01)	(0.01)	(0.01)	(0.02)	(0.01)	(0.01)	(0.02)	(0.01)	(0.01)	(0.01)	(0.01)	(0.01)	(0.01)	(0.01)	(0.01)	(0.01)	(0.01)	(0.01)
	AUC	0.76	0.88	0.92	0.93	0.93	0.89	0.92	0.92	0.92	0.87	0.88	0.89	0.89	0.89	0.9	0.9	0.91	0.93	0.91	0.92	0.93	0.88	0.9	0.9	0.91	0.93	0.93	0.93	0.93	0.89	0.91	0.91	0.91
		(0.02)	(0.01)	(0.01)	(0.01)	(0.01)	(0.02)	(0.01)	(0.01)	(0.01)	(0.02)	(0.02)	(0.01)	(0.01)	(0.01)	(0.01)	(0.01)	(0.01)	(0.01)	(0.01)	(0.01)	(0.01)	(0.01)	(0.01)	(0.01)	(0.01)	(0.01)	(0.01)	(0.01)	(0.01)	(0.01)	(0.01)	(0.01)	(0.01)
1000	PA	0.78	0.81	0.85	0.85	0.85	0.82	0.84	0.85	0.85	0.8	0.79	0.79	0.8	0.81	0.83	0.83	0.83	0.85	0.83	0.84	0.85	0.81	0.83	0.83	0.83	0.86	0.85	0.86	0.86	0.82	0.84	0.84	0.84
		(0.01)	(0.01)	(0.02)	(0.01)	(0.01)	(0.02)	(0.01)	(0.02)	(0.02)	(0.02)	(0.01)	(0.01)	(0.02)	(0.01)	(0.01)	(0.01)	(0.01)	(0.01)	(0.02)	(0.01)	(0.01)	(0.01)	(0.01)	(0.01)	(0.01)	(0.01)	(0.01)	(0.01)	(0.01)	(0.01)	(0.01)	(0.01)	(0.01)
	PA_1	0.78	0.82	0.85	0.86	0.86	0.82	0.85	0.85	0.85	0.79	0.79	0.79	0.8	0.8	0.82	0.83	0.83	0.85	0.83	0.85	0.85	0.81	0.82	0.83	0.83	0.85	0.85	0.85	0.85	0.82	0.84	0.84	0.84
		(0.03)	(0.02)	(0.02)	(0.01)	(0.01)	(0.03)	(0.02)	(0.02)	(0.01)	(0.03)	(0.02)	(0.02)	(0.02)	(0.04)	(0.02)	(0.02)	(0.02)	(0.02)	(0.03)	(0.02)	(0.02)	(0.04)	(0.02)	(0.02)	(0.02)	(0.02)	(0.02)	(0.02)	(0.02)	(0.02)	(0.02)	(0.02)	(0.02)
	PA_2	0.78	0.8	0.85	0.85	0.85	0.82	0.84	0.84	0.84	0.8	0.78	0.79	0.8	0.81	0.83	0.83	0.83	0.85	0.83	0.84	0.85	0.81	0.83	0.83	0.83	0.86	0.85	0.86	0.86	0.82	0.84	0.84	0.85
		(0.03)	(0.03)	(0.02)	(0.02)	(0.02)	(0.02)	(0.03)	(0.03)	(0.03)	(0.03)	(0.02)	(0.02)	(0.02)	(0.03)	(0.02)	(0.01)	(0.02)	(0.02)	(0.02)	(0.02)	(0.02)	(0.04)	(0.02)	(0.02)	(0.02)	(0.02)	(0.02)	(0.02)	(0.02)	(0.02)	(0.02)	(0.02)	(0.02)
	g-means	0.78	0.81	0.85	0.85	0.85	0.82	0.84	0.85	0.85	0.8	0.79	0.79	0.8	0.81	0.83	0.83	0.83	0.85	0.83	0.84	0.85	0.81	0.83	0.83	0.83	0.86	0.85	0.86	0.86	0.82	0.84	0.84	0.84
		(0.01)	(0.01)	(0.02)	(0.01)	(0.01)	(0.02)	(0.01)	(0.02)	(0.02)	(0.02)	(0.01)	(0.01)	(0.02)	(0.01)	(0.01)	(0.01)	(0.01)	(0.01)	(0.02)	(0.01)	(0.01)	(0.02)	(0.01)	(0.01)	(0.01)	(0.01)	(0.01)	(0.01)	(0.01)	(0.01)	(0.01)	(0.01)	(0.01)
	AUC	0.82	0.89	0.93	0.93	0.93	0.9	0.92	0.92	0.92	0.88	0.87	0.88	0.88	0.89	0.91	0.91	0.91	0.93	0.91	0.92	0.93	0.89	0.91	0.91	0.91	0.93	0.93	0.93	0.93	0.9	0.92	0.92	0.92
		(0.02)	(0.01)	(0.01)	(0.01)	(0.01)	(0.01)	(0.01)	(0.01)	(0.01)	(0.01)	(0.01)	(0.01)	(0.01)	(0.01)	(0.01)	(0.01)	(0.01)	(0.01)	(0.01)	(0.01)	(0.01)	(0.01)	(0.01)	(0.01)	(0.01)	(0.01)	(0.01)	(0.01)	(0.01)	(0.01)	(0.01)	(0.01)	(0.01)
