# Supplementary material for: Boosting for high-dimensional two-class prediction
Source: BMC Bioinformatics. 2015 Sep 21;16:300. doi: 10.1186/s12859-015-0723-9 (PMC4578758; doi:10.1186/s12859-015-0723-9)
Supplement: Additional file 6 — Results obtained by reanalyzing real microarray datasets (1 table). The table in the Additional file reports the performance measures (predictive accuracy - PA, predictive accuracy for class 1 - PA1, predictive accuracy for class 2 - PA2, g-means and AUC) for different ensemble classifiers and datasets/prediction tasks; darker shading denotes better performance in terms of the relevant accuracy measure. (PDF 111 kb) [file 12859_2015_723_MOESM6_ESM.pdf]

Table 1. Performance measures (predictive accuracy - PA, predictive accuracy for class 1 - PA_1, predictive accuracy for class 2 - PA_2, g-means and AUC) for different boosting algorithms and datasets/classification tasks (the number behind the name of the classifier denotes the number of boosting iterations; see the Methods section for more details); darker shading denotes better performance in terms of the relevant accuracy measure.

Dataset	Measure	CART(5)	AdaBoost.M1(5).10	AdaBoost.M1(5).100	AdaBoost.M1(5).300	AdaBoost.M1.ICV(5).10	AdaBoost.M1.ICV(5).100	AdaBoost.M1.ICV(5).300	AdaBoost.M1(1).10	AdaBoost.M1(1).100	LogitBoost(1).10	LogitBoost(1).100	St-GrBoost(1).opt	St-GrBoost(1).100	St-GrBoost(1).300	St-GrBoost(1).500	GrBoost(1).10	GrBoost(1).100	GrBoost(1).200	GrBoost(1).300	St-GrBoost(5).opt	St-GrBoost(5).100	St-GrBoost(5).300	St-GrBoost(5).500	GrBoost(5).10	GrBoost(5).100	GrBoost(5).200	GrBoost(5).300
Ivshina ER	PA	0.74 (0.07)	0.75 (0.07)	0.76 (0.06)	0.75 (0.06)	0.75 (0.07)	0.8 (0.03)	0.83 (0.03)	0.78 (0.06)	0.83 (0.05)	0.79 (0.03)	0.8 (0.02)	0.86 (0.01)	0.86 (0.01)	0.86 (0.01)	0.86 (0.01)	0.8 (0.03)	0.82 (0.02)	0.82 (0.02)	0.82 (0.02)	0.86 (0.01)	0.86 (0.01)	0.86 (0.01)	0.86 (0.01)	0.76 (0.04)	0.73 (0.04)	0.73 (0.04)	0.73 (0.04)
	PA_1	0.74 (0.08)	0.74 (0.1)	0.75 (0.08)	0.74 (0.08)	0.75 (0.06)	0.82 (0.03)	0.82 (0.04)	0.78 (0.07)	0.83 (0.06)	0.78 (0.07)	0.85 (0.04)	0.87 (0.03)	0.87 (0.03)	0.88 (0.03)	0.87 (0.03)	0.81 (0.05)	0.83 (0.04)	0.83 (0.04)	0.83 (0.04)	0.88 (0.02)	0.88 (0.02)	0.88 (0.02)	0.88 (0.02)	0.75 (0.06)	0.73 (0.06)	0.73 (0.06)	0.73 (0.06)
	PA_2	0.75 (0.07)	0.77 (0.08)	0.76 (0.08)	0.75 (0.08)	0.76 (0.09)	0.78 (0.06)	0.84 (0.03)	0.78 (0.06)	0.82 (0.07)	0.8 (0.03)	0.79 (0.02)	0.85 (0.02)	0.85 (0.02)	0.85 (0.02)	0.85 (0.02)	0.8 (0.04)	0.82 (0.02)	0.82 (0.02)	0.82 (0.02)	0.86 (0.01)	0.85 (0.01)	0.86 (0.01)	0.86 (0.02)	0.76 (0.05)	0.73 (0.05)	0.73 (0.05)	0.73 (0.05)
	g-means	0.74 (0.07)	0.75 (0.07)	0.75 (0.06)	0.74 (0.06)	0.75 (0.07)	0.8 (0.03)	0.83 (0.03)	0.78 (0.06)	0.83 (0.05)	0.79 (0.04)	0.82 (0.02)	0.86 (0.02)	0.86 (0.02)	0.87 (0.02)	0.86 (0.02)	0.8 (0.03)	0.82 (0.02)	0.83 (0.02)	0.82 (0.02)	0.87 (0.01)	0.87 (0.01)	0.87 (0.01)	0.87 (0.01)	0.76 (0.04)	0.73 (0.04)	0.73 (0.04)	0.73 (0.04)
	AUC	0.74 (0.07)	0.75 (0.07)	0.76 (0.06)	0.75 (0.06)	0.81 (0.05)	0.86 (0.03)	0.87 (0.05)	0.83 (0.05)	0.86 (0.04)	0.79 (0.04)	0.82 (0.02)	0.88 (0.01)	0.89 (0.01)	0.89 (0.01)	0.89 (0.01)	0.85 (0.02)	0.87 (0.02)	0.86 (0.02)	0.86 (0.02)	0.89 (0.01)	0.89 (0.01)	0.89 (0.01)	0.89 (0.01)	0.8 (0.04)	0.77 (0.04)	0.77 (0.04)	0.77 (0.04)
Ivshina Grade	PA	0.73 (0.05)	0.73 (0.04)	0.73 (0.05)	0.72 (0.05)	0.78 (0.04)	0.79 (0.03)	0.8 (0.03)	0.77 (0.04)	0.8 (0.03)	0.75 (0.02)	0.8 (0.02)	0.83 (0.01)	0.82 (0.02)	0.82 (0.01)	0.83 (0.01)	0.78 (0.02)	0.81 (0.02)	0.81 (0.02)	0.81 (0.02)	0.83 (0.02)	0.82 (0.02)	0.83 (0.02)	0.82 (0.02)	0.78 (0.03)	0.8 (0.03)	0.79 (0.03)	0.79 (0.03)
	PA_1	0.72 (0.06)	0.73 (0.06)	0.72 (0.06)	0.72 (0.07)	0.77 (0.04)	0.79 (0.03)	0.8 (0.04)	0.78 (0.05)	0.81 (0.04)	0.75 (0.03)	0.79 (0.02)	0.85 (0.03)	0.84 (0.03)	0.85 (0.03)	0.85 (0.03)	0.79 (0.05)	0.82 (0.04)	0.83 (0.04)	0.83 (0.04)	0.86 (0.03)	0.85 (0.03)	0.86 (0.03)	0.86 (0.03)	0.79 (0.06)	0.81 (0.05)	0.8 (0.05)	0.8 (0.05)
	PA_2	0.73 (0.07)	0.73 (0.06)	0.74 (0.07)	0.73 (0.05)	0.79 (0.05)	0.79 (0.05)	0.79 (0.03)	0.76 (0.05)	0.79 (0.05)	0.76 (0.05)	0.81 (0.04)	0.82 (0.02)	0.81 (0.02)	0.82 (0.02)	0.82 (0.02)	0.78 (0.03)	0.8 (0.02)	0.81 (0.02)	0.81 (0.02)	0.82 (0.02)	0.82 (0.02)	0.82 (0.02)	0.82 (0.02)	0.78 (0.03)	0.79 (0.03)	0.79 (0.03)	0.79 (0.03)
	g-means	0.72 (0.05)	0.73 (0.04)	0.73 (0.05)	0.72 (0.05)	0.78 (0.04)	0.79 (0.03)	0.8 (0.03)	0.77 (0.04)	0.8 (0.04)	0.75 (0.03)	0.8 (0.02)	0.83 (0.02)	0.83 (0.02)	0.83 (0.01)	0.84 (0.02)	0.78 (0.03)	0.81 (0.02)	0.82 (0.02)	0.82 (0.02)	0.84 (0.01)	0.83 (0.02)	0.84 (0.02)	0.84 (0.02)	0.79 (0.03)	0.8 (0.03)	0.8 (0.03)	0.79 (0.03)
	AUC	0.73 (0.05)	0.73 (0.04)	0.73 (0.05)	0.72 (0.05)	0.83 (0.03)	0.84 (0.03)	0.85 (0.02)	0.83 (0.04)	0.85 (0.03)	0.75 (0.03)	0.8 (0.02)	0.88 (0.01)	0.88 (0.01)	0.88 (0.01)	0.88 (0.01)	0.84 (0.02)	0.87 (0.01)	0.87 (0.01)	0.87 (0.01)	0.89 (0.01)	0.89 (0.01)	0.89 (0.01)	0.89 (0.01)	0.83 (0.03)	0.83 (0.03)	0.82 (0.04)	0.81 (0.04)
Wang ER	PA	0.79 (0.03)	0.79 (0.03)	0.79 (0.04)	0.79 (0.03)	0.82 (0.03)	0.85 (0.03)	0.84 (0.01)	0.85 (0.02)	0.86 (0.03)	0.82 (0.02)	0.86 (0.01)	0.89 (0.01)	0.89 (0.01)	0.89 (0.01)	0.89 (0.01)	0.85 (0.01)	0.86 (0.02)	0.86 (0.02)	0.86 (0.02)	0.89 (0.01)	0.89 (0.01)	0.89 (0.01)	0.89 (0.01)	0.85 (0.02)	0.86 (0.02)	0.86 (0.02)	0.86 (0.02)
	PA_1	0.79 (0.04)	0.79 (0.04)	0.78 (0.04)	0.79 (0.05)	0.83 (0.04)	0.84 (0.04)	0.85 (0.02)	0.84 (0.03)	0.87 (0.03)	0.81 (0.03)	0.85 (0.03)	0.88 (0.01)	0.88 (0.02)	0.88 (0.01)	0.88 (0.01)	0.85 (0.03)	0.85 (0.02)	0.85 (0.02)	0.85 (0.02)	0.88 (0.02)	0.87 (0.02)	0.88 (0.02)	0.87 (0.02)	0.85 (0.03)	0.86 (0.02)	0.86 (0.02)	0.85 (0.02)
	PA_2	0.8 (0.04)	0.8 (0.04)	0.8 (0.05)	0.8 (0.05)	0.82 (0.04)	0.85 (0.03)	0.84 (0.02)	0.85 (0.03)	0.86 (0.03)	0.82 (0.03)	0.86 (0.01)	0.89 (0.01)	0.89 (0.01)	0.89 (0.01)	0.89 (0.01)	0.86 (0.02)	0.86 (0.02)	0.86 (0.02)	0.86 (0.02)	0.89 (0.01)	0.89 (0.01)	0.89 (0.01)	0.9 (0.01)	0.86 (0.02)	0.87 (0.02)	0.87 (0.02)	0.87 (0.02)
	g-means	0.79 (0.03)	0.79 (0.03)	0.79 (0.04)	0.79 (0.03)	0.82 (0.03)	0.85 (0.03)	0.84 (0.01)	0.85 (0.02)	0.86 (0.03)	0.81 (0.02)	0.86 (0.01)	0.89 (0.01)	0.89 (0.01)	0.89 (0.01)	0.89 (0.01)	0.85 (0.02)	0.85 (0.02)	0.85 (0.02)	0.85 (0.02)	0.89 (0.01)	0.88 (0.01)	0.89 (0.01)	0.88 (0.01)	0.85 (0.02)	0.86 (0.01)	0.86 (0.02)	0.86 (0.02)
	AUC	0.79 (0.03)	0.79 (0.03)	0.79 (0.04)	0.8 (0.03)	0.87 (0.02)	0.89 (0.02)	0.89 (0.01)	0.88 (0.02)	0.9 (0.02)	0.81 (0.02)	0.86 (0.01)	0.91 (0.01)	0.92 (0.01)	0.91 (0.01)	0.91 (0.01)	0.89 (0.01)	0.89 (0.01)	0.89 (0.01)	0.89 (0.01)	0.91 (0.01)	0.92 (0.01)	0.92 (0.01)	0.92 (0.01)	0.89 (0.02)	0.89 (0.02)	0.88 (0.02)	0.88 (0.02)
Wang Relapse	PA	0.54 (0.03)	0.58 (0.04)	0.62 (0.03)	0.63 (0.03)	0.56 (0.03)	0.58 (0.02)	0.61 (0.02)	0.58 (0.03)	0.62 (0.03)	0.57 (0.03)	0.6 (0.03)	0.6 (0.03)	0.61 (0.02)	0.62 (0.02)	0.63 (0.02)	0.57 (0.03)	0.6 (0.02)	0.62 (0.02)	0.62 (0.02)	0.62 (0.02)	0.62 (0.02)	0.63 (0.02)	0.64 (0.02)	0.59 (0.02)	0.62 (0.03)	0.62 (0.02)	0.63 (0.02)
	PA_1	0.54 (0.05)	0.57 (0.05)	0.62 (0.04)	0.62 (0.04)	0.57 (0.04)	0.58 (0.03)	0.6 (0.09)	0.58 (0.05)	0.61 (0.05)	0.57 (0.04)	0.61 (0.04)	0.61 (0.05)	0.61 (0.04)	0.63 (0.04)	0.64 (0.03)	0.57 (0.06)	0.59 (0.04)	0.61 (0.04)	0.61 (0.03)	0.63 (0.04)	0.63 (0.04)	0.64 (0.03)	0.64 (0.03)	0.58 (0.04)	0.61 (0.04)	0.63 (0.03)	0.63 (0.03)
	PA_2	0.54 (0.05)	0.58 (0.05)	0.62 (0.04)	0.64 (0.04)	0.55 (0.04)	0.59 (0.04)	0.61 (0.06)	0.58 (0.05)	0.63 (0.04)	0.56 (0.06)	0.6 (0.05)	0.6 (0.04)	0.6 (0.03)	0.62 (0.03)	0.62 (0.03)	0.58 (0.04)	0.61 (0.03)	0.62 (0.03)	0.63 (0.03)	0.62 (0.03)	0.62 (0.03)	0.63 (0.03)	0.63 (0.03)	0.6 (0.03)	0.62 (0.03)	0.62 (0.03)	0.63 (0.03)
	g-means	0.54 (0.03)	0.57 (0.04)	0.62 (0.03)	0.63 (0.03)	0.56 (0.03)	0.58 (0.02)	0.6 (0.02)	0.58 (0.03)	0.62 (0.03)	0.57 (0.03)	0.6 (0.03)	0.6 (0.03)	0.61 (0.02)	0.62 (0.02)	0.63 (0.02)	0.57 (0.03)	0.6 (0.03)	0.61 (0.02)	0.62 (0.02)	0.63 (0.02)	0.62 (0.02)	0.63 (0.02)	0.64 (0.02)	0.59 (0.03)	0.62 (0.03)	0.62 (0.02)	0.63 (0.02)
	AUC	0.54 (0.04)	0.61 (0.04)	0.66 (0.04)	0.68 (0.03)	0.58 (0.03)	0.6 (0.03)	0.64 (0.03)	0.61 (0.04)	0.66 (0.04)	0.57 (0.03)	0.6 (0.03)	0.63 (0.02)	0.64 (0.02)	0.67 (0.02)	0.68 (0.02)	0.6 (0.03)	0.64 (0.03)	0.66 (0.03)	0.66 (0.03)	0.66 (0.03)	0.67 (0.02)	0.68 (0.02)	0.69 (0.02)	0.62 (0.03)	0.66 (0.02)	0.67 (0.02)	0.67 (0.02)
Sotiriou ER	PA	0.72 (0.06)	0.71 (0.06)	0.71 (0.06)	0.71 (0.06)	0.7 (0.05)	0.78 (0.02)	0.78 (0.06)	0.78 (0.05)	0.82 (0.04)	0.79 (0.03)	0.81 (0.03)	0.85 (0.02)	0.84 (0.02)	0.85 (0.02)	0.85 (0.02)	0.78 (0.04)	0.82 (0.03)	0.83 (0.03)	0.83 (0.03)	0.86 (0.02)	0.85 (0.02)	0.86 (0.02)	0.86 (0.02)	0.73 (0.04)	0.72 (0.05)	0.72 (0.05)	0.72 (0.05)
	PA_1	0.72 (0.07)	0.71 (0.08)	0.71 (0.08)	0.71 (0.1)	0.64 (0.11)	0.78 (0.05)	0.74 (0.08)	0.78 (0.06)	0.8 (0.04)	0.75 (0.05)	0.84 (0.05)	0.81 (0.04)	0.81 (0.04)	0.81 (0.04)	0.81 (0.04)	0.77 (0.08)	0.79 (0.05)	0.8 (0.04)	0.8 (0.05)	0.82 (0.03)	0.82 (0.04)	0.82 (0.03)	0.82 (0.03)	0.73 (0.07)	0.72 (0.08)	0.72 (0.08)	0.72 (0.08)
	PA_2	0.72 (0.08)	0.71 (0.08)	0.71 (0.07)	0.71 (0.07)	0.77 (0.04)	0.78 (0.05)	0.82 (0.04)	0.78 (0.07)	0.84 (0.07)	0.81 (0.05)	0.8 (0.04)	0.87 (0.02)	0.86 (0.02)	0.87 (0.02)	0.87 (0.02)	0.79 (0.04)	0.84 (0.03)	0.84 (0.03)	0.84 (0.04)	0.88 (0.02)	0.87 (0.03)	0.88 (0.02)	0.88 (0.02)	0.73 (0.05)	0.72 (0.06)	0.72 (0.06)	0.72 (0.06)
	g-means	0.72 (0.06)	0.71 (0.06)	0.71 (0.06)	0.71 (0.06)	0.7 (0.06)	0.78 (0.02)	0.78 (0.06)	0.78 (0.05)	0.82 (0.04)	0.78 (0.03)	0.82 (0.03)	0.84 (0.02)	0.83 (0.02)	0.84 (0.02)	0.84 (0.02)	0.78 (0.04)	0.82 (0.03)	0.82 (0.03)	0.82 (0.03)	0.85 (0.02)	0.85 (0.03)	0.85 (0.02)	0.85 (0.02)	0.73 (0.04)	0.72 (0.05)	0.72 (0.05)	0.72 (0.05)
	AUC	0.72 (0.06)	0.71 (0.06)	0.71 (0.06)	0.71 (0.06)	0.79 (0.05)	0.82 (0.03)	0.84 (0.04)	0.85 (0.04)	0.88 (0.04)	0.85 (0.03)	0.89 (0.02)	0.89 (0.02)	0.89 (0.02)	0.9 (0.01)	0.9 (0.02)	0.85 (0.03)	0.88 (0.03)	0.87 (0.03)	0.87 (0.03)	0.9 (0.02)	0.91 (0.01)	0.91 (0.01)	0.91 (0.01)	0.79 (0.04)	0.75 (0.05)	0.75 (0.05)	0.75 (0.05)
Sotiriou Grade	PA	0.6 (0.05)	0.6 (0.05)	0.6 (0.04)	0.6 (0.06)	0.64 (0.04)	0.65 (0.04)	0.66 (0.03)	0.64 (0.05)	0.65 (0.04)	0.6 (0.04)	0.63 (0.03)	0.67 (0.02)	0.66 (0.03)	0.66 (0.02)	0.66 (0.03)	0.62 (0.04)	0.63 (0.03)	0.64 (0.03)	0.64 (0.03)	0.66 (0.02)	0.65 (0.03)	0.67 (0.03)	0.66 (0.03)	0.63 (0.04)	0.64 (0.03)	0.64 (0.03)	0.65 (0.03)
	PA_1	0.58 (0.08)	0.59 (0.08)	0.59 (0.07)	0.59 (0.08)	0.63 (0.08)	0.63 (0.05)	0.67 (0.05)	0.62 (0.07)	0.66 (0.06)	0.6 (0.07)	0.62 (0.05)	0.66 (0.04)	0.66 (0.04)	0.65 (0.04)	0.65 (0.04)	0.61 (0.06)	0.62 (0.05)	0.63 (0.04)	0.64 (0.04)	0.65 (0.04)	0.64 (0.05)	0.66 (0.04)	0.65 (0.05)	0.62 (0.05)	0.63 (0.06)	0.63 (0.05)	0.64 (0.05)
	PA_2	0.61 (0.07)	0.6 (0.07)	0.61 (0.05)	0.61 (0.07)	0.66 (0.03)	0.67 (0.05)	0.65 (0.04)	0.65 (0.06)	0.64 (0.06)	0.61 (0.06)	0.64 (0.05)	0.67 (0.04)	0.66 (0.04)	0.67 (0.04)	0.66 (0.04)	0.62 (0.06)	0.65 (0.04)	0.64 (0.04)	0.65 (0.04)	0.67 (0.03)	0.67 (0.04)	0.68 (0.03)	0.67 (0.03)	0.64 (0.06)	0.65 (0.04)	0.65 (0.05)	0.65 (0.05)
	g-means	0.59 (0.05)	0.6 (0.05)	0.59 (0.04)	0.59 (0.06)	0.64 (0.05)	0.65 (0.04)	0.66 (0.03)	0.63 (0.05)	0.65 (0.04)	0.6 (0.04)	0.63 (0.03)	0.67 (0.03)	0.66 (0.03)	0.66 (0.02)	0.65 (0.03)	0.62 (0.04)	0.63 (0.03)	0.64 (0.03)	0.64 (0.03)	0.66 (0.02)	0.65 (0.03)	0.66 (0.03)	0.66 (0.03)	0.63 (0.04)	0.64 (0.04)	0.64 (0.03)	0.64 (0.04)
	AUC	0.6 (0.05)	0.6 (0.05)	0.6 (0.04)	0.6 (0.06)	0.66 (0.04)	0.69 (0.03)	0.7 (0.03)	0.69 (0.05)	0.7 (0.05)	0.65 (0.05)	0.69 (0.03)	0.72 (0.03)	0.73 (0.03)	0.73 (0.03)	0.73 (0.03)	0.66 (0.04)	0.69 (0.04)	0.69 (0.03)	0.69 (0.03)	0.72 (0.04)	0.73 (0.04)	0.74 (0.03)	0.74 (0.03)	0.68 (0.04)	0.69 (0.04)	0.67 (0.04)	0.66 (0.04)
